# Design, Synthesis, Biological Evaluation and In Silico Studies of Pyrazole-Based NH_2_-Acyl Oseltamivir Analogues as Potent Neuraminidase Inhibitors

**DOI:** 10.3390/ph14040371

**Published:** 2021-04-16

**Authors:** Jiqing Ye, Lin Lin, Jinyi Xu, Paul Kay-sheung Chan, Xiao Yang, Cong Ma

**Affiliations:** 1 State Key Laboratory of Chemical Biology and Drug Discovery, Department of Applied Biology and Chemical Technology, The Hong Kong Polytechnic University, Kowloon, Hong Kong, China; jiqing.ye@connect.polyu.hk; 2 Department of Microbiology, The Chinese University of Hong Kong, Prince of Wales Hospital, Shatin, Hong Kong, China; linlin@cuhk.edu.hk (L.L.); paulkschan@cuhk.edu.hk (P.K.-s.C.); 3 State Key Laboratory of Natural Medicines and Department of Medicinal Chemistry, China Pharmaceutical University, 24 Tong Jia Xiang, Nanjing 210009, China; jinyixu@cpu.edu.cn; 4 Stanley Ho Centre for Emerging Infectious Diseases, The Chinese University of Hong Kong, Shatin, Hong Kong, China

**Keywords:** influenza virus, neuraminidase inhibitor, 150-cavity, oseltamivir derivatives, pyrazole

## Abstract

Oseltamivir represents one of the most successful neuraminidase (NA) inhibitors in the current anti-influenza therapy. The 150-cavity of NA was identified as an additional binding pocket, and novel NA inhibitors have been designed to occupy the 150-cavity based on the structure information of oseltamivir carboxylate (**OC**) in complex with NA. In this study, a series of C-5-NH_2_-acyl derivatives of **OC** containing the pyrazole moiety were synthesized. Several derivatives exhibited substantial inhibitory activity against NA. Moreover, in silico ADME evaluation indicated that the derivatives were drug-like with higher oral absorption rates and greater cell permeability than **OC**. Additionally, molecular docking studies revealed that the derivatives interacted with both the NA enzyme active site and 150-cavity as expected. The results provided useful information for further structural optimization of **OC**.

## 1. Introduction

Seasonal flu epidemics and pandemics caused substantial mortality and economic loss. Reports showed that up to 0.65 million respiratory deaths per annum are attributed to seasonal flu globally [1]. According to the antigenic differences in their nucleoprotein (NP) and matrix protein (M1), influenza viruses are classified as types A, B, and C [2]. Different subtypes of influenza A virus were named according to two important surface antigenic glycoproteins, hemagglutinin (HA) and neuraminidase (NA) [3]. So far, 18 HA subtypes (H1–18) and 11 NA subtypes (N1–N11) were identified [4], in which several different subtypes caused severe pandemics in the last century, including the 1918 Spanish flu (H1N1), 1957 Asian flu (H2N2), 1968 Hong Kong flu (H3N2) [5], and 2009 swine flu (H1N1 pdm09) [6].

**Figure 1:**
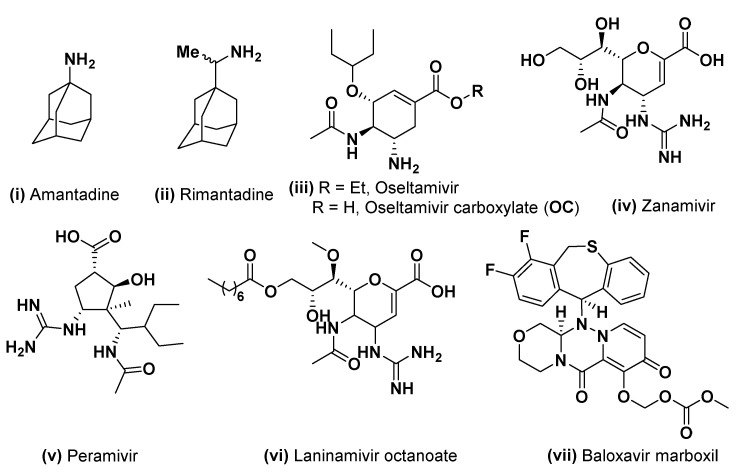
Representative anti-influenza drugs. M2 channel inhibitors: (**i**,**ii**); NAIs: (**iii**–**vi**) and cap-dependent endonuclease inhibitor (**vii**).

Presently, there are three classes of anti-influenza drugs, consisting of the M2 channel inhibitors, the neuraminidase inhibitors (NAIs) [7], and the recently approved cap-dependent endonuclease inhibitor baloxavir marboxil [8] (Figure 1), although the viral resistance to all the drugs has been reported [9]. NAIs have been approved as antivirals in clinical use for about 20 years, and they have a mode of action of disrupting the release of nascent viruses from host cells. Amongst all the NAIs, oseltamivir phosphate (**OP**, ethyl ester prodrug) has become a first-line therapy since its approval in 1999 [10].

**Figure 2:**
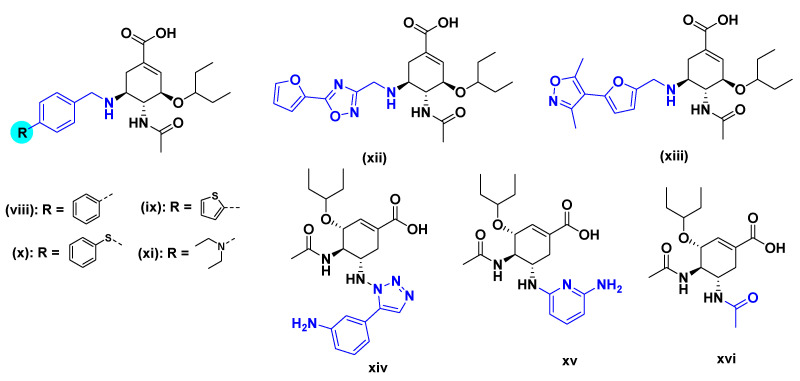
Representative C-5-NH_2_ modified **OC** derivatives designed as 150-cavity binders: secondary amine derivatives (**viii**–**xiii**), triazolylated analogue (**xiv**), pyridyl analogue (**xv**) and acyl analogue (**xvi**).

Phylogenetically, NAs can be divided into two groups: group 1 contains N1, N4, N5, and N8, and group 2 contains N2, N3, N6, N7, and N9 [11]. Based on the structural observations, Russel et al. disclosed that group-1 neuraminidases contain a so-called 150-cavity adjacent to their active sites, which suggests a new binding pocket for drug design [12]. Besides, it was also reported that N2 NA could be induced to an open position by the binding of oseltamivir carboxylate to NA [13]. Crystal structures of **OC** in complex with NAs indicated that the C-5-NH_2_ group of **OC** close to the 150-cavity of NA might be a possible modification site. On this basis, several C-5-NH_2_ modified **OC** derivatives have been reported as 150-cavity binders, including second amine derivatives (viii–xiii) [14,15,16,17]. These derivatives inhibited NA at the nM level, and most of them possessed the selectivity against group 1 NAs. Other derivatives, such as triazolylated analogue (xiv) [18], pyridyl analogue (xv) [19], and C-5-NH_2_-acyl analogue (xvi) (Figure 2) [20], showed moderate activity against NA.

Previously, we designed and synthesized a class of heterocycle-bearing secondary amine derivatives of **OC** for probing the 150-cavity [17]. Results indicated that the introduction of heterocyclic side chains could maintain the antiviral activity of **OC** with appropriate ADME properties. In this study, in an attempt to develop novel NA 150-cavity binders, we intended to modify the C-5-NH_2_ group of **OC** by incorporating pyrazole moiety. A series of pyrazole-containing C-5-NH_2_-acyl **OC** analogues were prepared, and their inhibitory activities to NA of a clinically isolated A/H3N2 influenza virus strain from Hong Kong were evaluated.

## 2. Results and Discussion

### 2.1. Design of Novel Oseltamivir Analogues

**Figure 3:**
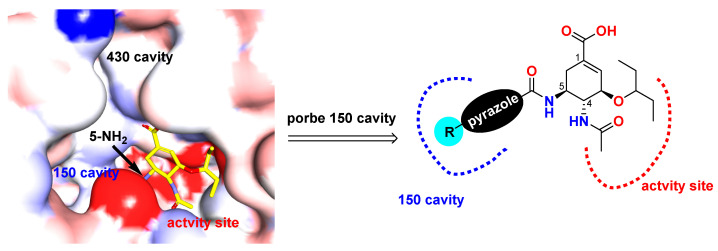
Design of the pyrazole-based C-5-NH_2_-acyl oseltamivir analogues.

The reported co-crystal structure of **OC** in complex with NA (PDB: 2HU0) showed that C-5-NH_2_ of **OC** is adjacent to the 150-cavity, which suggests that the C-5-NH_2_ may become a modification site towards the discovery of novel NA inhibitors. Based on this information, attempts on the development of 150-cavity binders as **OC** inhibitors with increased activity or overcome drug resistance were carried out. Herein, we designed, synthesized, and tested a series of C-5-NH_2_-acyl analogues of **OC** containing the pyrazole moiety through an amide bond with different substitutions on pyrazole (Figure 3).

### 2.2. Chemistry

**Scheme 1:**
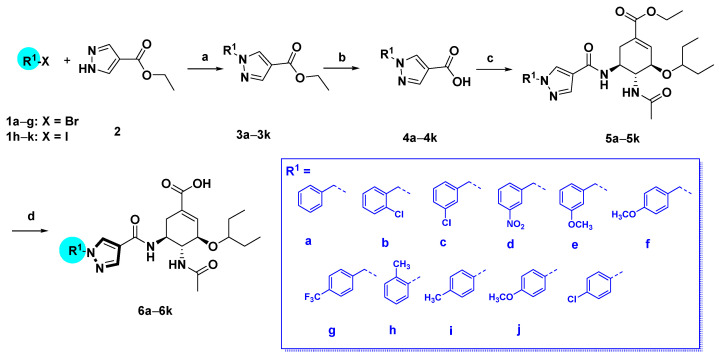
Synthetic route of compounds **6a**–**k**. Reagents and conditions: (**a**) for **3a**–**g**: **1a**–**g**, K_2_CO_3_, acetone, reflux, overnight, 60–90%; for **3h**–**k**: **1h**–**k**, CuI, *trans*-*N,N′*-dimethyl-1,2-cyclohexanediamine, K_2_CO_3_, Tol., 110 °C, 24 h, 30–55%. (**b**) NaOH, MeOH/H_2_O, 50 °C; (**c**) oseltamivir phosphate, HATU, DIPEA, DCM, rt, 60–90%; (**d**) NaOH, MeOH/H_2_O, 50 °C, 40–70%.

**Scheme 2:**
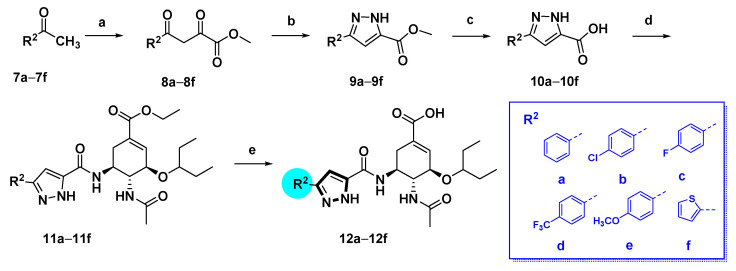
Synthetic route of compounds **12a**–**f**. Reagents and conditions: (**a**) Dimethyl oxalate, *t*-BuOK, Tol, rt; (**b**) NH_2_NH_2_⋅H_2_O, AcOH, 90 °C, 4 h, 70–80%; (**c**) NaOH, MeOH/H_2_O, 50 °C, overnight, 80–90%; (**d**) oseltamivir phosphate, HATU, DIPEA, DCM, rt, 40–80%; (**e**) NaOH, MeOH/H_2_O, 50 °C, 50–70%.

**Scheme 3:**
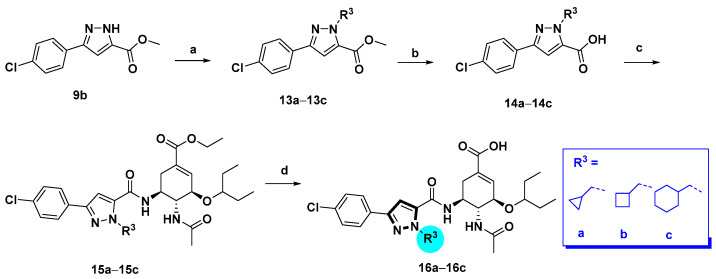
Synthetic route of compounds **16a**-**c**. Reagents and conditions: (**a**) R^3^Br, K_2_CO_3_, acetone, reflux, 60–70%; (**b**) NaOH, MeOH/H_2_O, 50 °C, 80–90%; (**c**) oseltamivir phosphate, HATU, DIPEA, DCM, rt, 40–90%; (**d**) NaOH, MeOH/H_2_O, 50 °C, 40–60%.

**Scheme 4:**
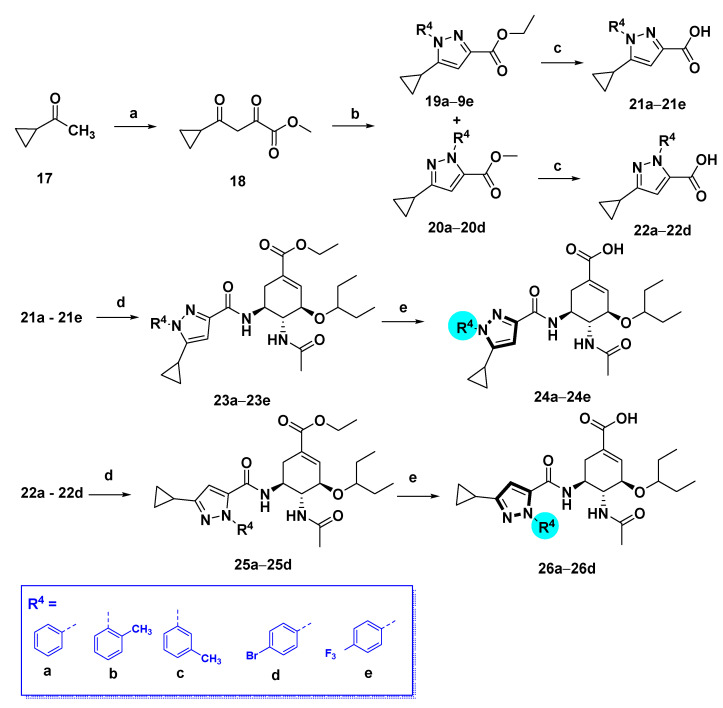
Synthetic route of compounds **24a**–**e** and **26a**–**d**. Reagents and conditions: (**a**) Dimethyl oxalate, *t*-BuOK, Tol, rt; (**b**) R^4^NHNH_2_⋅HCl, EtOH, reflux, 5 h, **19a**–**e**: 40–80%, **20a**–**d**: 15–30%; (**c**) NaOH, MeOH/H_2_O, 50 °C, 80–90%; (**d**) HATU, DIPEA, DCM, rt, 50–80%; (**e**) NaOH, MeOH/H_2_O, 50 °C, 40–70%.

The pyrazole-based C-5-NH_2_-acyl **OC** derivatives were synthesized following the synthetic routes described in Scheme 1, Scheme 2, Scheme 3 and Scheme 4.

As shown in Scheme 1, we first prepared a series of 1*H*-pyrazole-4-carboxamide derivatives **6a**–**k**. Ethyl 1*H*-pyrazole-4-carboxylate 2 reacted with various aryl bromides in the presence of K_2_CO_3_ [21] to furnish **3a**–**g**. The arylated derivatives **3h**–**k** were prepared by the coupling of aryl iodides with compound 2 catalyzed by the CuI-diamine system with K_2_CO_3_ as the base [21]. Hydrolysis of the esters **3a**–**k** occurred under basic conditions to afford 1*H*-pyrazole-4-carboxylic acid derivatives **4a**–**k**. The condensation of oseltamivir phosphate with substituted pyrazolecarboxylic acids **4a**–**k** using the HATU/DIPEA methodology provided **5a**–**k**. Finally, hydrolysis of ethyl ester gave compounds **6a**–**k**.

The 1*H*-pyrazole-5-carboxamide derivatives **12a**–**f** were synthesized as illustrated in Scheme 2. Treatment of methyl ketones **7a**–**f** with dimethyl oxalate in the presence of potassium *tert*-butoxide afforded intermediates **8a**–**f**, which were subjected to cyclization with hydrazine at 50 °C in AcOH to give methyl 1*H*-pyrazole-5-carboxylate derivatives **9a**–**f** [22], followed by hydrolysis to furnish the 1*H*-pyrazole-5-carboxylic acid derivatives **10a**–**f**. The condensation of **10a**–**f** with oseltamivir phosphate under the HATU/DIPEA condition provided **11a**–**f**. After hydrolysis of ethyl ester, compounds **12a**–**f** were obtained.

Alkylation at the pyrazole *N*-1 position of methyl 3-(4-chlorophenyl)-1*H*-pyrazole-5-carboxylate (**9b**) was accomplished as outlined in Scheme 3. Compound **9b** was respectively alkylated with several alkyl bromides with diverse ring sizes in the presence of K_2_CO_3_, followed by hydrolysis of the esters to afford carboxylic acids **14a**–**c**. Subsequently, condensation of acids **14a**–**c** with oseltamivir phosphate gave compounds **15a**–**c**, which underwent facile hydrolysis to provide compounds **16a**–**c**.

1*H*-pyrazole-3-carboxamide derivatives **24a**–**e** and 1*H*-pyrazole-5-carboxamide derivatives **26a**–**d** were prepared as shown in Scheme 4. Claisen condensation of commercially available 1-cyclopropylethan-1-one **17** with dimethyl oxalate afforded diketoester **18**, which underwent cyclization with substituted hydrazines to give both the corresponding 1,5-disubstituted pyrazole-3-carboxylic acid esters **19a**–**e**, and the isomeric derivatives, 1,3-disubstituted pyrazole-5-carboxylic acid esters **20a**–**d**. The latter could be isolated and purified by column chromatography [23,24]. The esters were hydrolyzed by aqueous sodium hydroxide solution to afford the corresponding carboxylic acids **21a**–**e** and **22a**–**d**. These carboxylic acids reacted with oseltamivir phosphate to furnish compounds **23a**–**e** and **25a**–**d**, respectively. After hydrolysis, compounds **24a**–**e** and **26a**–**d** were obtained.

### 2.3. Neuraminidase Enzyme Inhibitory Assay

A clinically isolated A/H3N2 strain from Hong Kong was cultivated with host cell cultures followed by lysis. All of the newly synthesized 29 compounds were evaluated for their inhibitory activity to A/H3N2 NA with **OC** as the reference drug [17]. The results were shown in Table 1.

Regarding the first series **6a**–**k** with a *1H*-pyrazole-4-carboxamide moiety, most of the compounds displayed mild NA inhibitory activity. Overall, compounds **6a**–**g** containing benzyl or substituted benzyl groups showed less activity than the derivatives **6h**–**k** with substituted phenyls. Compound **6i** with a 4-methylphenyl group substituted at pyrazole *N*-1 position exhibited the highest NA inhibitory activity of 52.31%. Interestingly, compound **6j** with a strong electron-donating group (OCH_3_) and **6k** with a weak electron-withdrawing group (Cl) at the 4-position of the phenyl ring showed inferior inhibitory activities to **6i**. These results suggest that both steric and electronic properties need to be further investigated to clarify the properties for binding to the 150-cavity of NA.

For 1*H*-pyrazole-5-carboxamide derivatives **12a**–**f**, all the derivatives showed interesting inhibitory activity at 10 μM. The inhibitory activities of the compounds increased with the electron-withdrawing capability of the halide groups at 4-position of the phenyl ring (**12c** > **12b** > **12a**; **12d** >**12e**). Compound **12c** with the 4-fluorophenyl group was the most active compound in this series, with the percentages of inhibition of 56.45%.

The alkylation of the *N*-1 position of the pyrazole moiety of compound **12b** provided compounds **16a**–**c**. Results demonstrated that the cyclohexylmethyl group at *N*-1 of pyzarole (**16c**) was preferred. **16c** showed similar inhibitory activity to **12b**, and the decreased ring sizes in **16a** and **16b** caused inferior activities.

1*H*-pyrazole-3-carboxamide derivatives **24a**–**e** and their isomers 1*H*-pyrazole-5-carboxamide derivatives **26a**–**d** displayed diverse NA inhibitory activities from 30% to 72%. When the substituents at *para-* or *meta-* positions (e.g., **24c**–**e**, **26c**, and **26d**), the inhibitory potencies were similar to the non-substituted benzene derivatives **24a** or **26a**. Compounds **24b** and its isomer **26b** with 2-methylphenyl substituted on the pyrazole ring exhibited the most potent inhibitory activity of 60.91% and 72.80% at 10 μM, respectively. The outstanding inhibitory activity of both compounds suggests that the *ortho* effect of the substitutions might be beneficial to the activity.

Briefly, the structure-activity relationship (SAR) discussed above showed that 1*H*-pyrazole-5-carboxamide or 1*H*-pyrazole-3-carboxamide were the optimal linker fragments, and they provided different electronic properties from the substitutions to the *N* atom of the pyrazole. Together with our previous study [17], the SAR obtained here will guide the further design of more potent NA inhibitors.

Overall, most of the pyrazole-based C-5-NH_2_-acyl analogues of **OC** inhibited the enzyme activity of A/H3N2 NA. Compounds **6i**, **12c**, **24b**, and **26b** represented the most active compounds in each series. Their IC_50_ values against NA were then determined. As shown in Table 2, compounds **6i**, **12c**, **24b**, and **26b** exhibited moderate NA inhibitory activity, among which compound **12c** and **26b** displayed the best NA inhibitory activity with an IC_50_ value of 6.98 ± 0.08 μM and 12.27 ± 0.14 μM, respectively. Interestingly, **24b** and **26b** against NA at 10 μM exhibited to be more potent than **12c**, but compound **12c** inhibited NA with a lower IC_50_ value, probably because **12c** may elicit stronger inhibitory activity at lower concentrations.

### 2.4. In Silico ADME Prediction

To further explore the druggability of these **OC** derivatives, the ADME properties were predicted by Qikprop (Maestro 10.2) [17]. The ADME descriptors of compounds **6i**, **12c, 24b**, and **26b** were calculated, including octanol-water partitioning coefficient (QPlogPo/w), aqueous solubility (QPlogS), binding to human serum albumin (QPlogKhsa), brain/blood partition coefficient (QPlogBB), number of likely metabolic reactions (#metab), central nervous system activity (CNS), predicted IC_50_ value for the blockage of HERG K^+^ channels (QPloghERG), apparent Caco-2 cell permeability (QPPCaco), apparent MDCK cell permeability (QPPMDCK), and human oral absorption.

As shown in Table 3, the four C5-NH-acyl derivatives were predicted to be significantly more lipophilic with lower aqueous solubility in comparison with **OC**. Moreover, the percentages of human oral absorption of the new derivatives were 60~70%, higher than 32.7% for **OC**. Meanwhile, QPPCaco and QPPMDCK of the derivatives were predicted to be 3- or 7-fold higher than that of **OC**. These data suggest that our derivatives could be well absorbed by the intestine and thereof could be orally administrated directly instead of converting to the ester prodrug as **OC**. Other parameters such as QPlogBB, CNS, QPloghERG, and #metab remained within the standard ranges which suggests these derivatives should be metabolically stable and nontoxic to both the nervous system and heart.

### 2.5. Molecular Docking Studies

**Figure 4:**
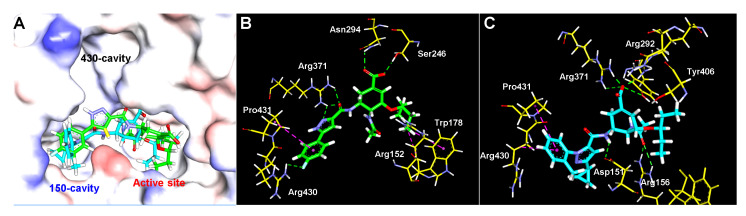
(**A**) Binding positions of compounds **12c** (green) and **26b** (cyan) to NA (PDB: 2HU0); Detailed interactions of **12c** (**B**) and **26b** (**C**) with NA: Hydrogen/ionic bonding interactions (green) and hydrophobic interactions (magenta).

As described previously, molecular docking studies were performed to reveal the potential interactions between NA and the newly designed **OC** derivatives. All the final compounds were docked with the crystal structure of NA (PDB: 2HU0) using Glide of Maestro 10.2 [17]. Results showed that most of the derivatives fitted into the NA active site with their OC motif and occupied the 150-cavity with the substituted pyrazole moieties as designed (Figure S1 and Figure 4A).

Details of the interactions between compound **12c** and NA are shown in Figure 4B and the key H-bonds were summarized in Table 4. The **OC** part of **12c** formed hydrogen bonding and hydrophobic interactions with the NA active site, including hydrogen bonds between the carboxylic acid group and residues Asn294 and Ser246, and hydrophobic interactions between the pentan-3-yloxy group and residues Arg152 and Trp178. In addition, the 5-(3-(4-fluorophenyl)-1*H*-pyrazole-5-carboxamide side chain formed hydrogen bonds with residues Arg371 and Arg430, and hydrophobic interaction with Pro431.

**26b** interacts with some different residues in the NA active site when compared with compound **12c** (Figure 4C, Table 4). The carboxylic group formed ionic bonding interactions with residue Arg292 and Arg371, and hydrogen bonding with Tyr406. Additionally, the C-4 amide group contacted Arg156 via two hydrogen bonds. These changes might enhance the stability of the **12c**-NA complex. Finally, hydrophobic interactions were formed between the phenyl ring and residues Pro431 and Arg430. Overall, the additional interactions of OC derivatives with NA may improve the binding affinity.

## 3. Materials and Methods

### 3.1. Biology

#### NA Enzyme Inhibitory Assay

The NA inhibitory assay was performed using the commercially available NA-Fluor™ Influenza Neuraminidase Assay Kit as described previously [17].

### 3.2. Chemistry

#### 3.2.1. General Methods

Starting materials and reagents, unless otherwise stated, were commercial grade and used without further purification. Thin-layer chromatography (TLC) on glass sheets (Silica gel F_254_) was visualized under UV light and used to monitor all the reactions. Column chromatography was carried out using silica gel (200–300 mesh). Compound structures were confirmed by ^1^H NMR (400 MHz) and ^13^C NMR (100 MHz) spectra which were acquired using a BRUKER AVANCE-III spectrometer. NMR chemical shifts were given in *δ* (ppm) and coupling constants (*J*) in Hz. The solvents for NMR were DMSO-*d*_6_ (*δ* 2.50 for ^1^H) or chloroform (*δ* 7.26 for ^1^H). High-resolution MS spectra were measured using a Micromass^®^ Q-ToF2 mass spectrometer.

#### 3.2.2. General Procedures for the Synthesis of Compounds **3a**–**k**

For compounds **3a**–**g**: A mixture of ethyl-4-pyrazole carboxylate (280 mg, 2 mmol), K_2_CO_3_ (1.38 g, 10 mmol), and a bromomethyl arene (2.4 mmol) in acetone (10 mL) was heated to reflux for 12 h. After cooling to room temperature, K_2_CO_3_ was filtered off and the filtrate was concentrated, purified by column chromatography, eluting with a gradient of hexane/ethyl acetate (15:1 to 6:1) to provide compounds **3a**–**g**.

For compounds **3h**–**3k**: To a Schlenk tube were added ethyl-4-pyrazole carboxylate (280 mg, 2 mmol), an aryl iodide (2.2 mmol), CuI (76 mg, 0.4 mmol), K_2_CO_3_ (580 mg, 4.2 mmol) and *trans*-*N,N′*-dimethyl-1,2-cyclohexanediamine (113 mg, 0.8 mmol) and toluene (5 mL). The reaction tube was charged with N_2_ and immersed in a preheated oil bath for stirring at 110 °C for 24 h. The reaction mixture was concentrated, and the resulting residue was purified by column chromatography, eluting with a gradient of hexane/ethyl acetate (20:1 to 10:1) to provide the desired products.

#### 3.2.3. General Procedure for the Synthesis of Compounds **4a**–**k**

Each ethyl ester of **3a**–**k** was dissolved in MeOH (10 mL), 1 M NaOH aqueous solution (2 mL) was added. The mixture was stirred at 50 °C overnight. The solvent was removed under reduced pressure and the residue was diluted with 10 mL water and washed with DCM. The aqueous phase was acidified by 1 M HCl to precipitate the product which was collected by filtration and dried in vacuo. The products were used directly for the next step of the reaction.

#### 3.2.4. General Procedure for the Synthesis of Compounds **5a**–**k**

A mixture of oseltamivir phosphate (82 mg, 0.2 mmol), each pyrazole-4-carboxylic acid derivative of **4a**–**k** (0.24 mmol), HATU (115 mg, 0.3 mmol) and DIPEA (83 μL, 0.5 mmol) in DCM (10 mL) was stirred at room temperature overnight. The reaction was quenched by saturated NaHCO_3_ aqueous solution and the resulting mixture was extracted with DCM (20 mL × 3). The combined organic phases were washed with brine, dried over Na_2_SO_4_, concentrated, and purified by silica gel column chromatography, eluting with a gradient of DCM/MeOH (50:1 to 30:1) to afford the titled compound.

#### 3.2.5. General Procedure for the Synthesis of Compounds **6a**–**k**

Each compound of **5a**–**k** (0.1 mmol) was dissolved in MeOH (10 mL), 1 M NaOH aqueous solution (2 mL) was added. The mixture was heated to 50 °C overnight. The solvent was removed under reduced pressure and the residue was diluted with 10 mL water and washed with DCM (10 mL × 2). The aqueous phase was acidified by 1 M HCl to precipitate the product which was collected by filtration and dried in vacuo.

#### 3.2.6. (*3R,4R,5S*)-4-acetamido-5-(1-benzyl-1*H*-pyrazole-4-carboxamido)-3-(pentan-3-yloxy) cyclohex-1-ene-1-carboxylic acid (**6a**)

Yield 59%; white solid, mp 273–275 °C. ^1^H NMR (400 MHz, DMSO-*d*_6_) *δ* 12.60 (s, 1H), 8.18 (s, 1H), 7.97–7.80 (m, 2H), 7.79 (s, 1H), 7.42–7.28 (m, 3H), 7.25 (d, *J* = 6.7 Hz, 2H), 6.63 (s, 1H), 5.35 (s, 2H), 4.08 (ddd, *J* = 19.4, 17.3, 9.0 Hz, 2H), 3.83 (dd, *J* = 19.9, 8.9 Hz, 1H), 3.45–3.30 (m, 1H), 2.55 (d, *J* = 5.1 Hz, 1H), 2.38–2.24 (m, 1H), 1.68 (s, 3H), 1.53–1.30 (m, 4H), 0.84 (t, *J* = 7.3 Hz, 3H), 0.77 (t, *J* = 7.3 Hz, 3H). ^13^C NMR (100 MHz, DMSO-*d*_6_) *δ* 170.3, 167.9, 162.0, 139.2, 138.3, 137.4, 131.8, 129.8, 129.0, 128.3, 128.2, 119.1, 81.6, 75.5, 55.5, 54.6, 48.3, 30.8, 26.3, 25.8, 23.2, 9.8, 9.5. HRMS (ESI): calcd for C_25_H_31_N_4_O_5_, [M-H]^−^ 467.2300, found 467.2309.

#### 3.2.7. (*3R,4R,5S*)-4-acetamido-5-(1-(2-chlorobenzyl)-1*H*-pyrazole-4-carboxamido)-3-(pentan-3-yloxy) cyclohex-1-ene-1-carboxylic acid (**6b**)

Yield 52%; white solid, mp 265–267 °C. ^1^H NMR (400 MHz, DMSO-*d*_6_) *δ* 8.16 (s, 1H), 7.94–7.83 (m, 2H), 7.82 (s, 1H), 7.50 (dd, *J* = 7.6, 1.4 Hz, 1H), 7.42–7.31 (m, 2H), 7.12 (dd, *J* = 7.3, 1.8 Hz, 1H), 6.63 (s, 1H), 5.46 (s, 2H), 4.14 (d, *J* = 8.1 Hz, 1H), 4.11–4.02 (m, 1H), 3.84 (dd, *J* = 20.0, 9.0 Hz, 1H), 3.45–3.30 (m, 1H), 2.57–2.51 (m, 1H), 2.32 (dd, *J* = 17.5, 10.4 Hz, 1H), 1.68 (s, 3H), 1.52–1.30 (m, 4H), 0.84 (t, *J* = 7.3 Hz, 3H), 0.77 (t, *J* = 7.3 Hz, 3H). ^13^C NMR (100 MHz, DMSO-*d*_6_) *δ* 170.3, 167.7, 161.9, 139.4, 138.2, 134.6, 132.9, 132.2, 130.8, 130.4, 130.0, 129.8, 128.0, 119.1, 81.6, 75.6, 54.6, 53.2, 48.4, 30.8, 26.3, 25.8, 23.2, 9.8, 9.5. HRMS (ESI): calcd for C_25_H_30_ClN_4_O_5_, [M-H]^−^ 501.1910, found 501.1912.

#### 3.2.8. (*3R,4R,5S*)-4-acetamido-5-(1-(3-chlorobenzyl)-1*H*-pyrazole-4-carboxamido)-3-(pentan-3-yloxy) cyclohex-1-ene-1-carboxylic acid (**6c**)

Yield 63%; white solid, mp 245–247 °C. ^1^H NMR (400 MHz, DMSO-*d*_6_) *δ* 12.62 (s, 1H), 8.21 (s, 1H), 7.84 (dd, *J* = 12.9, 9.2 Hz, 2H), 7.80 (s, 1H), 7.44–7.34 (m, 2H), 7.29 (s, 1H), 7.25–7.17 (m, 1H), 6.63 (s, 1H), 5.38 (s, 2H), 4.17–4.01 (m, 2H), 3.83 (dd, *J* = 19.9, 9.1 Hz, 1H), 3.45–3.30 (m, 1H), 2.55–2.51 (m, 1H), 2.32 (dd, *J* = 17.6, 10.4 Hz, 1H), 1.67 (s, 3H), 1.53–1.34 (m, 4H), 0.84 (t, *J* = 7.3 Hz, 3H), 0.76 (t, *J* = 7.3 Hz, 3H). ^13^C NMR (100 MHz, DMSO-*d*_6_) *δ* 170.2, 167.7, 161.9, 139.9, 139.4, 138.2, 133.6, 132.0, 131.0, 129.8, 128.2, 128.0, 126.0, 119.3, 81.6, 75.6, 54.7, 54.6, 48.4, 30.8, 26.3, 25.8, 23.2, 9.8, 9.5. HRMS (ESI): calcd for C_25_H_30_ClN_4_O_5_, [M-H]^−^ 501.1910, found 501.1912.

#### 3.2.9. (*3R,4R,5S*)-4-acetamido-5-(1-(3-nitrobenzyl)-*1H*-pyrazole-4-carboxamido)-3-(pentan-3-yloxy) cyclohex-1-ene-1-carboxylic acid (**6d**)

Yield 59%; white solid, mp 236–238 °C. ^1^H NMR (400 MHz, DMSO-*d*_6_) *δ* 8.28 (s, 1H), 8.22–8.15 (m, 1H), 8.12 (s, 1H), 7.94–7.79 (m, 3H), 7.71 (d, *J* = 7.6 Hz, 1H), 7.67 (t, *J* = 7.8 Hz, 1H), 6.63 (s, 1H), 5.54 (s, 2H), 4.17–4.01 (m, 2H), 3.84 (dd, *J* = 19.9, 9.0 Hz, 1H), 3.45–3.30 (m, 1H), 2.60–2.51 (m, 1H), 2.32 (dd, *J* = 17.5, 10.4 Hz, 1H), 1.68 (s, 3H), 1.51–1.31 (m, 4H), 0.84 (t, *J* = 7.3 Hz, 3H), 0.76 (t, *J* = 7.3 Hz, 3H). ^13^C NMR (100 MHz, DMSO-*d*_6_) *δ* 170.3, 167.8, 161.9, 148.3, 139.7, 139.6, 138.1, 134.9, 132.3, 130.7, 129.9, 123.3, 122.8, 119.4, 81.6, 75.6, 54.6, 54.4, 48.4, 30.8, 26.3, 25.7, 23.1, 9.8, 9.5. HRMS (ESI): calcd for C_25_H_30_N_5_O_7_, [M-H]^−^ 512.2151, found 512.2156.

#### 3.2.10. (*3R,4R,5S*)-4-acetamido-5-(1-(3-methoxybenzyl)-*1H*-pyrazole-4-carboxamido)-3-(pentan-3-yloxy) cyclohex-1-ene-1-carboxylic acid (**6e**)

Yield 62%; white solid, mp 259–261 °C. ^1^H NMR (400 MHz, DMSO-*d*_6_) *δ* 8.17 (s, 1H), 7.88 (d, *J* = 9.1 Hz, 1H), 7.82 (d, *J* = 8.9 Hz, 1H), 7.79 (s, 1H), 7.27 (t, *J* = 7.8 Hz, 1H), 6.88 (dd, *J* = 8.1, 2.1 Hz, 1H), 6.84–6.74 (m, 2H), 6.63 (s, 1H), 5.32 (s, 2H), 4.18–4.00 (m, 2H), 3.83 (dd, *J* = 20.0, 9.0 Hz, 1H), 3.73 (s, 3H), 3.45–3.30 (m, 1H), 2.59–2.50 (m, 1H), 2.37–2.27 (m, 1H), 1.67 (s, 3H), 1.51–1.28 (m, 4H), 0.84 (t, *J* = 7.3 Hz, 3H), 0.77 (t, *J* = 7.4 Hz, 3H). ^13^C NMR (100 MHz, DMSO-*d*_6_) *δ* 170.2, 167.7, 162.0, 159.8, 139.2, 138.9, 138.3, 131.8, 130.2, 129.8, 120.3, 119.1, 114.0, 113.6, 81.6, 75.6, 55.5, 55.4, 54.6, 48.3, 30.8, 26.3, 25.7, 23.1, 9.8, 9.5. HRMS (ESI): calcd for C_26_H_33_N_4_O_6_, [M-H]^−^ 497.2406, found 497.2410.

#### 3.2.11. (*3R,4R,5S*)-4-acetamido-5-(1-(4-methoxybenzyl)-1*H*-pyrazole-4-carboxamido)-3-(pentan-3-yloxy) cyclohex-1-ene-1-carboxylic acid (**6f**)

Yield 45%; white solid, mp 269–271 °C. ^1^H NMR (400 MHz, DMSO-*d*_6_) *δ* 8.11 (s, 1H), 7.87 (d, *J* = 9.1 Hz, 1H), 7.79 (d, *J* = 8.9 Hz, 1H), 7.76 (s, 1H), 7.24 (d, *J* = 8.5 Hz, 2H), 6.92 (d, *J* = 8.5 Hz, 2H), 6.63 (s, 1H), 5.26 (s, 2H), 4.14 (d, *J* = 8.0 Hz, 1H), 4.10–4.00 (m, 1H), 3.83 (dd, *J* = 19.9, 9.0 Hz, 1H), 3.74 (s, 3H), 3.45–3.30 (m, 1H), 2.63–2.45 (m, 1H), 2.34–2.20 (m, 1H), 1.68 (s, 3H), 1.53–1.30 (m, 4H), 0.84 (t, *J* = 7.3 Hz, 3H), 0.77 (t, *J* = 7.3 Hz, 3H). ^13^C NMR (100 MHz, DMSO-*d*_6_) *δ* 170.3, 167.7, 162.0, 159.4, 139.0, 138.2, 131.4, 129.9, 129.8, 129.2, 119.0, 114.4, 81.6, 75.6, 55.6, 55.0, 54.6, 48.3, 30.8, 26.3, 25.7, 23.2, 9.8, 9.5. HRMS (ESI): calcd for C_26_H_33_N_4_O_6_, [M-H]^−^ 497.2408, found 497.2410.

#### 3.2.12. (*3R,4R,5S*)-4-acetamido-3-(pentan-3-yloxy)-5-(1-(4-(trifluoromethyl) benzyl)-1*H*-pyrazole-4-carboxamido) cyclohex-1-ene-1-carboxylic acid (**6g**)

Yield 51%; white solid, mp 259–261 °C. ^1^H NMR (400 MHz, DMSO-*d*_6_) *δ* 8.24 (s, 1H), 7.86 (dd, *J* = 15.2, 10.8 Hz, 3H), 7.73 (d, *J* = 8.1 Hz, 2H), 7.44 (d, *J* = 8.0 Hz, 2H), 6.64 (s, 1H), 5.48 (s, 2H), 4.22–4.01 (m, 2H), 3.84 (dd, *J* = 19.8, 9.1 Hz, 1H), 3.40–3.30 (m, 1H), 2.60–2.51 (m, 1H), 2.32 (dd, *J* = 17.5, 10.4 Hz, 1H), 1.68 (s, 3H), 1.53–1.31 (m, 4H), 0.84 (t, *J* = 7.3 Hz, 3H), 0.77 (t, *J* = 7.3 Hz, 3H). ^13^C NMR (100 MHz, DMSO-*d*_6_) *δ* 170.3, 167.7, 161.9, 142.2, 139.5, 138.3, 132.3, 129.7, 128.85 (d, *J* = 31.7 Hz), 128.83, 126.0 (d, *J* = 3.2 Hz), 125.9 (d, *J* = 3.2 Hz), 124.6 (q, *J* = 260.3 Hz), 119.3, 81.6, 75.6, 54.8, 54.56, 48.4, 30.8, 26.3, 25.7, 23.2, 9.8, 9.5. HRMS (ESI): calcd for C_26_H_30_F_3_N_4_O_5_, [M-H]^−^ 535.2174, found 535.2178.

#### 3.2.13. (*3R,4R,5S*)-4-acetamido-3-(pentan-3-yloxy)-5-(1-(o-tolyl)-1*H*-pyrazole-4-carboxamido) cyclohex-1-ene-1-carboxylic acid (**6h**)

Yield 61%; white solid, mp 253–255 °C. ^1^H NMR (400 MHz, DMSO-*d*_6_) *δ* 8.34 (s, 1H), 8.02 (s, 1H), 7.93 (dd, *J* = 9.0, 4.2 Hz, 2H), 7.47–7.32 (m, 4H), 6.65 (s, 1H), 4.22–4.05 (m, 2H), 3.87 (dd, *J* = 20.0, 9.0 Hz, 1H), 3.45–3.30 (s, 1H), 2.58 (dd, *J* = 17.6, 5.0 Hz, 1H), 2.34 (dd, *J* = 17.4, 10.4 Hz, 1H), 2.21 (s, 3H), 1.71 (s, 3H), 1.52–1.34 (m, 4H), 0.85 (t, *J* = 7.3 Hz, 3H), 0.78 (t, *J* = 7.3 Hz, 3H). ^13^C NMR (100 MHz, DMSO-*d*_6_) *δ* 170.4, 167.7, 161.9, 140.0, 139.6, 138.3, 133.2, 132.8, 131.8, 129.8, 129.2, 127.3, 126.2, 119.8, 81.6, 75.6, 54.7, 48.5, 30.8, 26.3, 25.8, 23.2, 18.2, 9.9, 9.5. HRMS (ESI): calcd for C_25_H_31_N_4_O_5_, [M-H]^−^ 467.2300, found 467.2304.

#### 3.2.14. (*3R,4R,5S*)-4-acetamido-3-(pentan-3-yloxy)-5-(1-(p-tolyl)-1*H*-pyrazole-4-carboxamido) cyclohex-1-ene-1-carboxylic acid (**6i**)

Yield 45%; white solid, mp 282–284 °C. ^1^H NMR (400 MHz, DMSO-*d*_6_) *δ* 8.79 (s, 1H), 8.03 (s, 1H), 7.98 (d, *J* = 8.9 Hz, 1H), 7.92 (d, *J* = 9.1 Hz, 1H), 7.72 (d, *J* = 8.2 Hz, 2H), 7.33 (d, *J* = 8.1 Hz, 2H), 6.66 (s, 1H), 4.25–4.07 (m, 2H), 3.87 (dd, *J* = 19.8, 9.1 Hz, 1H), 3.45–3.30 (m, 1H), 2.58 (dd, *J* = 17.5, 4.8 Hz, 1H), 2.45–2.30 (m, 1H), 2.35 (s, 3H), 1.70 (s, 3H), 1.53–1.33 (m, 4H), 0.85 (t, *J* = 7.3 Hz, 3H), 0.78 (t, *J* = 7.3 Hz, 3H), 0.67–0.66 (m, 1H). ^13^C NMR (100 MHz, DMSO-*d*_6_) *δ* 170.3, 167.7, 161.7, 140.4, 138.3, 137.4, 136.9, 130.5, 129.8, 129.0, 120.8, 119.1, 81.6, 75.6, 54.7, 48.4, 30.8, 26.3, 25.8, 23.2, 20.9, 9.9, 9.5. HRMS (ESI): calcd for C_25_H_31_N_4_O_5_, [M-H]^−^ 467.2300, found 467.2306.

#### 3.2.15. (*3R,4R,5S*)-4-acetamido-5-(1-(4-methoxyphenyl)-1*H*-pyrazole-4-carboxamido)-3-(pentan-3-yloxy) cyclohex-1-ene-1-carboxylic acid (**6j**)

Yield 47%; white solid, mp 277–279 °C. ^1^H NMR (400 MHz, DMSO-*d*_6_) *δ* 8.72 (s, 1H), 8.01 (s, 1H), 7.94 (dd, *J* = 14.9, 9.2 Hz, 2H), 7.74 (d, *J* = 8.8 Hz, 2H), 7.08 (d, *J* = 8.7 Hz, 2H), 6.65 (s, 1H), 4.20–4.08 (m, 2H), 3.93–3.85 (m, 1H), 3.80 (s, 3H), 3.45–3.30 (m, 1H), 2.57 (dd, *J* = 17.4, 4.3 Hz, 1H), 2.41–2.30 (m, 1H), 1.70 (s, 3H), 1.51–1.33 (m, 4H), 0.85 (t, *J* = 7.2 Hz, 3H), 0.78 (t, *J* = 7.2 Hz, 3H). ^13^C NMR (100 MHz, DMSO-*d*_6_) *δ* 170.3, 167.8, 161.8, 158.6, 140.2, 138.1, 133.2, 130.0, 128.9, 120.8, 120.6, 115.2, 81.6, 75.6, 55.9, 54.7, 48.4, 30.9, 26.3, 25.8, 23.2, 9.9, 9.5. HRMS (ESI): calcd for C_25_H_31_N_4_O_6_, [M-H]^−^ 483.2249, found 483.2252.

#### 3.2.16. (*3R,4R,5S*)-4-acetamido-5-(1-(4-chlorophenyl)-1*H*-pyrazole-4-carboxamido)-3-(pentan-3-yloxy) cyclohex-1-ene-1-carboxylic acid (**6k**)

Yield 55%; white solid, mp 295–297 °C. ^1^H NMR (400 MHz, DMSO-*d*_6_) *δ* 8.87 (s, 1H), 8.08 (s, 1H), 8.02 (d, *J* = 8.9 Hz, 1H), 7.92 (d, *J* = 9.3 Hz, 1H), 7.88 (d, *J* = 8.6 Hz, 2H), 7.60 (d, *J* = 8.6 Hz, 2H), 6.65 (s, 1H), 4.22–4.07 (m, 2H), 3.87 (dd, *J* = 19.7, 9.3 Hz, 1H), 3.45–3.30 (m, 1H), 2.57 (dd, *J* = 17.5, 4.7 Hz, 1H), 2.41–2.30 (m, 1H), 1.70 (s, 3H), 1.52–1.33 (m, 4H), 0.85 (t, *J* = 7.2 Hz, 3H), 0.77 (t, *J* = 7.2 Hz, 3H). ^13^C NMR (100 MHz, DMSO-*d*_6_) *δ* 170.3, 167.7, 161.5, 140.8, 138.4, 138.3, 131.6, 130.1, 129.8, 129.5, 121.3, 120.9, 81.6, 75.6, 54.7, 48.5, 30.8, 26.3, 25.7, 23.2, 9.9, 9.5. HRMS (ESI): calcd for C_24_H_28_ClN_4_O_5_, [M-H]^−^ 487.1754, found 487.1759.

#### 3.2.17. General Procedure for the Synthesis of Compounds **9a**–**f**

To a stirred solution of dimethyl oxalate (1.30 g, 11 mmol) and an aryl methyl ketone (10 mmol) in toluene (60 mL) was added a solution of potassium *tert*-butoxide (1.35 g, 12 mmol) in THF (60 mL). The resulting solution was stirred at room temperature overnight. The reaction was quenched with 1 *N* HCl solution and extracted with ethyl acetate. The combined organic layers were dried over Na_2_SO_4_, filtered, and concentrated under reduced pressure to provide the crude products **8a**–**f**.

The crude product was dissolved in AcOH (10 mL) and hydrazine hydrate (534 μL, 10 mmol) was added. The solution was heated to 90 °C for 4 h. The solvent was removed under reduced pressure and the residue was dispersed in EtOAc. The precipitate was collected via filtration and washed by a small amount of EtOAc to afford compounds **9a**–**f** without further purification.

#### 3.2.18. General Procedure for the Synthesis of Compounds **10a**–**f**

The preparation of **10a**–**f** was performed similarly as described for compounds **4a**–**k**.

#### 3.2.19. General Procedure for the Synthesis of Compounds **11a**–**f**

The condensation of **10a**–**f** with oseltamivir phosphate to afford **11a**–**f** was performed in a similar manner as described for compounds **5a**–**k**.

#### 3.2.20. General Procedure for the Synthesis of Compounds **12a**–**f**

Each methyl ester of **11a**–**f** was dissolved in MeOH (10 mL), 1 M NaOH aqueous solution (2 mL) was added. The mixture was stirred at 50 °C overnight. The solvent was removed under reduced pressure and the residue was diluted with 10 mL water and washed with DCM (10 mL × 2). The aqueous phase was acidified by 1 M HCl to precipitate the product which was collected by filtration and dried in vacuo.

#### 3.2.21. (*3R,4R,5S*)-4-acetamido-3-(pentan-3-yloxy)-5-(3-phenyl-1*H*-pyrazole-5-carboxamido) cyclohex-1-ene-1-carboxylic acid (**12a**)

Yield 69%; white solid, mp 234–236 °C. ^1^H NMR (400 MHz, DMSO-*d*_6_) *δ* 8.00 (d, *J* = 8.6 Hz, 2H), 7.80 (d, *J* = 7.5 Hz, 2H), 7.46 (t, *J* = 7.4 Hz, 2H), 7.36 (t, *J* = 7.2 Hz, 1H), 7.08 (s, 1H), 6.64 (s, 1H), 4.18–4.08 (m, 2H), 4.00–3.85 (m, 1H), 3.46–3.30 (m, 1H), 2.67 (d, *J* = 15.7 Hz, 1H), 2.35 (dd, *J* = 16.9, 8.8 Hz, 1H), 1.75 (s, 3H), 1.54–1.34 (m, 4H), 0.86 (t, *J* = 7.2 Hz, 3H), 0.80 (t, *J* = 7.2 Hz, 3H). ^13^C NMR (100 MHz, DMSO-*d*_6_) *δ* 170.4, 168.3, 161.3, 147.4, 145.4, 136.6, 130.7, 129.4, 128.7, 125.7, 102.8, 81.6, 75.3, 53.5, 48.4, 30.7, 26.3, 25.8, 23.2, 9.8, 9.6. HRMS (ESI): calcd for C_24_H_29_N_4_O_5_, [M-H]^−^ 453.2143, found 453.2144.

#### 3.2.22. (*3R,4R,5S*)-4-acetamido-5-(3-(4-chlorophenyl)-1*H*-pyrazole-5-carboxamido)-3-(pentan-3-yloxy) cyclohex-1-ene-1-carboxylic acid (**12b**)

Yield 62%; white solid, mp 266–268 °C. ^1^H NMR (400 MHz, DMSO-*d*_6_) *δ* 13.74 (s, 1H), 8.05 (s, 1H), 7.95 (d, *J* = 8.7 Hz, 1H), 7.81 (d, *J* = 8.1 Hz, 2H), 7.52 (d, *J* = 8.1 Hz, 2H), 7.12 (s, 1H), 6.67 (s, 1H), 4.25–4.06 (m, 2H), 3.94 (dd, *J* = 17.6, 8.7 Hz, 1H), 3.45–3.40 (m, 1H), 2.65 (d, *J* = 14.0 Hz, 1H), 2.38 (dd, *J* = 16.9, 9.2 Hz, 1H), 1.75 (s, 3H), 1.56–1.31 (m, 4H), 0.86 (t, *J* = 7.2 Hz, 3H), 0.79 (t, *J* = 7.1 Hz, 3H). ^13^C NMR (100 MHz, DMSO-*d*_6_) *δ* 170.4, 167.9, 160.8, 137.4, 133.2, 130.0, 129.5, 127.4, 103.1, 81.6, 75.2, 53.6, 48.4, 30.5, 26.3, 25.8, 23.1, 9.8, 9.6, 9.3. HRMS (ESI): calcd for C_24_H_28_ClN_4_O_5_, [M-H]^−^ 487.1754, found 487.1761.

#### 3.2.23. (3*R,4R,5S*)-4-acetamido-5-(3-(4-fluorophenyl)-1*H*-pyrazole-5-carboxamido)-3-(pentan-3-yloxy) cyclohex-1-ene-1-carboxylic acid (**12c**)

Yield 55%; white solid, mp 259–261 °C. ^1^H NMR (400 MHz, DMSO-*d*_6_) *δ* 13.46 (s, 1H), 8.02 (s, 1H), 7.96 (d, *J* = 8.8 Hz, 1H), 7.83 (dd, *J* = 8.3, 5.5 Hz, 2H), 7.30 (t, *J* = 8.8 Hz, 2H), 7.07 (s, 1H), 6.68 (s, 1H), 4.22–4.07 (m, 2H), 3.95 (dd, *J* = 18.2, 8.8 Hz, 1H), 3.45–3.40 (m, 1H), 2.65 (dd, *J* = 17.3, 4.1 Hz, 1H), 2.38 (dd, *J* = 17.5, 9.3 Hz, 1H), 1.75 (s, 3H), 1.54–1.34 (m, 4H), 0.86 (t, *J* = 7.3 Hz, 3H), 0.79 (t, *J* = 7.3 Hz, 3H). ^13^C NMR (100 MHz, DMSO-*d*_6_) *δ* 170.4, 167.8, 162.4 (d, *J* = 243.9 Hz), 160.9, 145.3, 137.6, 129.8, 127.8, 127.7 (d, *J* = 8.1 Hz), 116.4 (d, *J* = 21.6 Hz), 102.8, 81.6, 75.2, 53.5, 48.3, 30.4, 26.3, 25.8, 23.1, 9.8, 9.6. HRMS (ESI): calcd for C_24_H_28_FN_4_O_5_, [M-H]^−^ 471.2049, found 471.2051.

#### 3.2.24. (*3R,4R,5S*)-4-acetamido-3-(pentan-3-yloxy)-5-(3-(4-(trifluoromethyl) phenyl)-1*H*-pyrazole-5-carboxamido) cyclohex-1-ene-1-carboxylic acid (**12d**)

Yield 50%; white solid, mp 270–272 °C. ^1^H NMR (400 MHz, DMSO-*d*_6_) *δ* 13.86 (s, 1H), 8.13 (s, 1H), 8.02 (s, 1H), 8.00 (s, 1H), 7.96 (d, *J* = 8.9 Hz, 1H), 7.81 (d, *J* = 8.1 Hz, 2H), 7.24 (s, 1H), 6.68 (s, 1H), 4.21–4.09 (m, 2H), 3.95 (dd, *J* = 18.0, 8.9 Hz, 1H), 3.45–3.30 (m, 1H), 2.65 (dd, *J* = 17.2, 3.6 Hz, 1H), 2.40 (dd, *J* = 17.3, 9.4 Hz, 1H), 1.74 (s, 3H), 1.52–1.35 (m, 4H), 0.85 (t, *J* = 7.3 Hz, 3H), 0.79 (t, *J* = 7.2 Hz, 3H). ^13^C NMR (100 MHz, DMSO-*d*_6_) *δ* 170.4, 167.8, 160.7, 137.7, 129.8, 128.71, 128.68 (q, *J* = 31.7 Hz), 126.4 (q, *J* = 3.6 Hz), 126.2, 126.0, 123.3, 120.6 (q, *J* = 270.2 Hz), 103.8, 81.6, 75.3, 53.7, 48.4, 30.4, 26.3, 25.8, 23.1, 9.8, 9.5. HRMS (ESI): calcd for C_25_H_28_F_3_N_4_O_5_, [M-H]^−^ 521.2017, found 521.2023.

#### 3.2.25. (*3R,4R,5S*)-4-acetamido-5-(3-(4-methoxyphenyl)-1*H*-pyrazole-5-carboxamido)-3-(pentan-3-yloxy) cyclohex-1-ene-1-carboxylic acid (**12e**)

Yield 59%; white solid, mp 273–275 °C. ^1^H NMR (400 MHz, DMSO-*d*_6_) *δ* 7.96 (d, *J* = 8.6 Hz, 2H), 7.72 (d, *J* = 8.7 Hz, 2H), 7.02 (d, *J* = 8.7 Hz, 2H), 6.97 (s, 1H), 6.68 (s, 1H), 4.10 (dd, *J* = 17.4, 11.7 Hz, 2H), 3.96 (dd, *J* = 18.2, 8.7 Hz, 1H), 3.79 (s, 3H), 3.45–3.42 (m, 1H), 2.66 (dd, *J* = 17.5, 4.4 Hz, 1H), 2.36 (dd, *J* = 17.5, 9.1 Hz, 1H), 1.76 (s, 3H), 1.54–1.35 (m, 4H), 0.86 (t, *J* = 7.3 Hz, 3H), 0.80 (t, *J* = 7.3 Hz, 3H). ^13^C NMR (100 MHz, DMSO-*d*_6_) *δ* 170.4, 167.8, 161.4, 159.8, 145.4, 137.5, 129.9, 127.1, 122.8, 114.8, 102.0, 81.6, 75.2, 55.7, 53.4, 48.3, 30.5, 26.3, 25.8, 23.1, 9.8, 9.6. HRMS (ESI): calcd for C_25_H_31_N_4_O_6_, [M-H]^−^ 483.2249, found 483.2253.

#### 3.2.26. (*3R,4R,5S*)-4-acetamido-3-(pentan-3-yloxy)-5-(3-(thiophen-2-yl)-1*H*-pyrazole-5-carboxamido) cyclohex-1-ene-1-carboxylic acid (**12f**)

Yield 65%; white solid, mp 225–227 °C. ^1^H NMR (400 MHz, DMSO-*d*_6_) *δ* 13.64 (s, 1H), 8.24 (s, 1H), 7.92 (d, *J* = 8.9 Hz, 1H), 7.54 (s, 1H), 7.41 (s, 1H), 7.13 (s, 1H), 6.94 (s, 1H), 6.65 (s, 1H), 4.19–4.05 (m, 2H), 3.95–3.90 (m, 1H), 3.45–3.30 (m, 1H), 2.60 (d, *J* = 14.5 Hz, 1H), 2.46–2.33 (m, 1H), 1.73 (s, 3H), 1.56–1.35 (m, 4H), 0.86 (t, *J* = 7.3 Hz, 3H), 0.79 (t, *J* = 7.2 Hz, 3H). ^13^C NMR (100 MHz, DMSO-*d*_6_) *δ* 170.3, 167.9, 160.0, 137.4, 130.1, 128.4, 126.2, 124.6, 102.6, 81.6, 75.3, 53.9, 48.4, 30.5, 26.3, 25.8, 23.6, 9.9, 9.6. HRMS (ESI): calcd for C_22_H_27_N_4_O_5_S, [M-H]^−^ 459.1708, found 459.1712.

#### 3.2.27. General Procedure for the Synthesis of Compounds **13a**–**c**

A mixture of methyl 3-(4-chlorophenyl)-1H-pyrazole-5-carboxylate (**9b**, 237 mg, 1 mmol), K_2_CO_3_ (276 g, 2 mmol), and alkyl bromide (3 mmol) in acetone (10 mL) was heated to reflux for 12 h. After cooling to room temperature, K_2_CO_3_ was filtered off and the filtrate was concentrated and purified by column chromatography, eluting with hexane/ethyl acetate (6:1) to provide the titled compound.

#### 3.2.28. General Procedure for the Synthesis of Compounds **14a**–**c**

The hydrolysis of **13a**–**c** was performed in a similar manner as described for compounds **4a**–**k**.

#### 3.2.29. General Procedure for the Synthesis of Compounds **15a**–**c**

The condensation of **13a**–**c** with oseltamivir phosphate was performed in a similar manner as described for compounds **5a**–**k**.

#### 3.2.30. General Procedure for the Synthesis of Compounds **16a**–**c**

Each ester of **15a**–**c** was dissolved in MeOH (10 mL), 1 M NaOH aqueous solution (2 mL) was added. The mixture was stirred at 50 °C overnight. The solvent was removed under reduced pressure and the residue was diluted with 10 mL water and washed with DCM (10 mL × 2). The aqueous phase was acidified by 1 M HCl to precipitate the product which was collected by filtration and dried in vacuo.

#### 3.2.31. (*3R,4R,5S*)-4-acetamido-5-(3-(4-chlorophenyl)-1-(cyclopropylmethyl)-1*H*-pyrazole-5-carboxamido)-3-(pentan-3-yloxy) cyclohex-1-ene-1-carboxylic acid (**16a**)

Yield 65%; white solid, mp 269–271 °C. ^1^H NMR (400 MHz, DMSO-*d*_6_) *δ* 8.42 (d, *J* = 8.9 Hz, 1H), 8.02 (d, *J* = 9.0 Hz, 1H), 7.76 (d, *J* = 8.2 Hz, 2H), 7.51 (d, *J* = 8.2 Hz, 2H), 7.14 (s, 1H), 6.56 (s, 1H), 4.37 (ddd, *J* = 45.8, 13.6, 7.2 Hz, 2H), 4.17–4.08 (m, 2H), 3.90–3.80 (m, 1H), 3.45–3.32 (m, 1H), 2.59 (d, *J* = 13.2 Hz, 1H), 2.44–2.33 (m, 1H), 1.73 (s, 3H), 1.54–1.36 (m, 4H), 1.34–1.28 (m, 1H), 0.85 (t, *J* = 7.3 Hz, 3H), 0.78 (t, *J* = 7.2 Hz, 3H), 0.46 (d, *J* = 7.6 Hz, 2H), 0.33–0.40 (m, 2H). ^13^C NMR (100 MHz, DMSO-*d*_6_) *δ* 170.3, 169.3, 159.5, 147.7, 137.4, 135.6, 132.7, 132.0, 129.4, 127.1, 104.9, 81.5, 75.8, 55.2, 54.7, 48.8, 31.1, 26.4, 25.7, 23.2, 12.5, 9.9, 9.5, 3.9, 3.8. HRMS (ESI): calcd for C_28_H_34_ClN_4_O_5_, [M-H]^−^ 541.2223, found 541.2229.

#### 3.2.32. (*3R,4R,5S*)-4-acetamido-5-(3-(4-chlorophenyl)-1-(cyclobutylmethyl)-1*H*-pyrazole-5-carboxamido)-3-(pentan-3-yloxy) cyclohex-1-ene-1-carboxylic acid (**16b**)

Yield 51%; white solid, mp 265–267 °C. ^1^H NMR (400 MHz, DMSO-*d*_6_) *δ* 8.39 (d, *J* = 8.8 Hz, 1H), 8.02 (d, *J* = 8.9 Hz, 1H), 7.75 (d, *J* = 8.0 Hz, 2H), 7.51 (d, *J* = 8.0 Hz, 2H), 7.12 (s, 1H), 6.63 (s, 1H), 4.54 (ddd, *J* = 40.1, 13.1, 7.5 Hz, 2H), 4.20–4.10 (s, 2H), 3.88 (dd, *J* = 19.3, 9.3 Hz, 1H), 3.45–3.30 (m, 1H), 2.82–2.71 (m, 1H), 2.56 (d, *J* = 13.6 Hz, 1H), 2.46–2.34 (m, 1H), 1.96–1.85 (m, 2H), 1.85–1.65 (m, 7H), 1.54–1.33 (m, 4H), 0.85 (t, *J* = 7.0 Hz, 3H), 0.78 (t, *J* = 7.0 Hz, 3H). ^13^C NMR (100 MHz, DMSO-*d*_6_) *δ* 170.2, 168.2, 159.5, 147.6, 137.7, 137.5, 132.7, 132.0, 129.4, 127.0, 104.8, 81.6, 75.6, 55.3, 54.5, 48.6, 36.2, 30.7, 26.3, 25.7, 25.5, 25.4, 23.2, 18.2, 9.9, 9.5. HRMS (ESI): calcd for C_29_H_36_ClN_4_O_5_, [M-H]^−^ 555.2380, found 555.2382.

#### 3.2.33. (*3R,4R,5S*)-4-acetamido-5-(3-(4-chlorophenyl)-1-(cyclohexylmethyl)-1*H*-pyrazole-5-carboxamido)-3-(pentan-3-yloxy) cyclohex-1-ene-1-carboxylic acid (**16c**)

Yield 40%; white solid, mp 232–234 °C. ^1^H NMR (400 MHz, DMSO-*d*_6_) *δ* 8.36 (d, *J* = 8.9 Hz, 1H), 8.02 (d, *J* = 9.1 Hz, 1H), 7.75 (d, *J* = 8.3 Hz, 2H), 7.50 (d, *J* = 8.3 Hz, 2H), 7.15 (s, 1H), 6.64 (s, 1H), 4.37 (ddd, *J* = 51.7, 13.0, 7.3 Hz, 2H), 4.22–4.07 (m, 2H), 3.88 (dd, *J* = 19.5, 9.3 Hz, 1H), 3.45–3.30 (m, 1H), 2.56 (dd, *J* = 17.6, 4.7 Hz, 1H), 2.48–2.37 (m, 1H), 1.85 (s, 1H), 1.73 (s, 3H), 1.61 (d, *J* = 16.6 Hz, 3H), 1.54–1.29 (m, 7H), 1.18–0.89 (m, 6H), 0.85 (t, *J* = 7.2 Hz, 3H), 0.78 (t, *J* = 7.2 Hz, 3H). ^13^C NMR (100 MHz, DMSO-*d*_6_) *δ* 170.1, 168.0, 159.4, 147.5, 138.0, 137.8, 132.7, 132.0, 130.1, 129.4, 127.0, 104.7, 81.6, 75.6, 56.6, 54.5, 48.6, 39.0, 30.6, 30.4, 26.34, 26.31, 25.7, 25.6, 23.2, 9.9, 9.5. HRMS (ESI): calcd for C_31_H_40_ClN_4_O_5_, [M-H]^−^ 583.2693, found 583.2699.

#### 3.2.34. General Procedure for the Synthesis of Compounds **19a**–**e** and **20a**–**d**

To a stirred solution of dimethyl oxalate (1.30 g, 11 mmol) and 1-cyclopropylethan-1-one (991 μL, 10 mmol) in toluene (60 mL) was added a solution of potassium *tert*-butoxide (1.35 g, 12 mmol) in THF (60 mmol). The resulting solution was stirred at room temperature overnight. The reaction was quenched with 1 *N* HCl and extracted with ethyl acetate. The combined organic layers were dried over Na_2_SO_4_, filtered, and concentrated under reduced pressure to provide crude product **18**.

Compound 18 was dissolved in EtOH (15 mL) and aryl hydrazine (12 mmol) was added. The mixture was heated to 85 °C for 4 h. The solvent was removed under reduced pressure and the residue was purified by column chromatography, eluting with a gradient of hexane/ethyl acetate (15:1 to 8:1) to provide the desired product from **19a**–**e** to **20a**–**d** (for **19a**–**e**, the methyl esters were transformed to ethyl esters in EtOH).

#### 3.2.35. General Procedure for the Synthesis of Compounds **21a**–**e** and **22a**–**d**

The hydrolysis of **19a**–**e** and **20a**–**d** was performed in a similar manner as described for compounds **4a**–**k**.

#### 3.2.36. General Procedure for the Synthesis of Compounds **23a**–**e** and **25a**–**d**

The condensation of **21a**–**e** and **22a**–**d** with oseltamivir phosphate was performed in a similar manner as described for compounds **5a**–**k**.

#### 3.2.37. General Procedure for the Synthesis of Compounds **24a**–**e** and **26a**–**d**

Each compound of **23a**–**e** and **25a**–**d** (0.2 mmol) was dissolved in MeOH (10 mL), and 1 M NaOH aqueous solution (2 mL) was added. The mixture was stirred at 50 °C overnight. The solvent was removed in vacuo, the residue was diluted with 10 mL water and washed with DCM (10 mL × 2). The aqueous phase was acidified by 1 M HCl to precipitate the product which was collected by filtration and dried in vacuo.

#### 3.2.38. (*3R,4R,5S*)-4-acetamido-5-(5-cyclopropyl-1-phenyl-1*H*-pyrazole-3-carboxamido)-3-(pentan-3-yloxy) cyclohex-1-ene-1-carboxylic acid (**24a**)

Yield 57%; white solid, mp 273–275 °C. ^1^H NMR (400 MHz, DMSO-*d*_6_) *δ* 7.96 (d, *J* = 8.2 Hz, 1H), 7.90 (d, *J* = 8.5 Hz, 1H), 7.66 (d, *J* = 7.8 Hz, 2H), 7.57 (t, *J* = 7.7 Hz, 2H), 7.48 (t, *J* = 7.2 Hz, 1H), 6.69 (s, 1H), 6.48 (s, 1H), 4.19–4.02 (m, 2H), 3.95 (dd, *J* = 15.9, 8.6 Hz, 1H), 3.45–3.30 (m, 1H), 2.59 (dd, *J* = 17.8, 4.5 Hz, 1H), 2.39 (dd, *J* = 17.8, 7.8 Hz, 1H), 1.83 (td, *J* = 8.2, 4.2 Hz, 1H), 1.78 (d, *J* = 10.4 Hz, 3H), 1.55–1.33 (m, 4H), 1.00–0.90 (m, 2H), 0.85–0.73 (m, 8H). ^13^C NMR (100 MHz, DMSO-*d*_6_) *δ* 170.3, 167.9, 161.5, 148.0, 146.7, 139.6, 136.9, 129.9, 129.6, 128.6, 125.2, 103.7, 81.6, 74.6, 52.5, 47.6, 29.8, 26.2, 25.8, 23.1, 9.8, 9.6, 9.2, 9.0, 7.8. HRMS (ESI): calcd for C_27_H_33_N_4_O_5_, [M-H]^−^ 493.2456, found 493.2461.

#### 3.2.39. (*3R,4R,5S*)-4-acetamido-5-(5-cyclopropyl-1-(o-tolyl)-1*H*-pyrazole-3-carboxamido)-3-(pentan-3-yloxy) cyclohex-1-ene-1-carboxylic acid (**24b**)

Yield 61%; white solid, mp 169–171 °C. ^1^H NMR (400 MHz, DMSO-*d*_6_) *δ* 7.89 (dd, *J* = 8.1, 3.5 Hz, 2H), 7.45 (s, 2H), 7.39 (s, 2H), 6.67 (s, 1H), 6.41 (s, 1H), 4.16–4.04 (m, 2H), 3.92 (dd, *J* = 16.3, 8.5 Hz, 1H), 3.45–3.30 (M, 1H), 2.58 (dd, *J* = 17.7, 4.3 Hz, 1H), 2.35 (dd, *J* = 17.7, 7.8 Hz, 1H), 2.02 (s, 3H), 1.74 (s, 3H), 1.48–1.30 (m, 5H), 0.85–0.80 (m, 2H), 0.80–0.70 (m, 8H). ^13^C NMR (100 MHz, DMSO-*d*_6_) *δ* 170.2, 167.8, 161.6, 148.9, 146.5, 138.4, 136.9, 136.1, 131.4, 130.0, 129.9, 128.1, 127.1, 101.5, 81.5, 74.6, 52.6, 47.6, 29.9, 26.1, 25.7, 23.1, 17.3, 9.8, 9.5, 8.8, 8.6, 7.0. HRMS (ESI): calcd for C_28_H_35_N_4_O_5_, [M-H]^−^ 507.2613, found 507.2617.

#### 3.2.40. (*3R,4R,5S*)-4-acetamido-5-(5-cyclopropyl-1-(m-tolyl)-1*H*-pyrazole-3-carboxamido)-3-(pentan-3-yloxy) cyclohex-1-ene-1-carboxylic acid (**24c**)

Yield 53%; white solid, mp 220–222 °C. ^1^H NMR (400 MHz, DMSO-*d*_6_) *δ* 7.97 (d, *J* = 8.2 Hz, 1H), 7.90 (d, *J* = 8.4 Hz, 1H), 7.45 (s, 3H), 7.30 (s, 1H), 6.68 (s, 1H), 6.46 (s, 1H), 4.16–4.06 (m, 2H), 3.95 (dd, *J* = 15.6, 8.3 Hz, 1H), 3.45–3.40 (m, 1H), 2.58 (dd, *J* = 17.6, 3.7 Hz, 1H), 2.40 (s, 4H), 1.82 (d, *J* = 4.6 Hz, 1H), 1.77 (s, 3H), 1.53–1.33 (m, 4H), 0.95 (d, *J* = 6.9 Hz, 2H), 0.85–0.72 (m, 8H). ^13^C NMR (100 MHz, DMSO-*d*_6_) *δ* 170.3, 167.9, 161.5, 147.9, 146.6, 139.6, 139.3, 136.6, 130.0, 129.4, 129.2, 125.7, 122.2, 103.6, 81.7, 74.6, 52.4, 47.4, 29.7, 26.2, 25.8, 23.1, 21.3, 9.8, 9.6, 9.2, 9.0, 7.9. HRMS (ESI): calcd for C_28_H_35_N_4_O_5_, [M-H]^−^ 507.2613, found 507.2618.

#### 3.2.41. (*3R,4R,5S*)-4-acetamido-5-(1-(4-bromophenyl)-5-cyclopropyl-1*H*-pyrazole-3-carboxamido)-3-(pentan-3-yloxy) cyclohex-1-ene-1-carboxylic acid (**24d**)

Yield 55%; white solid, mp 166–168 °C. ^1^H NMR (400 MHz, DMSO-*d*_6_) *δ* 7.98 (d, *J* = 8.2 Hz, 1H), 7.92 (d, *J* = 8.4 Hz, 1H), 7.77 (d, *J* = 8.4 Hz, 2H), 7.64 (d, *J* = 8.5 Hz, 2H), 6.67 (s, 1H), 6.50 (s, 1H), 4.10 (d, *J* = 5.4 Hz, 2H), 3.98–3.91 (m, 1H), 3.45–3.30 (m, 1H), 2.64–2.54 (m, 1H), 2.38 (dd, *J* = 17.7, 7.8 Hz, 1H), 1.89–1.80 (m, 1H), 1.76 (s, 3H), 1.52–1.33 (m, 4H), 0.96 (d, *J* = 7.8 Hz, 2H), 0.85–0.70 (m, 8H). ^13^C NMR (100 MHz, DMSO-*d*_6_) *δ* 170.4, 168.0, 161.4, 148.1, 147.0, 138.9, 136.7, 132.6, 130.1, 127.0, 121.4, 104.3, 81.6, 74.7, 52.6, 47.7, 29.9, 26.2, 25.8, 23.1, 9.8, 9.6, 9.1, 8.9, 7.8. HRMS (ESI): calcd for C_27_H_32_BrN_4_O_5_, [M-H]^−^ 571.1562, found 571.1562.

#### 3.2.42. (*3R,4R,5S*)-4-acetamido-5-(5-cyclopropyl-1-(4-(trifluoromethyl) phenyl)-1*H*-pyrazole-3-carboxamido)-3-(pentan-3-yloxy) cyclohex-1-ene-1-carboxylic acid (**24e**)

Yield 60%; white solid, mp 220–222 °C. ^1^H NMR (400 MHz, DMSO-*d*_6_) *δ* 8.03 (d, *J* = 8.2 Hz, 1H), 8.00–7. 90 (m, 5H), 6.69 (s, 1H), 6.56 (s, 1H), 4.11 (d, *J* = 5.3 Hz, 2H), 3.96 (dd, *J* = 16.3, 8.5 Hz, 1H), 3.45–3.30 (m, 1H), 2.60 (dd, *J* = 17.6, 4.2 Hz, 1H), 2.40 (dd, *J* = 17.7, 7.9 Hz, 1H), 1.99–1.89 (m, 1H), 1.77 (s, 3H), 1.51–1.34 (m, 4H), 1.00 (d, *J* = 7.6 Hz, 2H), 0.72–0.85 (m, 8H). ^13^C NMR (100 MHz, DMSO-*d*_6_) *δ* 170.4, 167.8, 161.2, 148.3, 147.5, 142.8, 137.1, 129.8, 128.5 (d, *J* = 32.0 Hz), 126.9 (d, *J* = 3.1 Hz), 126.9 (d, *J* = 3.9 Hz), 125.3, 124.4 (q, *J* = 270.6 Hz), 104.9, 81.6, 74.7, 52.7, 47.8, 29.8, 26.2, 25.8, 23.1, 9.8, 9.6, 9.2, 9.0, 8.0. HRMS (ESI): calcd for C_28_H_32_F_3_N_4_O_5_, [M-H]^−^ 561.2330, found 561.2335.

#### 3.2.43. (*3R,4R,5S*)-4-acetamido-5-(3-cyclopropyl-1-phenyl-1*H*-pyrazole-5-carboxamido)-3-(pentan-3-yloxy) cyclohex-1-ene-1-carboxylic acid (**26a**)

Yield 51%; white solid, mp 267–269 °C. ^1^H NMR (400 MHz, DMSO-*d*_6_) *δ* 7.96 (d, *J* = 8.3 Hz, 1H), 7.90 (d, *J* = 8.5 Hz, 1H), 7.66 (d, *J* = 7.8 Hz, 2H), 7.57 (t, *J* = 7.7 Hz, 2H), 7.49 (t, *J* = 7.3 Hz, 1H), 6.67 (s, 1H), 6.48 (s, 1H), 4.16–4.06 (m, 2H), 3.94 (dd, *J* = 15.8, 8.7 Hz, 1H), 3.45–3.30 (m, 1H), 2.59 (dd, *J* = 17.7, 4.7 Hz, 1H), 2.38 (dd, *J* = 17.8, 7.9 Hz, 1H), 1.84 (td, *J* = 8.3, 4.1 Hz, 1H), 1.78 (d, *J* = 11.8 Hz, 3H), 1.43 (qt, *J* = 13.8, 7.1 Hz, 4H), 1.04–0.89 (m, 2H), 0.86–0.71 (m, 8H). ^13^C NMR (100 MHz, DMSO-*d*_6_) *δ* 170.3, 168.0, 161.5, 148.0, 146.7, 139.6, 136.6, 130.1, 129.6, 128.6, 125.2, 103.7, 81.6, 74.7, 52.5, 47.6, 29.8, 26.2, 25.8, 23.2, 9.8, 9.6, 9.2, 9.0, 7.8. HRMS (ESI): calcd for C_27_H_33_N_4_O_5_, [M-H]^−^ 493.2456, found 493.2461.

#### 3.2.44. (*3R,4R,5S*)-4-acetamido-5-(3-cyclopropyl-1-(o-tolyl)-1*H*-pyrazole-5-carboxamido)-3-(pentan-3-yloxy) cyclohex-1-ene-1-carboxylic acid (**26b**)

Yield 45%; white solid, mp 168–170 °C. ^1^H NMR (400 MHz, DMSO-*d*_6_) *δ* 7.88 (d, *J* = 8.1 Hz, 2H), 7.42 (d, *J* = 26.3 Hz, 4H), 6.67 (s, 1H), 6.41 (s, 1H), 4.20–4.10 (m, 2H), 3.92 (dd, *J* = 16.2, 8.3 Hz, 1H), 3.45–3.30 (m, 1H), 2.58 (dd, *J* = 17.6, 3.6 Hz, 1H), 2.36 (dd, *J* = 17.5, 7.6 Hz, 1H), 2.02 (s, 3H), 1.74 (s, 3H), 1.49–1.31 (m, 5H), 0.90–0.85 (m, 2H), 0.80–0.70 (m, 8H). ^13^C NMR (100 MHz, DMSO-*d*_6_) *δ* 170.2, 167.8, 161.6, 148.9, 146.5, 138.4, 137.0, 136.1, 131.4, 130.0, 129.9, 128.1, 127.1, 101.5, 81.5, 74.7, 52.6, 47.6, 29.9, 26.1, 25.7, 23.1, 17.3, 9.8, 9.5, 8.8, 8.6, 7.0. HRMS (ESI): calcd for C_28_H_35_N_4_O_5_, [M-H]^−^ 507.2613, found 507.2614.

#### 3.2.45. (*3R,4R,5S*)-4-acetamido-5-(3-cyclopropyl-1-(m-tolyl)-1*H*-pyrazole-5-carboxamido)-3-(pentan-3-yloxy) cyclohex-1-ene-1-carboxylic acid (**26c**)

Yield 48%; white solid, mp 159–161 °C. ^1^H NMR (400 MHz, DMSO-*d*_6_) *δ* 7.97 (d, *J* = 8.2 Hz, 1H), 7.91 (d, *J* = 8.4 Hz, 1H), 7.45 (s, 3H), 7.29 (s, 1H), 6.69 (s, 1H), 6.46 (s, 1H), 4.17–4.04 (m, 2H), 3.95 (dd, *J* = 15.6, 8.2 Hz, 1H), 3.45–3.30 (m, 1H), 2.58 (dd, *J* = 17.8, 4.0 Hz, 1H), 2.40 (s, 4H), 1.82 (d, *J* = 4.8 Hz, 1H), 1.77 (s, 3H), 1.50–1.35 (m, 4H), 0.95 (d, *J* = 7.0 Hz, 2H), 0.85–0.70 (m, 8H). ^13^C NMR (100 MHz, DMSO-*d*_6_) *δ* 170.3, 167.9, 161.5, 147.9, 146.6, 139.5, 139.3, 136.8, 129.8, 129.4, 129.2, 125.7, 122.1, 103.6, 81.7, 74.6, 52.4, 47.4, 29.7, 26.2, 25.8, 23.1, 21.3, 9.8, 9.6, 9.2, 9.0, 7.9. HRMS (ESI): calcd for C_28_H_35_N_4_O_5_, [M-H]^−^ 507.2613, found 507.2614.

#### 3.2.46. (*3R,4R,5S*)-4-acetamido-5-(1-(4-bromophenyl)-3-cyclopropyl-1*H*-pyrazole-5-carboxamido)-3-(pentan-3-yloxy) cyclohex-1-ene-1-carboxylic acid (**26d**)

Yield 40%; white solid, mp 169–171 °C. ^1^H NMR (400 MHz, DMSO-*d*_6_) *δ* 7.97 (d, *J* = 8.0 Hz, 1H), 7.90 (d, *J* = 8.3 Hz, 1H), 7.77 (d, *J* = 8.3 Hz, 2H), 7.64 (d, *J* = 8.2 Hz, 2H), 6.68 (s, 1H), 6.50 (s, 1H), 4.30–4.05 (m, 2H), 3.94 (dd, *J* = 15.9, 8.2 Hz, 1H), 3.45–3.30 (m, 1H), 2.58 (d, *J* = 13.8 Hz, 1H), 2.39 (dd, *J* = 17.3, 7.5 Hz, 1H), 1.91–1.80 (m, 1H), 1.76 (s, 3H), 1.50–1.35 (m, 4H), 0.96 (d, *J* = 5.9 Hz, 2H), 0.85–0.70 (m, 8H). ^13^C NMR (100 MHz, DMSO-*d*_6_) *δ* 170.3, 167.9, 161.3, 148.1, 147.0, 138.9, 136.9, 132.6, 129.9, 127.0, 121.4, 104.3, 81.6, 74.7, 52.6, 47.7, 29.8, 26.2, 25.8, 23.1, 9.8, 9.6, 9.1, 8.9, 7.8. HRMS (ESI): calcd for C_27_H_32_BrN_4_O_5_, [M-H]^−^ 571.1562, found 571.1565.

### 3.3. In Silico Studies

The structure of NA was extracted from PDB 2HU0. Structures of the compounds and the protein for docking were imported to Maestro 10.2, and the conformations were generated with the panel “Protein Preparation Wizard” and “LigPrep”, respectively. Molecular docking was performed using the Glide in Maestro 10.2, and ADME properties were predicted by Qikprop (Maestro 10.2). The docking and ADME calculation processes were conducted with the default parameters unless otherwise mentioned.

## 4. Conclusions

The 150-cavity of NA represents a potential binding site for novel NA inhibitors. In the present study, we designed and synthesized a series of pyrazole-based C-5-NH_2_-acyl derivatives of **OC**. Most of the derivatives exhibited moderate to high inhibitory activity against NA at the μM level, in which compounds **12c** and **26b** demonstrated the greatest activity. Moreover, in silico ADME prediction suggest that the four compounds have drug-like pharmacokinetic properties. Especially, parameters for oral absorption and cell permeability indicated the prospect of direct oral administration of the derivatives, such as **6i**, **12c, 24b**, and **26b**. Further molecular docking studies revealed that the derivatives could dock into the active site and the 150-cavity as expected. However, calculated binding energy showed that these derivatives possessed higher binding energy than **OC** (Table S1), indicating these modifications would reduce the binding affinity to NA. Considering the inhibitory activity of compounds **12c** and **26b**, as well as their docking models, it is expected that further optimized NA inhibitors could be identified based on the work described in this study.

C-5-NH_2_ modification of oseltamivir represents one of the most attractive research interests in the development of novel NA 150-cavity binders. Previously, we initiated the strategy of using heterocycles to link oseltamivir and the 150-binding fragments [17]. Inspired by the fact that pyrazole is one of the most widely used heterocycles in drug discovery, we validated our hypothesis that modifying the oseltamivir C-5-NH_2_ group by incorporating the pyrazole moiety has the potential to develop novel NA inhibitors. This work together with our previous discovery on the development of NA inhibitors containing other heterocycles will pave a solid basis for the design of novel 150-binders as NA inhibitors in the future.

## Figures and Tables

**Table 1:** Inhibitory rates of designed compounds against A/H3N2 influenza NA ^a^.

Derivatives	Cpd.	R	10 μM	Cpd.	R	10 μM
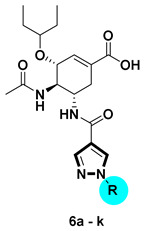	**OC**	\	97.46%	**6f**	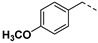	26.79%
	**6a**	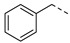	32.02%	**6g**	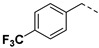	21.72%
	**6b**	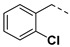	18.88%	**6h**	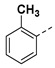	38.37%
	**6c**	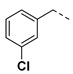	23.88%	**6i**	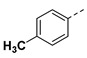	52.31%
	**6d**	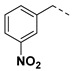	12.26%	**6j**	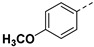	34.15%
	**6e**	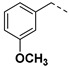	23.37%	**6k**	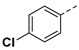	25.67%
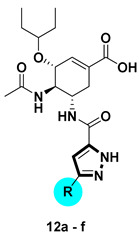	**12a**	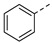	42.81%	**12d**	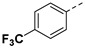	46.7%
	**12b**	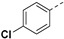	50.43%	**12e**	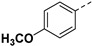	42.02%
	**12c**	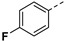	56.45%	**12f**	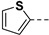	40.02%
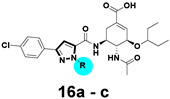	**16a**	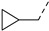	31.50%	**16c**	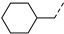	48.50%
	**16b**	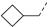	39.81%			
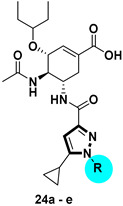	**24a**	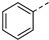	30.57%	**24d**	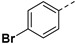	42.68%
	**24b**	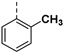	60.91%	**24e**	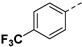	44.47%
	**24c**	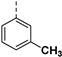	34.75%			
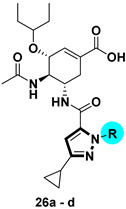	**26a**	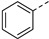	30.41%	**26c**	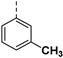	31.53%
	**26b**	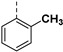	72.80%	**26d**	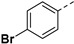	37.17%

^a^$\left(\%\right)\mathrm{Inhibitory}\;\mathrm{rate}\;=\frac{\left(A_{x}-A_{bg}\right)-\left(A_{0}-A_{bg}\right)}{A_{0}-A_{bg}}\times 100\%$, *A_o_* is the absorbance of the control (no drug control), *A_bg_* is the absorbance of the background, *A_x_* is the absorbance of the sample or control compound (**OC**).

**Table 2:** Inhibitory activity of **6i**, **12c**, **24b**, and **26b** against clinically isolated A/H3N2 NA (μM) ^a^.

No.	OC ^b^	6i	12c	24b	26b
IC_50_ (μM) ^a^	1.04 ± 0.01	27.56 ± 0.55	6.98 ± 0.08	26.99 ± 0.31	12.27 ± 0.14

^a^ IC_50_ is the compound concentration producing 50% inhibition of NA, the values are the mean of three independent experiments. ^b^ IC_50_ unit of OC is nM.

**Table 3:** Comparison of computational pharmacokinetic parameters (ADME) of **OC** derivatives.

Principal Descriptors	OC	6i	12c	24b	26b	Standard Range *
**QPlogPo/w**	−1.654	3.361	3.337	4.106	4.380	−2.0–6.5
**QPlogS**	−1.164	−5.741	−5.931	−6.323	−6.648	−6.5–0.5
**QPlogKhsa**	−0.873	−0.043	−0.036	0.277	0.291	−1.5–1.5
**QPlogBB**	−1.05	−2.186	−2.186	−2.086	−1.714	−3.0–1.2
**#metab**	3	3	2	3	3	1–8
**CNS**	−2	−2	−2	−2	−2	−2 (inactivity), +2 (activity)
**QPloghERG**	−1.322	−3.348	−2.975	−3.006	−3.085	<−5
**QPPCaco**	7.34	20.787	17.378	22.727	54.32	<25 poor, >500 great
**QPPMDCK**	6.146	17.413	19.348	20.759	42.816	<25 poor, >500 great
**(%) Human Oral** **Absorption**	32.756	70.213	68.675	62.305	70.687	<25% is poor, >80% is high
**Human Oral** **Absorption**	Middle	Middle	Middle	Middle	Middle	

* Statistics of 95% of known drugs according to Qikprop (Maestro 10.2).

**Table 4:** Hydrogen bonding interactions between NA (PDB: 2HU0) and compounds **12c** and **26b**.

H-Bonding between NA and 12c		H-Bonding between NA and 26b	
Hydrogen Bonds	Distance (Å)	Hydrogen Bonds	Distance (Å)
SER246: HG/**12c**: O9	2.10	ARG156: HH11/**26b**: O19	1.82
ASN294: HD21/**12c**: O8	3.15	ARG156: HH21/**26b**: O19	2.67
ARG371: HH11/**12c**: O23	2.24	ARG292: HH21/**26b**: O9	1.92
ARG371: HH21/**12c**: O23	1.95	ARG371: HH11/**26b**: O9	1.57
ARG371: HH21/**12c**: N27	2.67	ARG371: HH21/**26b**: O9	3.01
ARG430: HH22/**12c**: F34	2.93	**26b**: H59/ASP151: OD1	2.71

## Data Availability

Data are provided within the article and the Supplementary Materials.

## Supplementary Materials

The following are available online at https://www.mdpi.com/article/10.3390/ph14040371/s1, docking models of the products, binding free energy of representative products, experimental data of the intermediates; ^1^H and ^13^C NMR Spectra of the products.

## Abbreviations

OC	oseltamivir carboxylate
OP	oseltamivir phosphate
HA	Hemagglutinin
NA	Neuraminidase
NAIs	Neuraminidase inhibitors
ADME	absorption, distribution, metabolism and excretion
DCM	Dichloromethane
DIPEA	*N,N*-Diisopropylethylamine
HATU	2-(7-Azabenzotriazol-1-yl)-*N,N,N′,N′*-tetramethyluronium hexafluorophosphate

## References

[1] Word Health Organization Report. https://www.who.int/news/item/13-12-2017-up-to-650-000-people-die-of-respiratory-diseases-linked-to-seasonal-flu-each-year.

[2] Cox N.J., Bender C.A. (1995). The molecular epidemiology of influenza viruses. Semin. Virol..

[3] Bouvier N.M., Palese P. (2008). The biology of influenza viruses. Vaccine.

[4] Zhuang Q., Wang S., Liu S., Hou G., Li J., Jiang W., Wang K., Peng C., Liu D., Guo A. (2019). Diversity and distribution of type A influenza viruses: An updated panorama analysis based on protein sequences. Virol. J..

[5] Kilbourne E.D. (2006). Influenza pandemics of the 20th century. Emerg. Infect. Dis..

[6] Dawood F.S., Iuliano A.D., Reed C., Meltzer M.I., Shay D.K., Cheng P.-Y., Bandaranayake D., Breiman R.F., Brooks W.A., Buchy P. (2012). Estimated global mortality associated with the first 12 months of 2009 pandemic influenza A H1N1 virus circulation: A modelling study. Lancet Infect. Dis..

[7] Jefferson T., Demicheli V., Rivetti D., Jones M., Di Pietrantonj C., Rivetti A. (2006). Antivirals for influenza in healthy adults: Systematic review. Lancet.

[8] Noshi T., Kitano M., Taniguchi K., Yamamoto A., Omoto S., Baba K., Hashimoto T., Ishida K., Kushima Y., Hattori K. (2018). In vitro characterization of baloxavir acid, a first-in-class cap-dependent endonuclease inhibitor of the influenza virus polymerase PA subunit. Antivir. Res..

[9] Gubareva L.V., Fry A.M. (2020). Baloxavir and Treatment-Emergent Resistance: Public Health Insights and Next Steps. J. Infect. Dis..

[10] Greener M. (2009). Using oseltamivir (Tamiflu) as a first line treatment for seasonal or pandemic flu. Nurs. Times.

[11] Gamblin S.J., Skehel J.J. (2010). Influenza hemagglutinin and neuraminidase membrane glycoproteins. J. Biol. Chem..

[12] Russell R.J., Haire L.F., Stevens D.J., Collins P.J., Lin Y.P., Blackburn G.M., Hay A.J., Gamblin S.J., Skehel J.J. (2006). The structure of H5N1 avian influenza neuraminidase suggests new opportunities for drug design. Nature.

[13] Wu Y., Qin G., Gao F., Liu Y., Vavricka C.J., Qi J., Jiang H., Yu K., Gao G.F. (2013). Induced opening of influenza virus neuraminidase N2 150-loop suggests an important role in inhibitor binding. Sci. Rep..

[14] Xie Y., Xu D., Huang B., Ma X., Qi W., Shi F., Liu X., Zhang Y., Xu W. (2014). Discovery of N-substituted oseltamivir derivatives as potent and selective inhibitors of H5N1 influenza neuraminidase. J. Med. Chem..

[15] Zhang J., Poongavanam V., Kang D., Bertagnin C., Lu H., Kong X., Ju H., Lu X., Gao P., Tian Y. (2018). Optimization of N-Substituted Oseltamivir Derivatives as Potent Inhibitors of Group-1 and -2 Influenza A Neuraminidases, Including a Drug-Resistant Variant. J. Med. Chem..

[16] Zhang J., Murugan N.A., Tian Y., Bertagnin C., Fang Z., Kang D., Kong X., Jia H., Sun Z., Jia R. (2018). Structure-Based Optimization of N-Substituted Oseltamivir Derivatives as Potent Anti-Influenza A Virus Agents with Significantly Improved Potency against Oseltamivir-Resistant N1-H274Y Variant. J. Med. Chem..

[17] Ye J., Yang X., Xu M., Chan P.K., Ma C. (2019). Novel N-Substituted oseltamivir derivatives as potent influenza neuraminidase inhibitors: Design, synthesis, biological evaluation, ADME prediction and molecular docking studies. Eur. J. Med. Chem..

[18] Wang P., Oladejo B.O., Li C., Fu L., Zhang S., Qi J., Lv X., Li X. (2021). Structure-based design of 5′-substituted 1,2,3-triazolylated oseltamivir derivatives as potent influenza neuraminidase inhibitors. RSC Adv..

[19] Wang K., Lei Z., Zhao L., Chen B., Yang F., Liu K., Zhu H., Zhao H., Cao R., Zhang K. (2020). Design, synthesis and biological evaluation of oseltamivir derivatives containing pyridyl group as potent inhibitors of neuraminidase for influenza A. Eur. J. Med. Chem..

[20] Wang K., Yang F., Wang L., Liu K., Sun L., Lin B., Hu Y., Wang B., Cheng M., Tian Y. (2017). Synthesis and biological evaluation of NH2-acyl oseltamivir analogues as potent neuraminidase inhibitors. Eur. J. Med. Chem..

[21] Antilla J.C., Baskin J.M., Barder T.E., Buchwald S.L. (2004). Copper-diamine-catalyzed N-arylation of pyrroles, pyrazoles, indazoles, imidazoles, and triazoles. J. Org. Chem..

[22] Dawood K.M., Abdel-Gawad H., Mohamed H.A., Badria F.A. (2010). Synthesis, anti-HSV-1, and cytotoxic activities of some new pyrazole- and isoxazole-based heterocycles. Med. Chem. Res..

[23] Levent S., Caliskan B., Ciftci M., Ozkan Y., Yenicesu I., Unver H., Banoglu E. (2013). Pyrazole derivatives as inhibitors of arachidonic acid-induced platelet aggregation. Eur. J. Med. Chem..

[24] Aghazadeh Tabrizi M., Baraldi P.G., Baraldi S., Ruggiero E., De Stefano L., Rizzolio F., Di Cesare Mannelli L., Ghelardini C., Chicca A., Lapillo M. (2018). Discovery of 1,5-Diphenylpyrazole-3-Carboxamide Derivatives as Potent, Reversible, and Selective Monoacylglycerol Lipase (MAGL) Inhibitors. J. Med. Chem..

