# Supplementary material for: Design, Synthesis, Biological Evaluation and In Silico Studies of Pyrazole-Based NH2-Acyl Oseltamivir Analogues as Potent Neuraminidase Inhibitors
Source: Pharmaceuticals (Basel). 2021 Apr 16;14(4):371. doi: 10.3390/ph14040371 (PMC8073777; doi:10.3390/ph14040371)

Design, synthesis, biological evaluation and *in silico* studies of pyrazole-based *NH2*-acyl oseltamivir analogues as potent neuraminidase inhibitors

Jiqing Ye [a], Lin Lin [b], Jinyi Xu [c], Paul Kay-sheung Chan [b, d], Xiao Yang [b,*] and Cong Ma [a,*]

[a] *State Key Laboratory of Chemical Biology and Drug Discovery, Department of Applied Biology and Chemical Technology, The Hong Kong Polytechnic University, Kowloon, Hong Kong Special Administrative Region, China*

[b] *Department of Microbiology, The Chinese University of Hong Kong, Prince of Wales Hospital, Shatin, Hong Kong Special Administrative Region, China*

[c] *State Key Laboratory of Natural Medicines and Department of Medicinal Chemistry, China Pharmaceutical University, 24 Tong Jia Xiang, Nanjing, 210009, China*

[d] *Stanley Ho Centre for Emerging Infectious Diseases, The Chinese University of Hong Kong, Shatin, Hong Kong Special Administrative Region, China*

Table of content

1. **Figure S1**. Docking models of all the final products to NA (PDB: 2HU0), and detailed interactions of **6i**, **16c**, **24b** with NA……………………………**S2 – S4**

2. **Table S1.** Calculated binding energy of compounds **6i**, **12c**, **24b** and **26b** in complex with NA (PDB: 2HU0) ………………………………………………**S5**

3. Experimental data for the intermediates ………………………………**S6 – S14**

4. ^{1}H and ^{13}C NMR Spectra of the products ………………………………**S15 – S43**

* Corresponding authors: cong.ma@polyu.edu.hk (C. Ma); xiaoyang@cuhk.edu.hk (X. Yang)

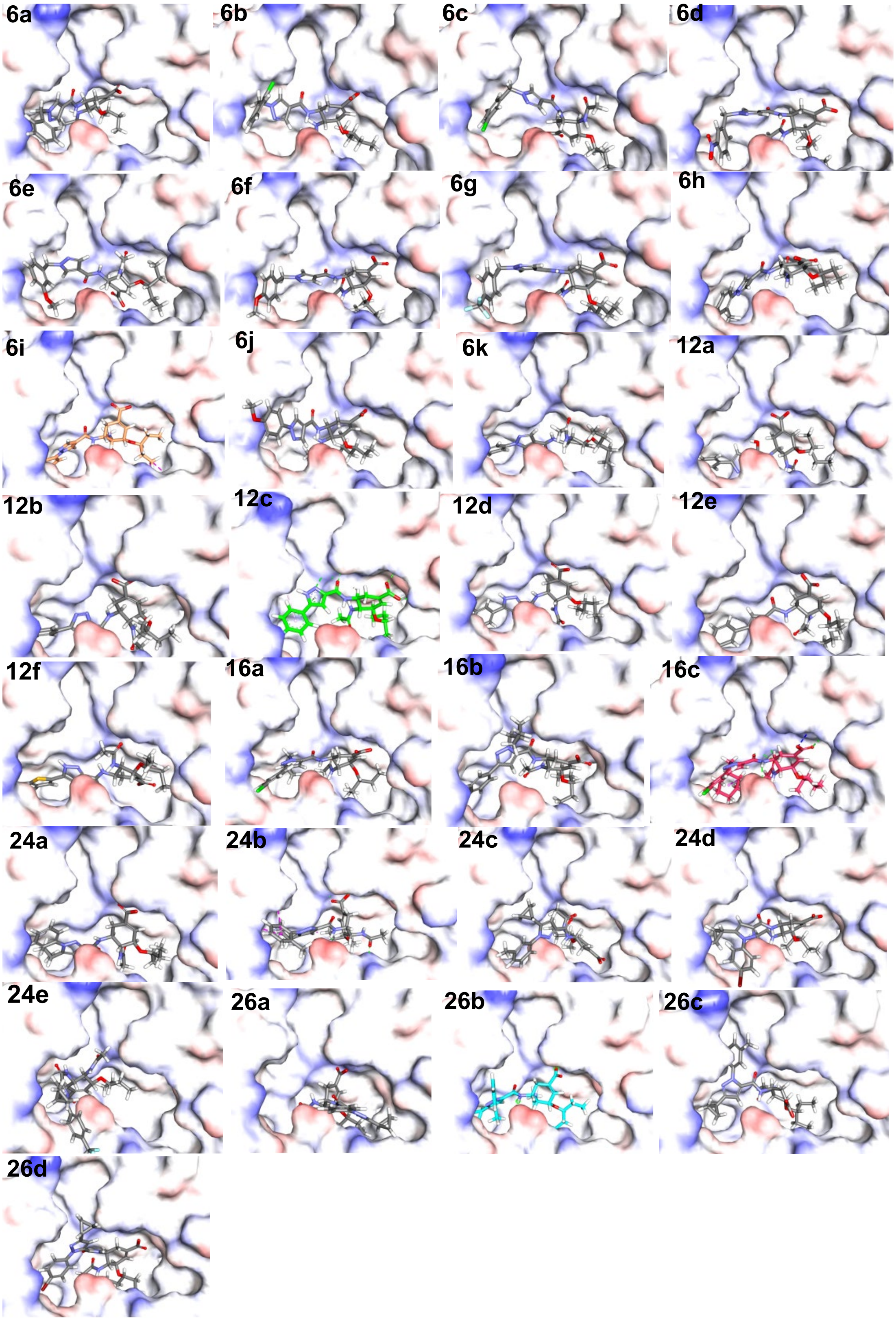
6a
6b
6c
6d
6e
6f
6g
6h
6i
6j
6k
12a
12b
12c
12d
12e
12f
16a
16b
16c
24a
24b
24c
24d
24e
26a
26b
26c
26d

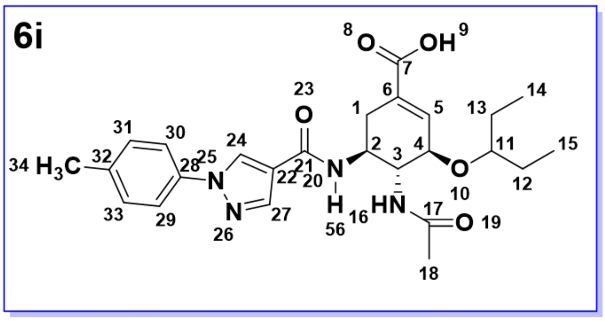

H-bond	Distance (Å)
ARG156:HH11/**6i**:O19	1.80
ARG156:HH21 /**6i**:O19	2.45
ARG292:HH11 /**6i**:O9	1.66
ARG292:HH21/**6i**:O9	2.17
ARG371:HH21/**6i**:O8	1.80
**6i**:H56/ASP151:OD1	2.22

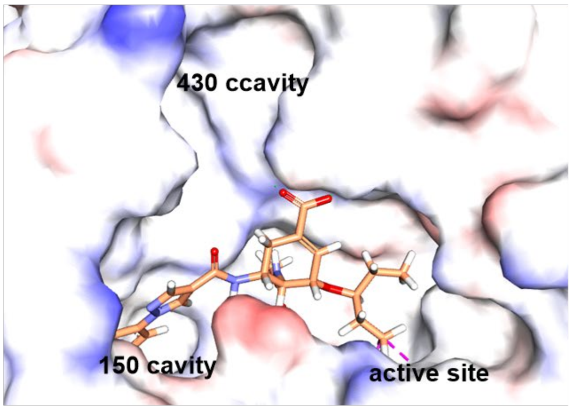

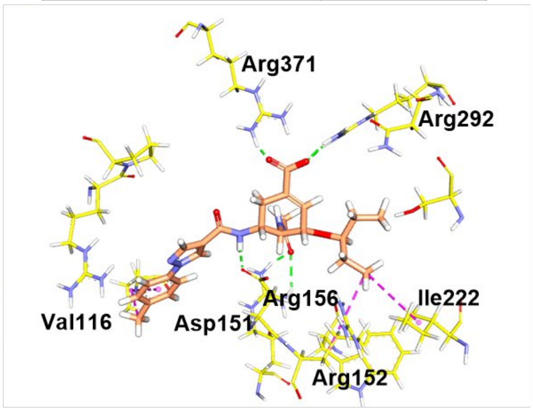

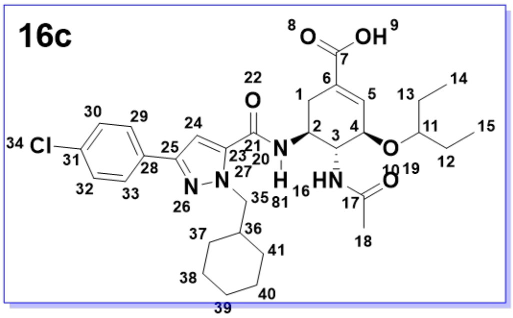

H-bond	Distance (Å)
ARG118:HH12/16c:O19	2.61
ARG118:HH22/16c:O19	2.46
ARG118:HH22/16c:O22	1.96
ASN294:HD22/16c:O8	2.90
16c:H81/ASP151:OD2	2.81

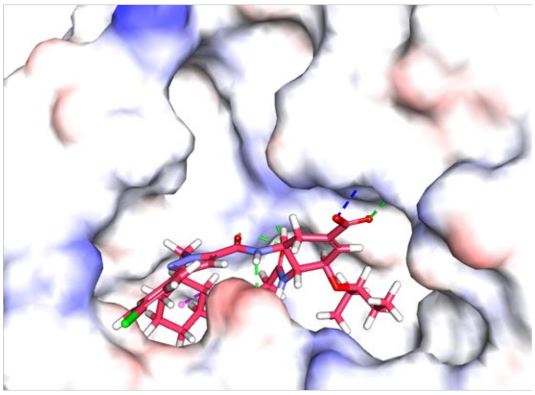

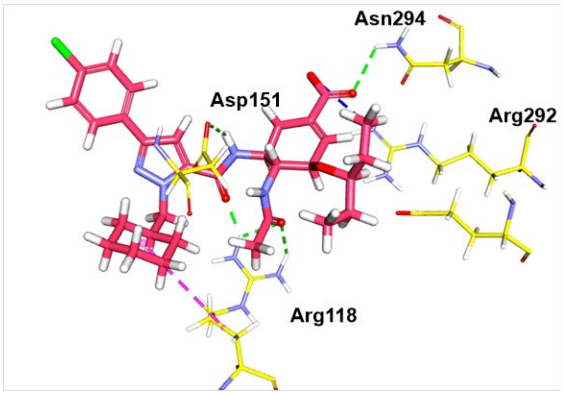

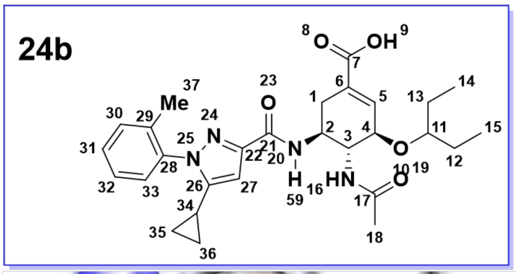

H-bond	Distance (Å)
ARG152:HH21/**24b**:O19	2.03
ARG292:HH11/**24b**:O9	1.76
ARG292:HH21/**24b**:O9	2.36
ARG371:HH11/**24b**:O9	1.78
ARG371:HH21/**24b**:O8	1.80
**24b**:H59/ASP151:OD2	2.09

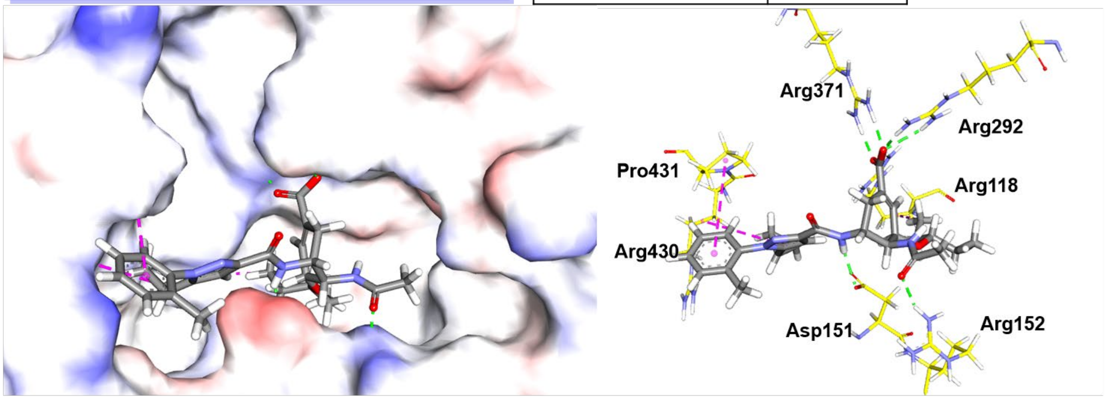

Figure S1. Binding positions of all final compounds to NA (PDB: 2HU0), and detailed interactions of **6i**, **16c**, **24b** with NA: Hydrogen/ionic bonding interactions (green) and hydrophobic interactions (magenta), hydrogen bonds were summarized in the tables.

The binding free energy of the ligand-protein complexes was calculated using Discovery studio [1]. Results showed that all the derivatives possessed higher binding energy than **OC**, indicating the binding affinities of the derivatives were lower than **OC**. This might because the modifications may disturb the key interactions between OC and NA, resulting in reduced inhibitory activities.

Table S1. Calculated binding energy of compounds **6i**, **12c**, **24b** and **26b** in complex with NA (PDB: 2HU0)

**No.**	**OC**	**6i**	**12c**	**24b**	**26b**
*$\Delta E_{binding}$ (Kcal/mol)	-237.295	-75.969	-20.229	-28.763	-53.392

*$\Delta E_{binding} = \Delta E_{Complex} - \Delta E_{Receptor} - \Delta E_{Ligand}$.

Reference

1. Raharjo, S. J., Mahdi, C., Nurdiana, N., Kikuchi, T., & Fatchiyah, F. Binding energy calculation of patchouli alcohol isomer cyclooxygenase complexes suggested as COX-1/COX-2 selective inhibitor. *Advances in bioinformatics*, 2014 (2014).

3. Experimental data for the intermediates

*Ethyl 1-benzyl-1H-pyrazole-4-carboxylate (**3a**)*

Yield 67%; white solid. ^{1}H NMR (400 MHz, $CDCl_3$) δ 7.94 (s, 1H), 7.85 (s, 1H), 7.40 – 7.31 (m, 3H), 7.28 – 7.21 (m, 2H), 5.31 (s, 2H), 4.27 (q, *J* = 7.1 Hz, 2H), 1.32 (t, *J* = 7.1 Hz, 3H).

*Ethyl 1-(2-chlorobenzyl)-1H-pyrazole-4-carboxylate (**3b**)*

Yield 61%; white solid. ^{1}H NMR (400 MHz, $CDCl_3$) δ 7.95 (s, 1H), 7.92 (s, 1H), 7.48 – 7.37 (m, 1H), 7.32 – 7.23 (m, 2H), 7.18 – 7.06 (m, 1H), 5.43 (s, 2H), 4.28 (q, *J* = 7.1 Hz, 2H), 1.33 (t, *J* = 7.1 Hz, 3H).

*Ethyl 1-(3-chlorobenzyl)-1H-pyrazole-4-carboxylate (**3c**)*

Yield 95%; white solid. ^{1}H NMR (400 MHz, $CDCl_3$) δ 7.95 (s, 1H), 7.89 (s, 1H), 7.34 – 7.27 (m, 2H), 7.22 (s, 1H), 7.12 (d, *J* = 6.3 Hz, 1H), 5.28 (s, 2H), 4.29 (q, *J* = 7.1 Hz, 2H), 1.34 (t, *J* = 7.1 Hz, 3H).

*Ethyl 1-(3-nitrobenzyl)-1H-pyrazole-4-carboxylate (**3d**)*

Yield 91%; white solid. ^{1}H NMR (400 MHz, $CDCl_3$) δ 8.23 – 8.17 (m, 1H), 8.11 (s, 1H), 7.97 (s, 2H), 7.56 (d, *J* = 5.2 Hz, 2H), 5.42 (s, 2H), 4.30 (q, *J* = 7.1 Hz, 2H), 1.34 (t, *J* = 7.1 Hz, 3H).

*Ethyl 1-(3-methoxybenzyl)-1H-pyrazole-4-carboxylate (**3e**)*

Yield 87%; white solid. ^{1}H NMR (400 MHz, $CDCl_3$) δ 7.94 (s, 1H), 7.86 (s, 1H), 7.29 (d, *J* = 7.9 Hz, 1H), 6.87 (dd, *J* = 8.3, 2.3 Hz, 1H), 6.83 (d, *J* = 7.6 Hz, 1H), 6.77 (s, 1H), 5.27 (s, 2H), 4.27 (q, *J* = 7.2 Hz, 2H), 1.33 (t, *J* = 7.1 Hz, 3H).

*Ethyl 1-(4-methoxybenzyl)-1H-pyrazole-4-carboxylate (**3f**)*

Yield 89%; white solid. ^{1}H NMR (400 MHz, $CDCl_3$) δ 7.92 (s, 1H), 7.80 (s, 1H), 7.21 (d, *J* = 8.6 Hz, 2H), 6.92 – 6.86 (m, 2H), 5.23 (s, 2H), 4.27 (q, *J* = 7.2 Hz, 2H), 3.81 (s, 3H), 1.32 (t, *J* = 7.1 Hz, 3H).

*Ethyl 1-(4-(trifluoromethyl)benzyl)-1H-pyrazole-4-carboxylate (**3g**)*

Yield 87%; white solid. ^{1}H NMR (400 MHz, $CDCl_3$) δ 7.96 (s, 1H), 7.91 (s, 1H), 7.62 (d, *J* = 8.1 Hz, 2H), 7.34 (d, *J* = 8.0 Hz, 2H), 5.37 (s, 2H), 4.29 (q, *J* = 7.1 Hz, 2H), 1.34 (t, *J* = 7.1 Hz, 3H).

*Ethyl 1-(o-tolyl)-1H-pyrazole-4-carboxylate (**3h**)*

Yield 34%; colorless oil. ^{1}H NMR (400 MHz, $CDCl_3$) δ 8.11 (s, 1H), 8.09 (s, 1H), 7.41 – 7.27 (m, 4H), 4.34 (q, *J* = 7.1 Hz, 2H), 2.25 (s, 3H), 1.37 (t, *J* = 7.1 Hz, 3H).

*Ethyl 1-(p-tolyl)-1H-pyrazole-4-carboxylate (**3i**)*

Yield 52%; white solid. ^{1}H NMR (400 MHz, $CDCl_3$) δ 8.36 (s, 1H), 8.08 (s, 1H), 7.58 (d, *J* = 8.5 Hz, 2H), 7.27 (d, *J* = 10.0 Hz, 2H), 4.34 (q, *J* = 7.1 Hz, 2H), 2.40 (s, 3H), 1.38 (t, *J* = 7.1 Hz, 3H).

*Ethyl 1-(4-methoxyphenyl)-1H-pyrazole-4-carboxylate (**3j**)*

Yield 55%; white solid. ^{1}H NMR (400 MHz, $CDCl_3$) δ 8.30 (s, 1H), 8.07 (s, 1H), 7.64 – 7.56 (m, 2H), 7.04 – 6.94 (m, 2H), 4.34 (q, *J* = 7.1 Hz, 2H), 3.86 (s, 3H), 1.38 (t, *J* = 7.1 Hz, 3H).

*Ethyl 1-(4-chlorophenyl)-1H-pyrazole-4-carboxylate (**3k**)*

Yield 42%; white solid. ^{1}H NMR (400 MHz, $CDCl_3$) δ 8.38 (s, 1H), 8.09 (s, 1H), 7.66 (d, *J* = 8.8 Hz, 2H), 7.46 (d, *J* = 8.8 Hz, 2H), 4.34 (q, *J* = 7.1 Hz, 2H), 1.38 (t, *J* = 7.1 Hz, 3H).

*Ethyl (3R,4R,5S)-4-acetamido-5-(1-benzyl-1H-pyrazole-4-carboxamido)-3-(pentan-3-yloxy)cyclohex-1-ene-1-carboxylate (**5a**)*

Yield 49%; white solid. ^{1}H NMR (400 MHz, $CDCl_3$) δ 7.83 (s, 1H), 7.79 (s, 1H), 7.38 – 7.28 (m, 3H), 7.25 – 7.20 (m, 2H), 7.17 (d, *J* = 8.2 Hz, 1H), 6.80 (s, 1H), 6.28 (d, *J* = 7.2 Hz, 1H), 5.26 (s, 2H), 4.27 – 4.15 (m, 3H), 4.12 (t, *J* = 7.0 Hz, 2H), 3.40 (p, *J* = 5.6 Hz, 1H), 2.84 (dd, *J* = 17.9, 5.0 Hz, 1H), 2.46 – 2.33 (m, 1H), 1.85 (s, 3H), 1.50 (ddd, *J* = 10.3, 8.1, 2.7 Hz, 4H), 1.28 (t, *J* = 7.1 Hz, 3H), 0.89 (dd, *J* = 16.7, 7.5 Hz, 6H).

*Ethyl (3R,4R,5S)-4-acetamido-5-(1-(2-chlorobenzyl)-1H-pyrazole-4-carboxamido)-3-(pentan-3-yloxy)cyclohex-1-ene-1-carboxylate (**5b**)*

Yield 90%; white solid. ^{1}H NMR (400 MHz, $CDCl_3$) δ 7.85 (s, 1H), 7.83 (s, 1H), 7.40 (dd, *J* = 7.7, 1.4 Hz, 1H), 7.31 – 7.27 (m, 1H), 7.26 – 7.21 (m, 1H), 7.18 (d, *J* = 7.6 Hz, 1H), 7.10 (dd, *J* = 7.4, 1.7 Hz, 1H), 6.81 (s, 1H), 6.14 (s, 1H), 5.40 (s, 2H), 4.29 – 4.17 (m, 3H), 4.13 (t, *J* = 6.9 Hz, 2H), 3.41 (t, *J* = 5.7 Hz, 1H), 2.87 (dd, *J* = 18.1, 5.0 Hz, 1H), 2.39 (dd, *J* = 17.9, 9.0 Hz, 1H), 1.90 (s, 3H), 1.61 – 1.48 (m, 4H), 1.28 (t, *J* = 7.1 Hz, 3H), 0.90 (q, *J* = 7.4 Hz, 6H).

*Ethyl (3R,4R,5S)-4-acetamido-5-(1-(3-chlorobenzyl)-1H-pyrazole-4-carboxamido)-3-(pentan-3-yloxy)cyclohex-1-ene-1-carboxylate (**5c**)*

Yield 73%; white solid. ^{1}H NMR (400 MHz, $CDCl_3$) δ 7.82 (s, 2H), 7.33 – 7.27 (m, 2H), 7.19 (s, 1H), 7.13 (dd, *J* = 19.2, 6.9 Hz, 2H), 6.82 (s, 1H), 5.94 (s, 1H), 5.25 (s, 2H), 4.28 – 4.16 (m, 3H), 4.16 – 4.06 (m, 2H), 3.41 (p, *J* = 5.6 Hz, 1H), 2.88 (dd, *J* = 18.0, 4.9 Hz, 1H), 2.39 (dd, *J* = 18.0, 9.0 Hz, 1H), 1.91 (s, 3H), 1.61 – 1.48 (m, 4H), 1.28 (t, *J* = 7.1 Hz, 3H), 0.90 (q, *J* = 7.2 Hz, 6H).

*Ethyl (3R,4R,5S)-4-acetamido-5-(1-(3-nitrobenzyl)-1H-pyrazole-4-carboxamido)-3-(pentan-3-yloxy)cyclohex-1-ene-1-carboxylate (**5d**)*

Yield 80%; white solid. ^{1}H NMR (400 MHz, $CDCl_3$) δ 8.24 – 8.14 (m, 1H), 8.07 (s, 1H), 7.91 (s, 1H), 7.86 (s, 1H), 7.55 (d, *J* = 5.3 Hz, 2H), 7.23 (d, *J* = 7.8 Hz, 1H), 6.82 (s, 1H), 5.93 (d, *J* = 7.4 Hz, 1H), 5.40 (s, 2H), 4.28 – 4.16 (m, 3H), 4.16 – 4.02 (m, 2H), 3.41 (p, *J* = 5.6 Hz, 1H), 2.90 (dd, *J* = 18.0, 5.1 Hz, 1H), 2.39 (dd, *J* = 18.0, 9.1 Hz, 1H), 1.93 (s, 3H), 1.61 – 1.49 (m, 4H), 1.29 (t, *J* = 7.1 Hz, 3H), 0.91 (q, *J* = 7.2 Hz, 6H).

*Ethyl (3R,4R,5S)-4-acetamido-5-(1-(3-methoxybenzyl)-1H-pyrazole-4-carboxamido)-3-(pentan-3-yloxy)cyclohex-1-ene-1-carboxylate (**5e**)*

Yield 83%; white solid. ^{1}H NMR (400 MHz, $CDCl_3$) δ 7.81 (s, 1H), 7.79 (s, 1H), 7.27 – 7.22 (m, 1H), 7.14 (d, *J* = 7.5 Hz, 1H), 6.85 (dd, *J* = 8.3, 2.1 Hz, 1H), 6.83 – 6.71 (m, 3H), 6.13 (s, 1H), 5.23 (s, 2H), 4.28 – 4.15 (m, 3H), 4.12 (t, *J* = 6.9 Hz, 2H), 3.77 (s, 3H), 3.47 – 3.36 (m, 1H), 2.86 (dd, *J* = 18.0, 4.8 Hz, 1H), 2.38 (dd, *J* = 17.9, 9.0 Hz, 1H), 1.88 (s, 3H), 1.57 – 1.47 (m, 4H), 1.28 (t, *J* = 7.1 Hz, 3H), 0.90 (q, *J* = 7.6 Hz, 6H).

*Ethyl (3R,4R,5S)-4-acetamido-5-(1-(4-methoxybenzyl)-1H-pyrazole-4-carboxamido)-3-(pentan-3-yloxy)cyclohex-1-ene-1-carboxylate (**5f**)*

Yield 86%; white solid. ^{1}H NMR (400 MHz, $CDCl_3$) δ 7.79 (s, 1H), 7.74 (s, 1H), 7.20 (d, *J* = 8.4 Hz, 2H), 7.12 – 7.00 (m, 1H), 6.87 (d, *J* = 8.5 Hz, 2H), 6.81 (s, 1H), 6.14 – 5.71 (m, 1H), 5.20 (s, 2H), 4.28 – 4.16 (m, 3H), 4.16 – 4.04 (m, 2H), 3.80 (s, 3H), 3.40 (p, *J* = 5.6 Hz, 1H), 2.90 – 2.82 (m, 1H), 2.38 (dd, *J* = 18.0, 8.9 Hz, 1H), 1.90 (s, 3H), 1.53 (dd, *J* = 13.4, 6.2 Hz, 4H), 1.28 (t, *J* = 7.1 Hz, 3H), 0.90 (q, *J* = 7.3 Hz, 6H).

*Ethyl (3R,4R,5S)-4-acetamido-3-(pentan-3-yloxy)-5-(1-(4-(trifluoromethyl)benzyl)-1H-pyrazole-4-carboxamido)cyclohex-1-ene-1-carboxylate (**5g**)*

Yield 76%; white solid. ^{1}H NMR (400 MHz, $CDCl_3$) δ 7.85 (d, *J* = 3.9 Hz, 2H), 7.60 (d, *J* = 8.1 Hz, 2H), 7.32 (d, *J* = 8.0 Hz, 2H), 7.21 (d, *J* = 6.8 Hz, 1H), 6.81 (s, 1H), 6.02 (s, 1H), 5.33 (s, 2H), 4.29 – 4.16 (m, 3H), 4.16 – 4.06 (m, 2H), 3.47 – 3.32 (m, 1H), 2.95 – 2.83 (m, 1H), 2.45 – 2.31 (m, 1H), 1.90 (s, 3H), 1.60 – 1.47 (m, 4H), 1.28 (t, *J* = 7.1 Hz, 3H), 0.90 (q, *J* = 7.3 Hz, 6H).

*Ethyl (3R,4R,5S)-4-acetamido-3-(pentan-3-yloxy)-5-(1-(o-tolyl)-1H-pyrazole-4-carboxamido)cyclohex-1-ene-1-carboxylate (**5h**)*

Yield 73%; white solid. ^{1}H NMR (400 MHz, $CDCl_3$) δ 8.02 (s, 1H), 7.99 (s, 1H), 7.32 (dt, *J* = 13.1, 7.5 Hz, 4H), 6.84 (s, 1H), 6.00 (d, *J* = 7.8 Hz, 1H), 4.35 – 4.26 (m, 1H), 4.21 (dt, *J* = 15.8, 7.8 Hz, 3H), 4.13 (t, *J* = 6.5 Hz, 1H), 3.48 – 3.37 (m, 1H), 2.93 (dd, *J* = 17.9, 4.9 Hz, 1H), 2.44 (dd, *J* = 18.1, 9.1 Hz, 1H), 2.24 (s, 3H), 1.96 (s, 3H), 1.54 (dd, *J* = 13.5, 6.9 Hz, 4H), 1.30 (t, *J* = 7.1 Hz, 3H), 0.91 (dt, *J* = 12.5, 7.0 Hz, 8H).

*Ethyl (3R,4R,5S)-4-acetamido-3-(pentan-3-yloxy)-5-(1-(p-tolyl)-1H-pyrazole-4-carboxamido)cyclohex-1-ene-1-carboxylate (**5i**)*

Yield 66%; white solid. ^{1}H NMR (400 MHz, $CDCl_3$) δ 8.32 (s, 1H), 7.98 (s, 1H), 7.58 (d, *J* = 8.4 Hz, 2H), 7.27 – 7.21 (m, 2H), 6.87 (s, 1H), 5.76 (s, 1H), 4.35 – 4.19 (m, 4H), 4.14 (dd, *J* = 14.3, 7.1 Hz, 2H), 3.46 – 3.37 (m, 1H), 2.95 (dd, *J* = 18.1, 5.0 Hz, 1H), 2.53 – 2.43 (m, 1H), 2.41 (s, 3H), 1.99 (s, 3H), 1.57 (dd, *J* = 14.6, 7.4 Hz, 4H), 1.34 – 1.29 (m, 3H), 0.95 (td, *J* = 7.4, 4.9 Hz, 6H).

*Ethyl (3R,4R,5S)-4-acetamido-5-(1-(4-methoxyphenyl)-1H-pyrazole-4-carboxamido)-3-(pentan-3-yloxy)cyclohex-1-ene-1-carboxylate (**5j**)*

Yield 70%; white solid. ^{1}H NMR (400 MHz, $CDCl_3$) δ 8.25 (s, 1H), 7.97 (s, 1H), 7.56 (d, *J* = 8.8 Hz, 2H), 7.29 (d, *J* = 8.0 Hz, 1H), 6.96 (d, *J* = 8.9 Hz, 2H), 6.83 (s, 1H), 6.02 (d, *J* = 7.9 Hz, 1H), 4.36 – 4.26 (m, 1H), 4.26 – 4.16 (m, 3H), 4.16 – 4.04 (m, 1H), 3.84 (s, 3H), 3.42 (p, *J* = 5.4 Hz, 1H), 2.91 (dd, *J* = 18.0, 4.9 Hz, 1H), 2.44 (dd, *J* = 18.0, 9.3 Hz, 1H), 1.95 (s, 3H), 1.54 (dd, *J* = 13.6, 6.9 Hz, 4H), 1.30 (t, *J* = 7.1 Hz, 3H), 0.92 (q, *J* = 7.1 Hz, 6H).

*Ethyl (3R,4R,5S)-4-acetamido-5-(1-(4-chlorophenyl)-1H-pyrazole-4-carboxamido)-3-(pentan-3-yloxy)cyclohex-1-ene-1-carboxylate (**5k**)*

Yield 88%; white solid. ^{1}H NMR (400 MHz, $CDCl_3$) δ 8.35 (s, 1H), 8.00 (s, 1H), 7.62 (d, *J* = 8.9 Hz, 2H), 7.46 – 7.36 (m, 3H), 6.83 (s, 1H), 6.01 (d, *J* = 8.0 Hz, 1H), 4.35 – 4.25 (m, 1H), 4.25 – 4.10 (m, 4H), 3.41 (p, *J* = 5.7 Hz, 1H), 2.96 – 2.88 (m, 1H), 2.44 (dd, *J* = 18.0, 9.3 Hz, 1H), 1.95 (s, 3H), 1.59 – 1.50 (m, 4H), 1.30 (t, *J* = 7.1 Hz, 3H), 0.92 (q, *J* = 7.3 Hz, 6H).

*Ethyl (3R,4R,5S)-4-acetamido-3-(pentan-3-yloxy)-5-(3-phenyl-1H-pyrazole-5-carboxamido)cyclohex-1-ene-1-carboxylate (**11a**)*

Yield 67%; white solid. ^{1}H NMR (400 MHz, $CDCl_3$) δ 13.40 (s, 1H), 9.55 (s, 1H), 7.75 (d, *J* = 7.5 Hz, 2H), 7.40 (t, *J* = 7.6 Hz, 2H), 7.32 (t, *J* = 7.3 Hz, 1H), 7.04 (s, 1H), 6.75 (s, 1H), 6.48 (s, 1H), 4.45 – 4.34 (m, 1H), 4.25 – 4.07 (m, 4H), 3.44 – 3.35 (m, 1H), 2.88 – 2.82 (m, 1H), 2.45 – 2.29 (m, 1H), 2.03 (s, 3H), 1.50 (ddd, *J* = 10.9, 10.0, 6.4 Hz, 4H), 1.23 (t, *J* = 7.1 Hz, 3H), 0.95 – 0.82 (m, 6H).

*Ethyl (3R,4R,5S)-4-acetamido-5-(3-(4-chlorophenyl)-1H-pyrazole-5-carboxamido)-3-(pentan-3-yloxy)cyclohex-1-ene-1-carboxylate (**11b**)*

Yield 62%; white solid. ^{1}H NMR (400 MHz, $CDCl_3$) δ 13.50 (s, 1H), 9.56 (s, 1H), 7.71 (d, *J* = 8.5 Hz, 2H), 7.39 (d, *J* = 8.5 Hz, 2H), 7.03 (s, 1H), 6.77 (s, 1H), 6.52 (s, 1H), 4.41 (dt, *J* = 21.1, 7.9 Hz, 1H), 4.26 – 4.10 (m, 4H), 3.46 – 3.35 (m, 1H), 2.86 (d, *J* = 12.8 Hz, 1H), 2.45 – 2.31 (m, 1H), 2.04 (s, 3H), 1.54 (ddd, *J* = 12.4, 10.9, 5.4 Hz, 4H), 1.25 (t, *J* = 7.1 Hz, 3H), 0.91 (dt, *J* = 10.5, 7.4 Hz, 6H).

*Ethyl (3R,4R,5S)-4-acetamido-5-(3-(4-fluorophenyl)-1H-pyrazole-5-carboxamido)-3-(pentan-3-yloxy)cyclohex-1-ene-1-carboxylate (**11c**)*

Yield 71%; white solid. ^{1}H NMR (400 MHz, $CDCl_3$) δ 13.45 (s, 1H), 9.57 (s, 1H), 7.76 (dd, *J* = 8.6, 5.2 Hz, 2H), 7.11 (t, *J* = 8.6 Hz, 2H), 7.00 (s, 1H), 6.76 (s, 1H), 6.58 (d, *J* = 7.2 Hz, 1H), 4.50 – 4.36 (m, 1H), 4.28 – 4.08 (m, 4H), 3.45 – 3.36 (m, 1H), 2.90 – 2.80 (m, 1H), 2.38 (dd, *J* = 17.1, 11.2 Hz, 1H), 2.04 (s, 3H), 1.60 – 1.51 (m, 4H), 1.25 (t, *J* = 7.1 Hz, 3H), 0.91 (dt, *J* = 11.5, 7.4 Hz, 6H).

*Ethyl (3R,4R,5S)-4-acetamido-3-(pentan-3-yloxy)-5-(3-(4-(trifluoromethyl)phenyl)-1H-pyrazole-5-carboxamido)cyclohex-1-ene-1-carboxylate (**11d**)*

Yield 77%; white solid. ^{1}H NMR (400 MHz, $CDCl_3$) δ 13.86 (s, 1H), 9.58 (s, 1H), 7.89 (d, *J* = 8.2 Hz, 2H), 7.67 (d, *J* = 8.3 Hz, 2H), 7.11 (s, 1H), 6.89 (s, 1H), 6.76 (s, 1H), 4.51 – 4.35 (m, 1H), 4.27 – 4.06 (m, 4H), 3.50 – 3.33 (m, 1H), 2.88 (dd, *J* = 17.5, 4.8 Hz, 1H), 2.44 – 2.31 (m, 1H), 2.06

(s, 3H), 1.60 – 1.45 (m, 4H), 1.23 (t, *J* = 7.1 Hz, 3H), 0.92 (t, *J* = 7.4 Hz, 3H), 0.85 (t, *J* = 7.4 Hz, 3H).

*Ethyl (3R,4R,5S)-4-acetamido-5-(3-(4-methoxyphenyl)-1H-pyrazole-5-carboxamido)-3-(pentan-3-yloxy)cyclohex-1-ene-1-carboxylate (**11e**)*

Yield 65%; white solid. ^{1}H NMR (400 MHz, $CDCl_3$) δ 13.20 (s, 1H), 9.63 (s, 1H), 7.68 (d, *J* = 8.7 Hz, 2H), 6.93 (d, *J* = 7.8 Hz, 3H), 6.75 (s, 1H), 6.41 (d, *J* = 8.7 Hz, 1H), 4.47 – 4.34 (m, 1H), 4.24 – 4.03 (m, 4H), 3.82 (s, 3H), 3.44 – 3.33 (m, 1H), 2.91 – 2.83 (m, 1H), 2.38 (dd, *J* = 17.2, 11.2 Hz, 1H), 2.02 (s, 3H), 1.57 – 1.47 (m, 4H), 1.23 (t, *J* = 7.1 Hz, 3H), 0.88 (dt, *J* = 14.6, 7.4 Hz, 6H).

*Ethyl (3R,4R,5S)-4-acetamido-3-(pentan-3-yloxy)-5-(3-(thiophen-2-yl)-1H-pyrazole-5-carboxamido)cyclohex-1-ene-1-carboxylate (**11f**)*

Yield 45%; white solid. ^{1}H NMR (400 MHz, $CDCl_3$) δ 9.45 (s, 1H), 7.52 (d, *J* = 3.2 Hz, 1H), 7.31 (d, *J* = 4.8 Hz, 1H), 7.11 – 7.04 (m, 1H), 6.91 (s, 1H), 6.78 (s, 1H), 6.59 (d, *J* = 8.5 Hz, 1H), 4.40 (qd, *J* = 10.4, 5.0 Hz, 1H), 4.26 – 4.15 (m, 4H), 3.45 – 3.37 (m, 1H), 2.92 – 2.84 (m, 1H), 2.49 – 2.34 (m, 1H), 2.04 (s, 3H), 1.60 – 1.51 (m, 4H), 1.26 (t, *J* = 7.1 Hz, 3H), 0.91 (dt, *J* = 10.2, 7.4 Hz, 6H).

*Methyl 3-(4-chlorophenyl)-1-(cyclopropylmethyl)-1H-pyrazole-5-carboxylate (**13a**)*

Yield 75%; colorless oil. ^{1}H NMR (400 MHz, $CDCl_3$) δ 7.73 (d, *J* = 8.5 Hz, 2H), 7.36 (d, *J* = 8.5 Hz, 2H), 4.45 (t, *J* = 7.7 Hz, 2H), 3.90 (s, 3H), 1.46 – 1.37 (m, 1H), 0.56 – 0.49 (m, 2H), 0.48 – 0.39 (m, 2H).

*Methyl 3-(4-chlorophenyl)-1-(cyclobutylmethyl)-1H-pyrazole-5-carboxylate (**13b**)*

Yield 68%; colorless oil. ^{1}H NMR (400 MHz, $CDCl_3$) δ 7.73 (d, *J* = 8.5 Hz, 2H), 7.37 (d, *J* = 8.5 Hz, 2H), 7.08 (s, 1H), 4.63 (d, *J* = 7.3 Hz, 2H), 3.91 (s, 3H), 2.88 (dt, *J* = 15.2, 7.4 Hz, 1H), 2.08 – 1.95 (m, 2H), 1.95 – 1.80 (m, 4H).

*Methyl 3-(4-chlorophenyl)-1-(cyclohexylmethyl)-1H-pyrazole-5-carboxylate (**13c**)*

Yield 70%; colorless oil. ^{1}H NMR (400 MHz, $CDCl_3$) δ 7.75 (d, *J* = 8.5 Hz, 2H), 7.39 (d, *J* = 8.5 Hz, 2H), 7.11 (s, 1H), 4.47 (d, *J* = 7.3 Hz, 2H), 3.93 (s, 3H), 2.03 – 1.90 (m, 1H), 1.76 – 1.59 (m, 5H), 1.26 – 1.02 (m, 5H).

*Ethyl (3R,4R,5S)-4-acetamido-5-(3-(4-chlorophenyl)-1-(cyclopropylmethyl)-1H-pyrazole-5-carboxamido)-3-(pentan-3-yloxy)cyclohex-1-ene-1-carboxylate (**15a**)*

Yield 75%; white solid. ^{1}H NMR (400 MHz, $CDCl_3$) δ 7.70 (d, *J* = 8.4 Hz, 2H), 7.48 (d, *J* = 7.0 Hz, 1H), 7.35 (d, *J* = 8.5 Hz, 2H), 6.86 (s, 1H), 6.80 (s, 1H), 5.67 (s, 1H), 4.45 (d, *J* = 7.1 Hz, 2H), 4.30 – 4.16 (m, 4H), 4.08 (d, *J* = 6.4 Hz, 1H), 3.42 (p, *J* = 5.6 Hz, 1H), 2.90 (dd, *J* = 17.9, 4.3 Hz, 1H), 2.47 (dd, *J* = 17.8, 8.0 Hz, 1H), 1.99 (s, 3H), 1.60 – 1.51 (m, 4H), 1.46 – 1.35 (m, 1H), 1.30 (t, *J* = 7.1 Hz, 3H), 0.93 (dd, *J* = 12.7, 7.3 Hz, 6H), 0.55 – 0.48 (m, 2H), 0.47 – 0.39 (m, 2H).

*Ethyl (3R,4R,5S)-4-acetamido-5-(3-(4-chlorophenyl)-1-(cyclobutylmethyl)-1H-pyrazole-5-carboxamido)-3-(pentan-3-yloxy)cyclohex-1-ene-1-carboxylate (**15b**)*

Yield 43%; white solid. ^{1}H NMR (400 MHz, $CDCl_3$) δ 7.70 (d, *J* = 8.5 Hz, 2H), 7.41 (d, *J* = 7.0 Hz, 1H), 7.35 (d, *J* = 8.5 Hz, 2H), 6.86 (s, 1H), 6.77 (s, 1H), 5.59 (s, 1H), 4.67 – 4.53 (m, 2H), 4.32 – 4.16 (m, 4H), 4.08 (d, *J* = 6.0 Hz, 1H), 3.48 – 3.36 (m, 1H), 2.97 – 2.80 (m, 2H), 2.46 (dd, *J* = 17.9, 8.0 Hz, 1H), 2.06 – 1.93 (m, 5H), 1.86 (s, 4H), 1.59 – 1.49 (m, 4H), 1.30 (t, *J* = 7.1 Hz, 3H), 0.93 (td, *J* = 7.4, 4.7 Hz, 6H).

*Ethyl (3R,4R,5S)-4-acetamido-5-(3-(4-chlorophenyl)-1-(cyclohexylmethyl)-1H-pyrazole-5-carboxamido)-3-(pentan-3-yloxy)cyclohex-1-ene-1-carboxylate (**15c**)*

Yield 85%; white solid. ^{1}H NMR (400 MHz, $CDCl_3$) δ 7.72 (s, 1H), 7.70 (s, 1H), 7.40 (d, *J* = 7.2 Hz, 1H), 7.36 (d, *J* = 8.5 Hz, 2H), 6.86 (s, 1H), 6.77 (s, 1H), 5.55 (d, *J* = 7.1 Hz, 1H), 4.43 (dd, *J* = 7.3, 1.3 Hz, 2H), 4.27 – 4.21 (m, 3H), 4.07 (d, *J* = 5.5 Hz, 1H), 3.47 – 3.38 (m, 1H), 2.90 (dd, *J* = 17.9, 4.3 Hz, 1H), 2.53 – 2.40 (m, 1H), 2.00 (s, 3H), 1.98 – 1.89 (m, 1H), 1.75 – 1.62 (m, 3H), 1.57 – 1.48 (m, 6H), 1.30 (t, *J* = 7.1 Hz, 3H), 1.21 (dd, *J* = 22.0, 8.3 Hz, 3H), 1.10 – 0.98 (m, 2H), 0.93 (td, *J* = 7.4, 4.2 Hz, 6H).

*Ethyl 5-cyclopropyl-1-phenyl-1H-pyrazole-3-carboxylate (**19a**)*

Yield 52%; Colorless jelly. ^{1}H NMR (400 MHz, $CDCl_3$) δ 7.67 – 7.58 (m, 2H), 7.53 – 7.46 (m, 2H), 7.46 – 7.39 (m, 1H), 6.50 (s, 1H), 4.41 (q, *J* = 7.2 Hz, 2H), 1.84 – 1.73 (m, 1H), 1.39 (t, *J* = 7.1 Hz, 3H), 1.04 – 0.96 (m, 2H), 0.84 – 0.75 (m, 2H).

*Ethyl 5-cyclopropyl-1-(o-tolyl)-1H-pyrazole-3-carboxylate (**19b**)*

Yield 40%; Colorless oil. ^{1}H NMR (400 MHz, $CDCl_3$) δ 7.41 – 7.29 (m, 4H), 6.44 (s, 1H), 4.40 (q, *J* = 7.2 Hz, 2H), 2.09 (s, 3H), 1.47 (td, *J* = 8.4, 4.2 Hz, 1H), 1.39 (t, *J* = 7.1 Hz, 3H), 0.96 – 0.86 (m, 2H), 0.78 – 0.67 (m, 2H).

*Ethyl 5-cyclopropyl-1-(m-tolyl)-1H-pyrazole-3-carboxylate (**19c**)*

Yield 39%; colorless jelly. ^{1}H NMR (400 MHz, $CDCl_3$) δ 7.50 – 7.32 (m, 3H), 7.23 (d, *J* = 7.4 Hz, 1H), 6.48 (s, 1H), 4.40 (q, *J* = 7.1 Hz, 2H), 2.42 (s, 3H), 1.86 – 1.71 (m, 1H), 1.39 (t, *J* = 7.1 Hz, 3H), 1.08 – 0.92 (m, 2H), 0.79 (dt, *J* = 6.7, 4.6 Hz, 2H).

*Ethyl 1-(4-bromophenyl)-5-cyclopropyl-1H-pyrazole-3-carboxylate (**19d**)*

Yield 48%; orange jelly. ^{1}H NMR (400 MHz, $CDCl_3$) δ 7.62 (d, *J* = 8.7 Hz, 2H), 7.53 (d, *J* = 8.7 Hz, 2H), 6.51 (s, 1H), 4.41 (q, *J* = 7.1 Hz, 2H), 1.81 – 1.70 (m, 1H), 1.39 (t, *J* = 7.1 Hz, 3H), 1.06 – 0.97 (m, 2H), 0.83 – 0.72 (m, 2H).

*Ethyl 5-cyclopropyl-1-(4-(trifluoromethyl)phenyl)-1H-pyrazole-3-carboxylate (**19e**)*

Yield 82%; white solid. ^{1}H NMR (400 MHz, $CDCl_3$) δ 7.82 (d, *J* = 8.6 Hz, 2H), 7.77 (d, *J* = 8.6 Hz, 2H), 6.56 (s, 1H), 3.94 (s, 3H), 1.85 – 1.75 (m, 1H), 1.11 – 1.03 (m, 2H), 0.88 – 0.80 (m, 2H).

*Methyl 3-cyclopropyl-1-phenyl-1H-pyrazole-5-carboxylate (**20a**)*

Yield 32%; colorless jelly. ^{1}H NMR (400 MHz, $CDCl_3$) δ 7.62 (d, *J* = 7.6 Hz, 2H), 7.54 – 7.45 (m, 2H), 7.45 – 7.39 (dd, *J* = 8.1, 6.5 Hz, 1H), 6.51 (d, *J* = 4.2 Hz, 1H), 3.92 (s, 3H), 1.86 – 1.73 (m, 1H), 1.07 – 0.95 (m, 2H), 0.86 – 0.73 (m, 2H).

*Methyl 3-cyclopropyl-1-(o-tolyl)-1H-pyrazole-5-carboxylate (**20b**)*

Yield 12%; colorless oil. ^{1}H NMR (400 MHz, $CDCl_3$) δ 7.43 – 7.28 (m, 4H), 6.46 (s, 1H), 3.91 (s, 3H), 2.09 (s, 3H), 1.53 – 1.43 (m, 1H), 0.93 – 0.87 (m, 2H), 0.75 – 0.69 (m, 2H).

*Methyl 3-cyclopropyl-1-(m-tolyl)-1H-pyrazole-5-carboxylate (**20c**)*

Yield 35%; colorless jelly. ^{1}H NMR (400 MHz, $CDCl_3$) δ 7.47 – 7.38 (m, 2H), 7.35 (t, *J* = 7.6 Hz, 1H), 7.23 (d, *J* = 7.3 Hz, 1H), 6.49 (s, 1H), 3.92 (s, 3H), 2.42 (s, 3H), 1.86 – 1.72 (m, 1H), 1.11 – 0.93 (m, 2H), 0.88 – 0.71 (m, 2H).

*Methyl 1-(4-bromophenyl)-3-cyclopropyl-1H-pyrazole-5-carboxylate (**20d**)*

Yield 16%; orange jelly. ^{1}H NMR (400 MHz, $CDCl_3$) δ 7.62 (d, *J* = 8.7 Hz, 2H), 7.53 (d, *J* = 8.7 Hz, 2H), 6.52 (s, 1H), 3.93 (s, 3H), 1.81 – 1.70 (m, 1H), 1.06 – 0.98 (m, 2H), 0.84 – 0.75 (m, 2H).

*Ethyl (3R,4R,5S)-4-acetamido-5-(5-cyclopropyl-1-phenyl-1H-pyrazole-3-carboxamido)-3-(pentan-3-yloxy)cyclohex-1-ene-1-carboxylate (**23a**)*

Yield 66%; white solid. ^{1}H NMR (400 MHz, $CDCl_3$) δ 7.63 (d, *J* = 7.8 Hz, 2H), 7.50 (t, *J* = 7.7 Hz, 3H), 7.42 (t, *J* = 7.3 Hz, 1H), 6.85 (s, 1H), 6.44 (s, 1H), 5.87 (d, *J* = 8.9 Hz, 1H), 4.41 – 4.29 (m, 1H), 4.27 – 4.15 (m, 3H), 4.06 (d, *J* = 6.6 Hz, 1H), 3.45 – 3.32 (m, 1H), 2.76 (d, *J* = 4.8 Hz, 1H), 2.51 (dd, *J* = 17.9, 8.9 Hz, 1H), 1.91 (s, 3H), 1.79 (td, *J* = 8.4, 4.3 Hz, 1H), 1.51 (dd, *J* = 13.6, 6.9 Hz, 4H), 1.28 (t, *J* = 7.2 Hz, 3H), 1.05 – 0.94 (m, 2H), 0.88 (dd, *J* = 16.6, 7.6 Hz, 6H), 0.83 – 0.72 (m, 2H).

*Ethyl (3R,4R,5S)-4-acetamido-5-(5-cyclopropyl-1-(o-tolyl)-1H-pyrazole-3-carboxamido)-3-(pentan-3-yloxy)cyclohex-1-ene-1-carboxylate (**23b**)*

Yield 83%; white solid. ^{1}H NMR (400 MHz, $CDCl_3$) δ 7.45 – 7.30 (m, 5H), 6.84 (s, 1H), 6.38 (s, 1H), 5.84 (d, *J* = 9.0 Hz, 1H), 4.33 (qd, *J* = 9.3, 5.4 Hz, 1H), 4.25 – 4.13 (m, 3H), 4.09 – 4.00 (m, 1H), 3.42 – 3.31 (m, 1H), 2.79 – 2.73 (m, 1H), 2.53 – 2.40 (m, 1H), 2.10 (s, 3H), 1.90 (s, 3H), 1.55 – 1.41 (m, 5H), 1.28 (t, *J* = 7.1 Hz, 3H), 0.92 – 0.80 (m, 8H), 0.75 – 0.67 (m, 2H).

*Ethyl (3R,4R,5S)-4-acetamido-5-(5-cyclopropyl-1-(m-tolyl)-1H-pyrazole-3-carboxamido)-3-(pentan-3-yloxy)cyclohex-1-ene-1-carboxylate (**23c**)*

Yield 80%; white solid. ^{1}H NMR (400 MHz, $CDCl_3$) δ 7.44 (t, *J* = 6.6 Hz, 2H), 7.37 (dd, *J* = 14.3, 6.9 Hz, 2H), 7.23 (d, *J* = 7.3 Hz, 1H), 6.85 (s, 1H), 6.43 (s, 1H), 5.84 (d, *J* = 9.2 Hz, 1H), 4.41 – 4.28 (m, 1H), 4.25 – 4.15 (m, 3H), 4.06 (d, *J* = 7.5 Hz, 1H), 3.38 (p, *J* = 5.5 Hz, 1H), 2.76 (d, *J* = 5.7 Hz, 1H), 2.56 – 2.47 (m, 1H), 2.44 (s, 3H), 1.91 (s, 3H), 1.78 (ddd, *J* = 13.3, 8.4, 5.1 Hz, 1H), 1.52 (ddd, *J* = 12.7, 6.6, 3.8 Hz, 4H), 1.28 (t, *J* = 7.1 Hz, 3H), 1.04 – 0.96 (m, 2H), 0.88 (dt, *J* = 10.2, 7.4 Hz, 6H), 0.83 – 0.75 (m, 2H).

*Ethyl (3R,4R,5S)-4-acetamido-5-(1-(4-bromophenyl)-5-cyclopropyl-1H-pyrazole-3-carboxamido)-3-(pentan-3-yloxy)cyclohex-1-ene-1-carboxylate (**23d**)*

Yield 79%; white solid. ^{1}H NMR (400 MHz, $CDCl_3$) δ7.62 (d, *J* = 8.7 Hz, 2H), 7.53 (d, *J* = 8.7 Hz, 2H), 7.48 (d, *J* = 8.6 Hz, 1H), 7.26 (s, 3H), 6.85 (s, 1H), 6.46 (s, 1H), 5.77 (d, *J* = 9.0 Hz, 1H), 4.33 (ddd, *J* = 18.0, 9.0, 5.3 Hz, 1H), 4.26 – 4.15 (m, 3H), 4.09 – 3.96 (m, 1H), 3.39 (p, *J* = 5.6 Hz, 1H), 2.78 – 2.72 (m, 1H), 2.55 – 2.40 (m, 1H), 1.91 (s, 3H), 1.76 (ddd, *J* = 13.5, 8.5, 5.2 Hz, 1H), 1.57 – 1.47 (m, 4H), 1.28 (t, *J* = 7.1 Hz, 3H), 1.02 (dd, *J* = 8.2, 2.0 Hz, 2H), 0.88 (dt, *J* = 10.6, 7.4 Hz, 6H), 0.83 – 0.76 (m, 2H).

*Ethyl (3R,4R,5S)-4-acetamido-5-(5-cyclopropyl-1-(4-(trifluoromethyl)phenyl)-1H-pyrazole-3-carboxamido)-3-(pentan-3-yloxy)cyclohex-1-ene-1-carboxylate (**23e**)*

Yield 68%; white solid. ^{1}H NMR (400 MHz, $CDCl_3$) δ7.83 (d, *J* = 8.4 Hz, 2H), 7.76 (d, *J* = 8.5 Hz, 2H), 7.54 (d, *J* = 8.6 Hz, 1H), 6.86 (s, 1H), 6.50 (s, 1H), 5.80 (d, *J* = 8.8 Hz, 1H), 4.40 – 4.28 (m, 1H), 4.27 – 4.16 (m, 3H), 4.06 (d, *J* = 6.6 Hz, 1H), 3.45 – 3.36 (m, 1H), 2.78 (d, *J* = 5.7 Hz, 1H), 2.52 (dd, *J* = 17.9, 8.7 Hz, 1H), 1.92 (s, 3H), 1.86 – 1.76 (m, 1H), 1.60 – 1.47 (m, 4H), 1.29 (t, *J* = 7.1 Hz, 3H), 1.06 (d, *J* = 8.2 Hz, 2H), 0.89 (dd, *J* = 15.8, 7.7 Hz, 6H), 0.85 – 0.77 (m, 2H).

*Ethyl (3R,4R,5S)-4-acetamido-5-(3-cyclopropyl-1-phenyl-1H-pyrazole-5-carboxamido)-3-(pentan-3-yloxy)cyclohex-1-ene-1-carboxylate (**25a**)*

Yield 50%; white solid. ^{1}H NMR (400 MHz, $CDCl_3$) ^{1}H NMR (400 MHz, $CDCl_3$) δ7.65 (d, *J* = 7.7 Hz, 2H), 7.56 – 7.41 (m, 4H), 6.87 (s, 1H), 6.47 (s, 1H), 5.76 (d, *J* = 9.2 Hz, 1H), 4.42 – 4.31 (m, 1H), 4.31 – 4.19 (m, 3H), 4.07 (d, *J* = 7.7 Hz, 1H), 3.44 – 3.36 (m, 1H), 2.78 (s, 1H), 2.56 (d, *J* = 9.0 Hz, 1H), 1.94 (s, 3H), 1.85 – 1.77 (m, 1H), 1.57 – 1.49 (m, 4H), 1.30 (t, *J* = 7.7 Hz, 3H), 1.08 – 0.97 (m, 2H), 0.95 – 0.85 (m, 6H), 0.84 – 0.74 (m, 2H).

*Ethyl (3R,4R,5S)-4-acetamido-5-(3-cyclopropyl-1-(o-tolyl)-1H-pyrazole-5-carboxamido)-3-(pentan-3-yloxy)cyclohex-1-ene-1-carboxylate (**25b**)*

Yield 68%; white solid. ^{1}H NMR (400 MHz, $CDCl_3$) δ7.43 – 7.30 (m, 5H), 6.84 (s, 1H), 6.38 (s, 1H), 5.81 (d, *J* = 9.1 Hz, 1H), 4.33 (dd, *J* = 9.5, 5.4 Hz, 1H), 4.25 – 4.13 (m, 3H), 4.07 – 3.99 (m, 1H), 3.42 – 3.29 (m, 1H), 2.78 – 2.68 (m, 1H), 2.51 – 2.39 (m, 1H), 2.10 (s, 3H), 1.90 (s, 3H), 1.54 – 1.43 (m, 5H), 1.28 (t, *J* = 7.1 Hz, 3H), 0.91 – 0.82 (m, 8H), 0.76 – 0.68 (m, 2H).

*Ethyl (3R,4R,5S)-4-acetamido-5-(3-cyclopropyl-1-(m-tolyl)-1H-pyrazole-5-carboxamido)-3-(pentan-3-yloxy)cyclohex-1-ene-1-carboxylate (**25c**)*

Yield 69%; white solid. ^{1}H NMR (400 MHz, $CDCl_3$) δ7.50 – 7.39 (m, 3H), 7.36 (t, *J* = 7.6 Hz, 1H), 7.23 (d, *J* = 7.4 Hz, 1H), 6.85 (s, 1H), 6.43 (s, 1H), 5.81 (d, *J* = 9.0 Hz, 1H), 4.34 (ddd, *J* = 18.5, 9.1, 5.4 Hz, 1H), 4.21 (dt, *J* = 14.4, 6.0 Hz, 3H), 4.05 (dd, *J* = 5.2, 2.3 Hz, 1H), 3.38 (p, *J* = 5.6 Hz, 1H), 2.78 – 2.73 (m, 1H), 2.56 – 2.47 (m, 1H), 2.44 (s, 3H), 1.91 (s, 3H), 1.83 – 1.69 (m, 1H), 1.57 – 1.47 (m, 4H), 1.28 (t, *J* = 7.1 Hz, 3H), 0.99 (d, *J* = 8.4 Hz, 2H), 0.88 (dt, *J* = 10.4, 7.4 Hz, 6H), 0.82 – 0.75 (m, 2H).

*Ethyl (3R,4R,5S)-4-acetamido-5-(1-(4-bromophenyl)-3-cyclopropyl-1H-pyrazole-5-carboxamido)-3-(pentan-3-yloxy)cyclohex-1-ene-1-carboxylate (**25d**)*

Yield 61%; white solid. ^{1}H NMR (400 MHz, $CDCl_3$) δ 7.68 – 7.59 (m, 2H), 7.58 – 7.51 (m, 2H), 7.49 (d, *J* = 8.1 Hz, 1H), 6.85 (s, 1H), 6.46 (s, 1H), 5.82 (d, *J* = 9.2 Hz, 1H), 4.39 – 4.29 (m, 1H), 4.26 – 4.16 (m, 3H), 4.06 (d, *J* = 6.8 Hz, 1H), 3.39 (p, *J* = 5.6 Hz, 1H), 2.78 – 2.75 (m, 1H), 2.58 – 2.45 (m, 1H), 1.91 (s, 3H), 1.76 (dd, *J* = 9.2, 4.2 Hz, 1H), 1.59 – 1.48 (m, 4H), 1.28 (t, *J* = 7.1 Hz, 3H), 1.02 (dd, *J* = 8.2, 1.9 Hz, 2H), 0.88 (dt, *J* = 10.2, 7.4 Hz, 6H), 0.83 – 0.76 (m, 2H).

2. ^{1}H and ^{13}C NMR Spectra of the products

*(3R,4R,5S)-4-acetamido-5-(1-benzyl-1H-pyrazole-4-carboxamido)-3-(pentan-3-yloxy)cyclohex-1-ene-1-carboxylic acid (**6a**)*

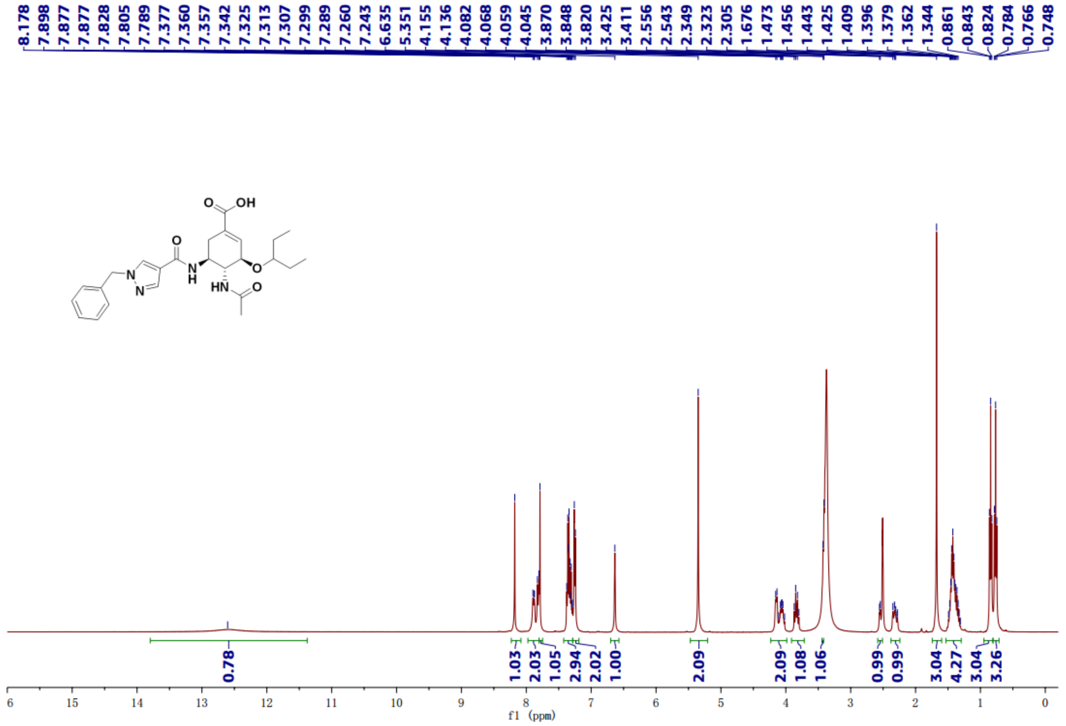

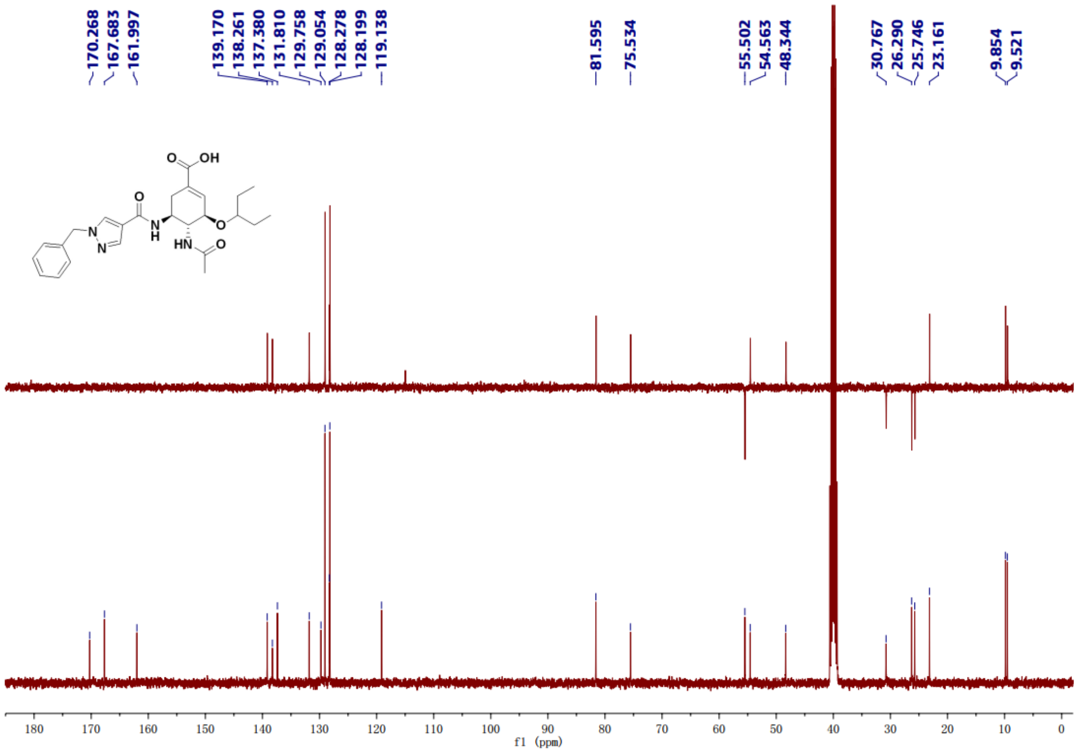

*(3R,4R,5S)-4-acetamido-5-(1-(2-chlorobenzyl)-1H-pyrazole-4-carboxamido)-3-(pentan-3-yloxy)cyclohex-1-ene-1-carboxylic acid (**6b**)*

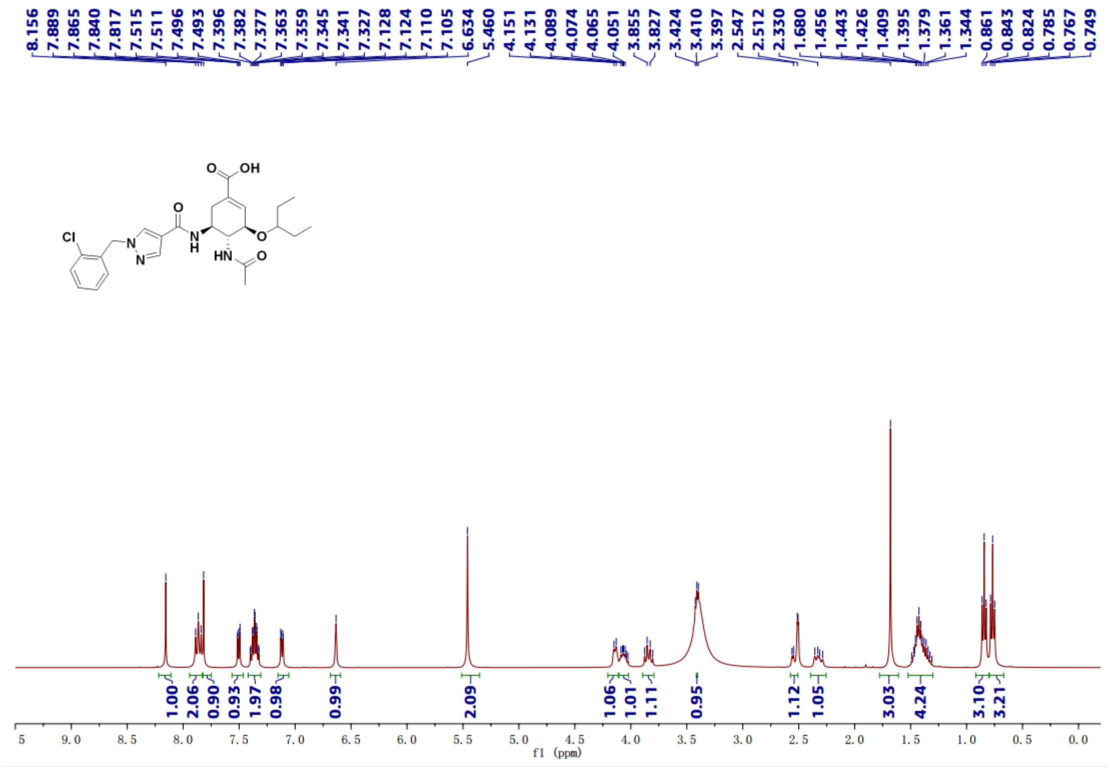

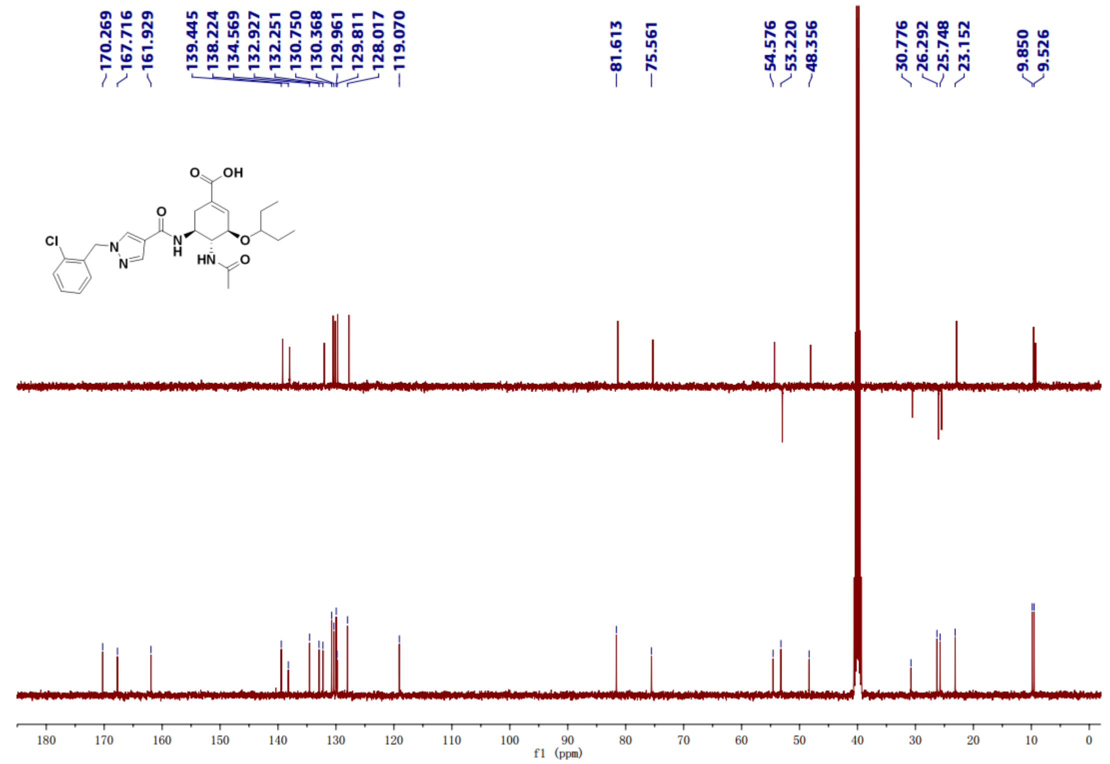

*(3R,4R,5S)-4-acetamido-5-(1-(3-chlorobenzyl)-1H-pyrazole-4-carboxamido)-3-(pentan-3-yloxy)cyclohex-1-ene-1-carboxylic acid (**6c**)*

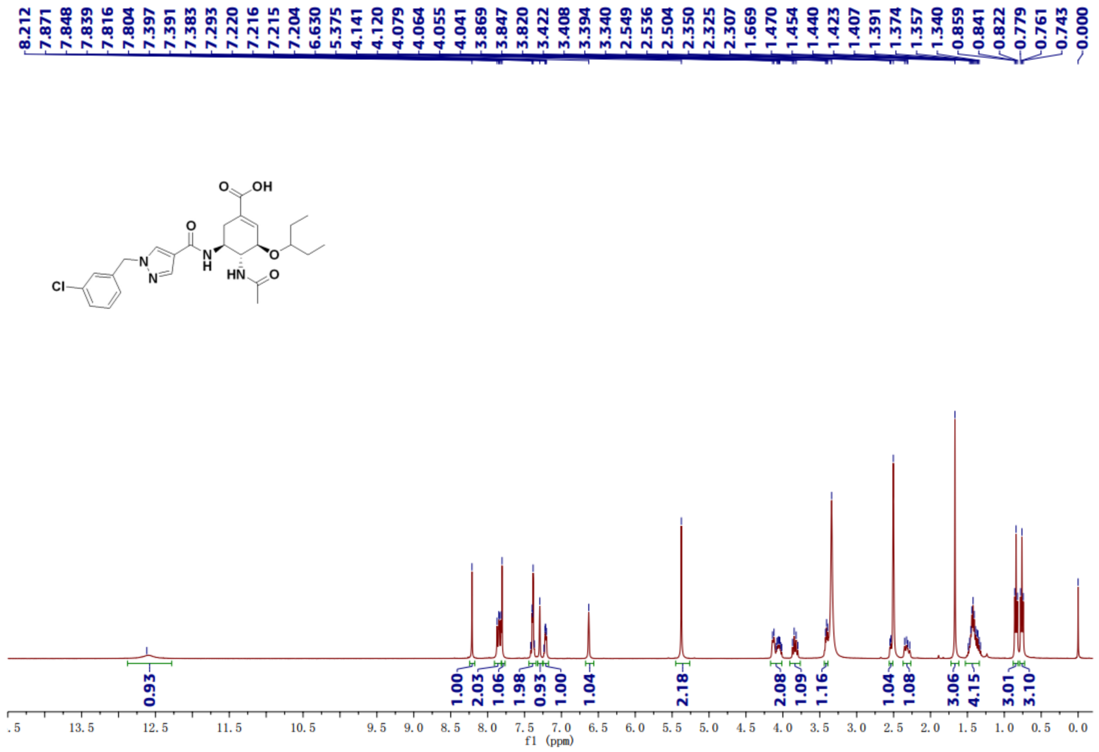

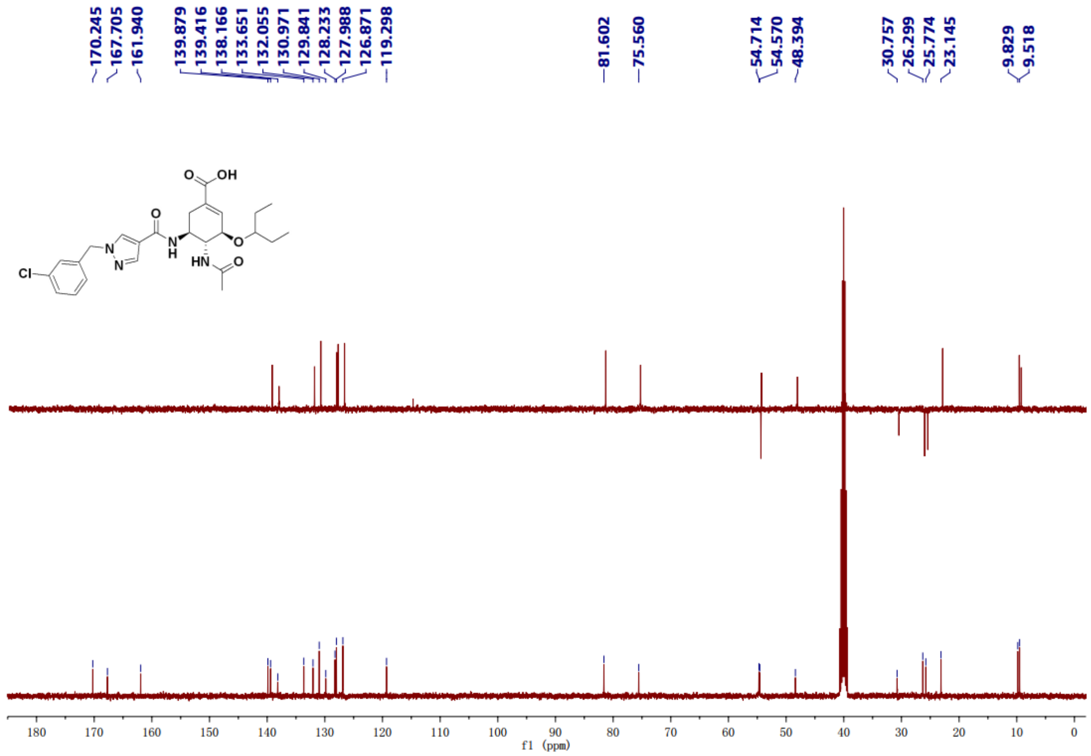

*(3R,4R,5S)-4-acetamido-5-(1-(3-nitrobenzyl)-1H-pyrazole-4-carboxamido)-3-(pentan-3-yloxy)cyclohex-1-ene-1-carboxylic acid (**6d**)*

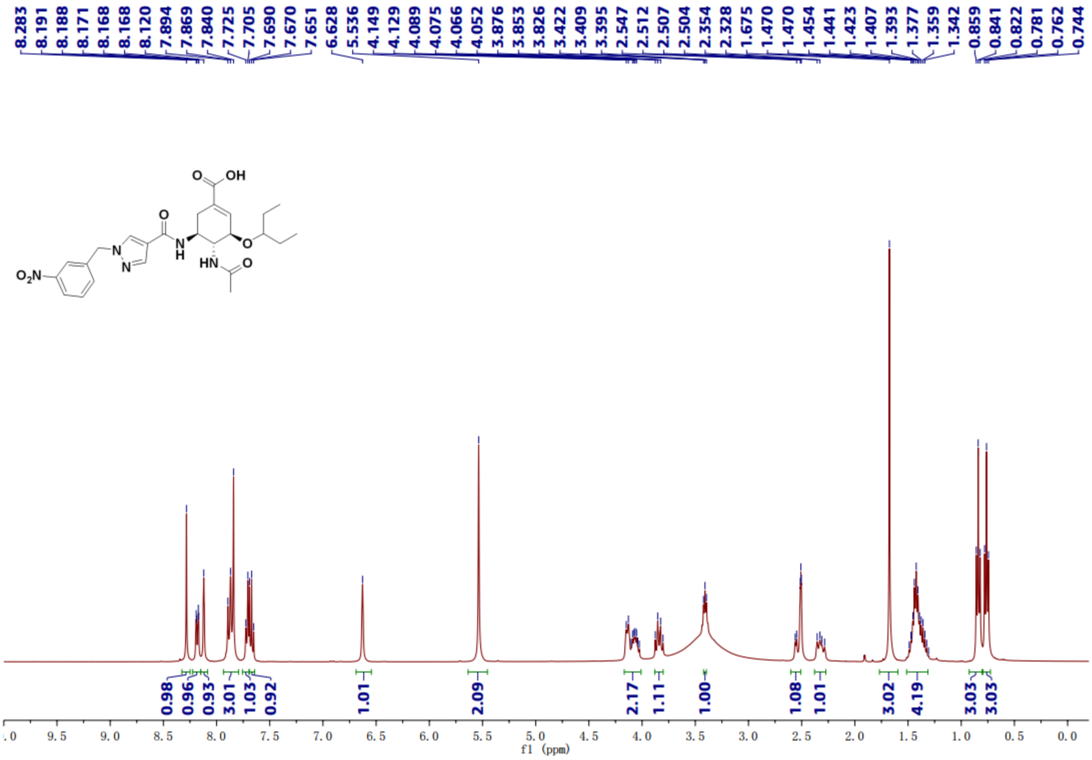

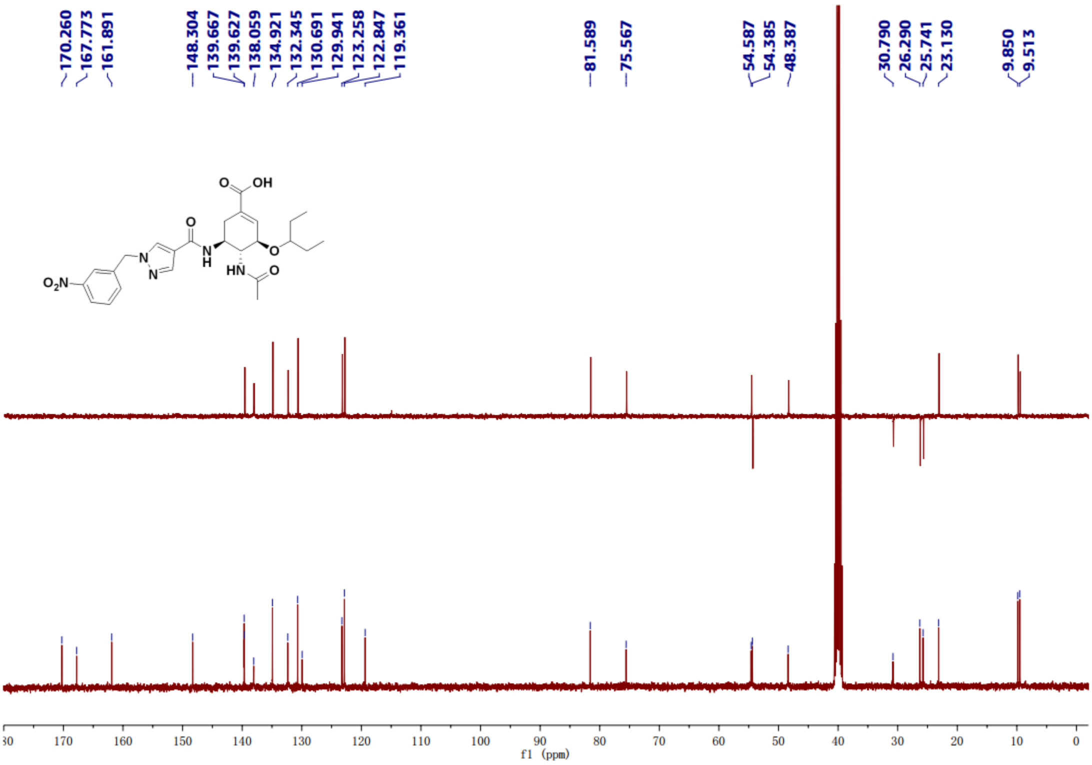

*(3R,4R,5S)-4-acetamido-5-(1-(3-methoxybenzyl)-1H-pyrazole-4-carboxamido)-3-(pentan-3-yloxy)cyclohex-1-ene-1-carboxylic acid (**6e**)*

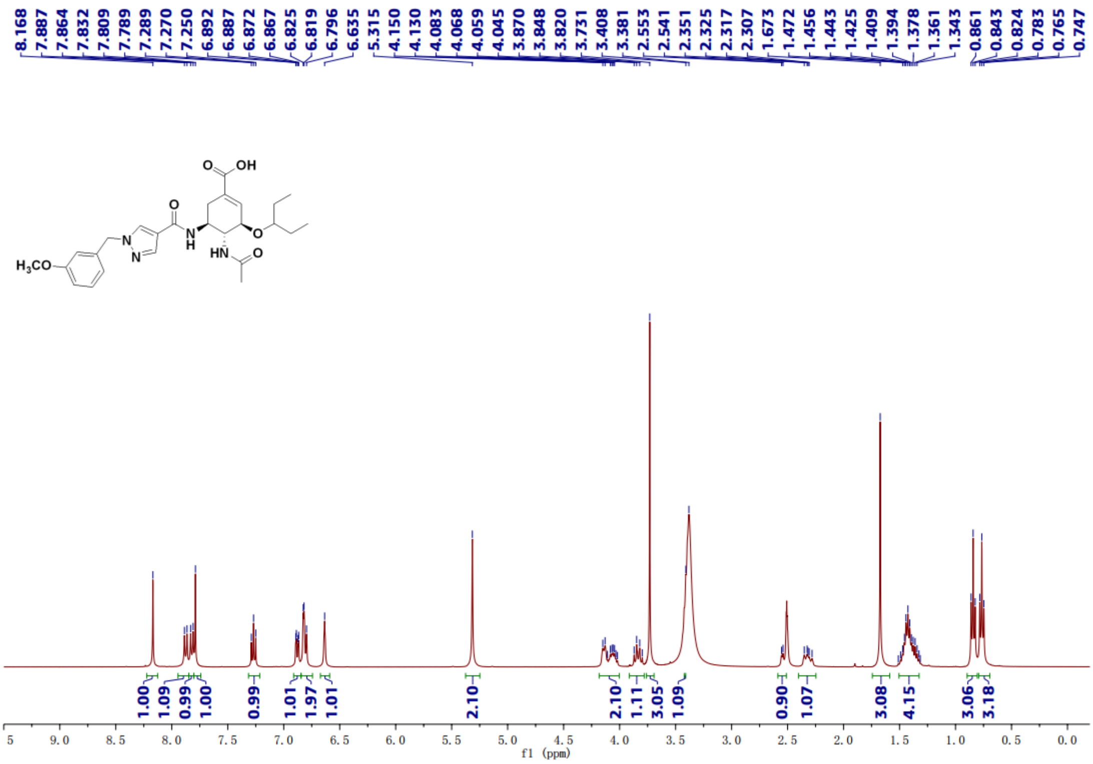

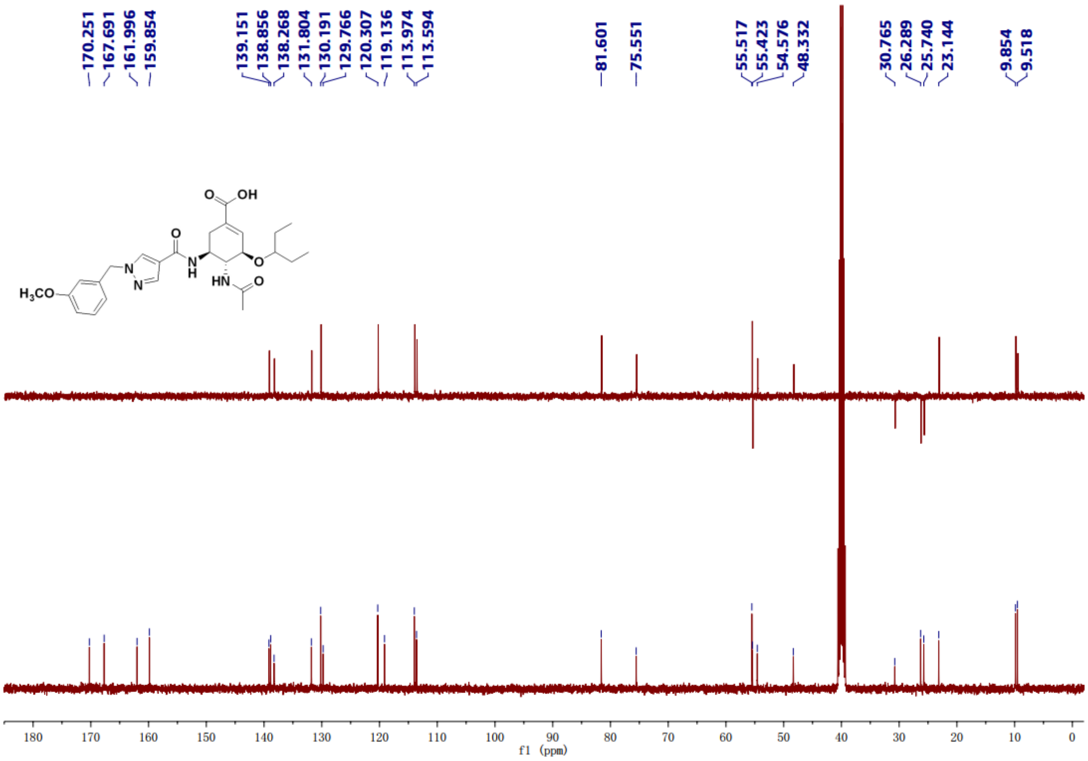

*(3R,4R,5S)-4-acetamido-5-(1-(4-methoxybenzyl)-1H-pyrazole-4-carboxamido)-3-(pentan-3-yloxy)cyclohex-1-ene-1-carboxylic acid (**6f**)*

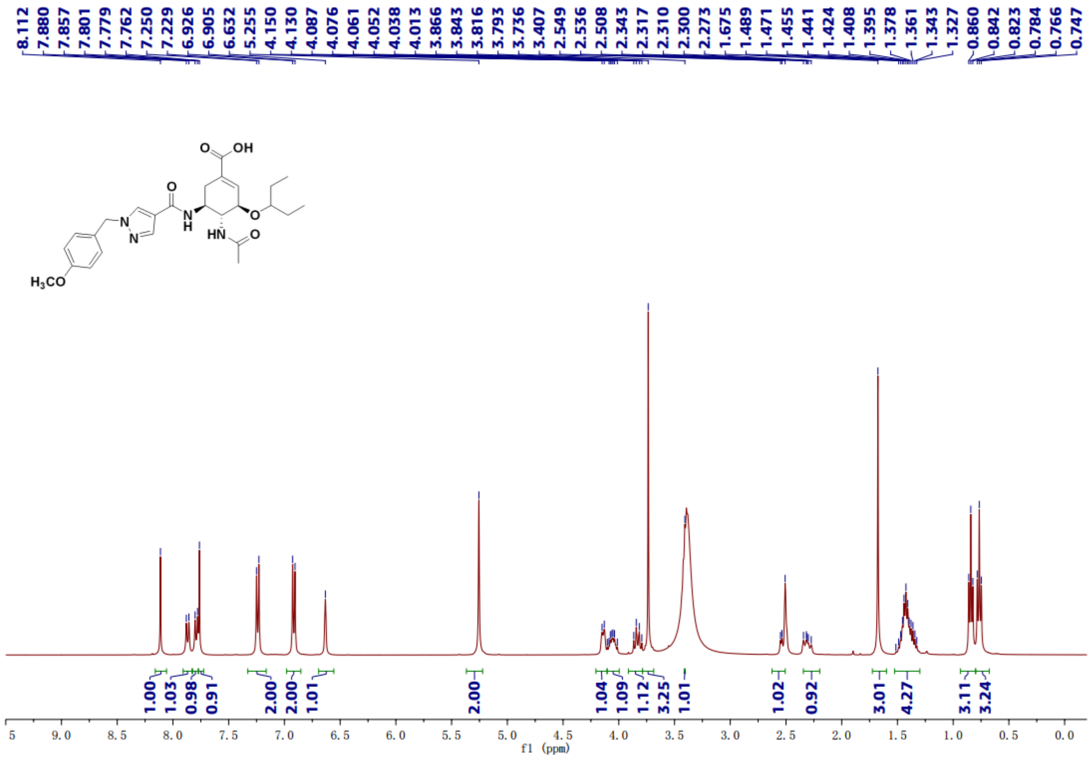

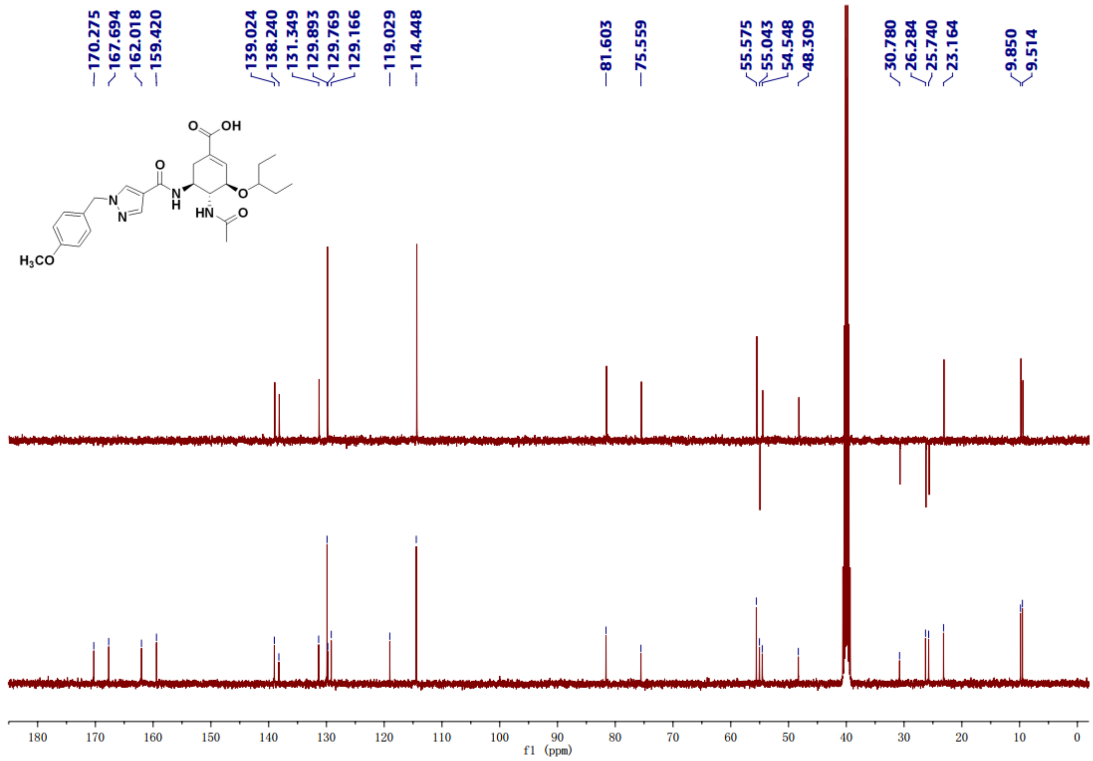

*(3R,4R,5S)-4-acetamido-3-(pentan-3-yloxy)-5-(1-(4-(trifluoromethyl)benzyl)-1H-pyrazole-4-carboxamido)cyclohex-1-ene-1-carboxylic acid (**6g**)*

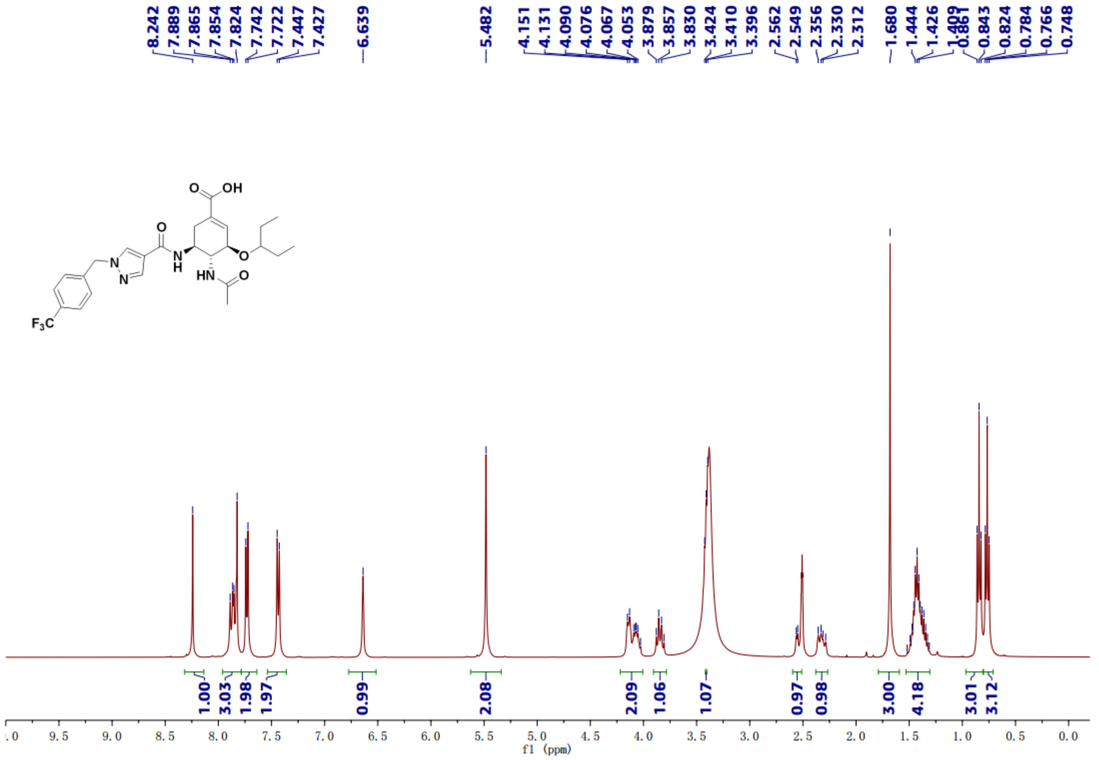

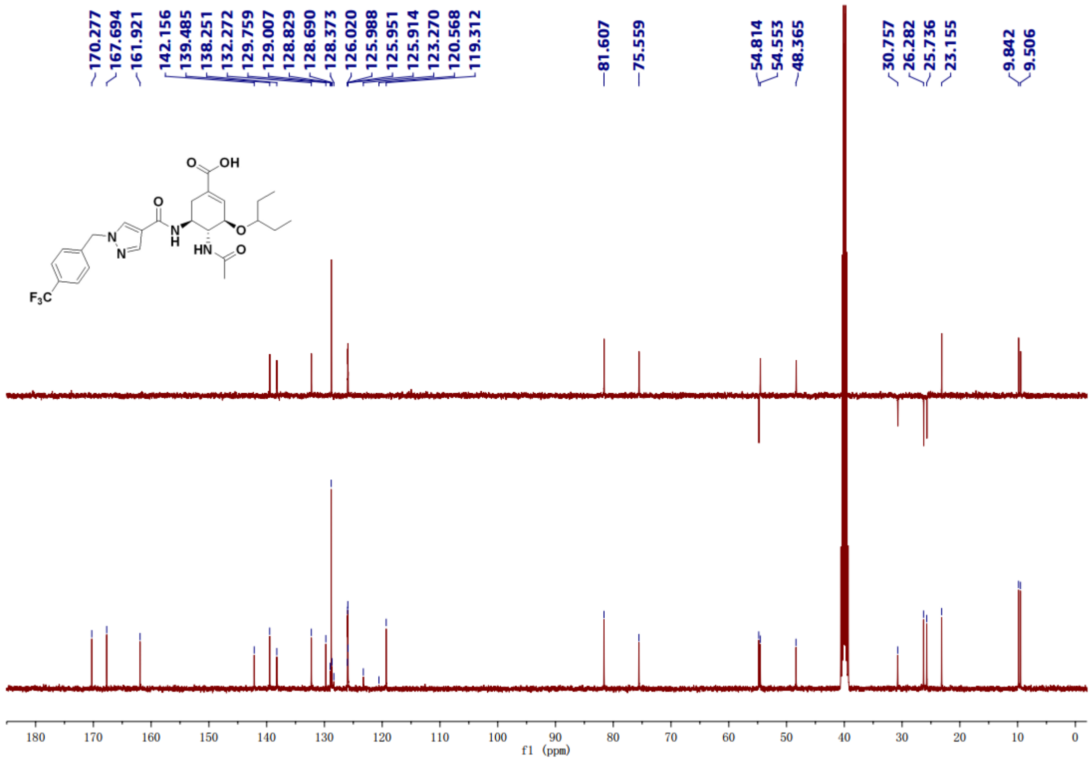

*(3R,4R,5S)-4-acetamido-3-(pentan-3-yloxy)-5-(1-(o-tolyl)-1H-pyrazole-4-carboxamido)cyclohex-1-ene-1-carboxylic acid (**6h**)*

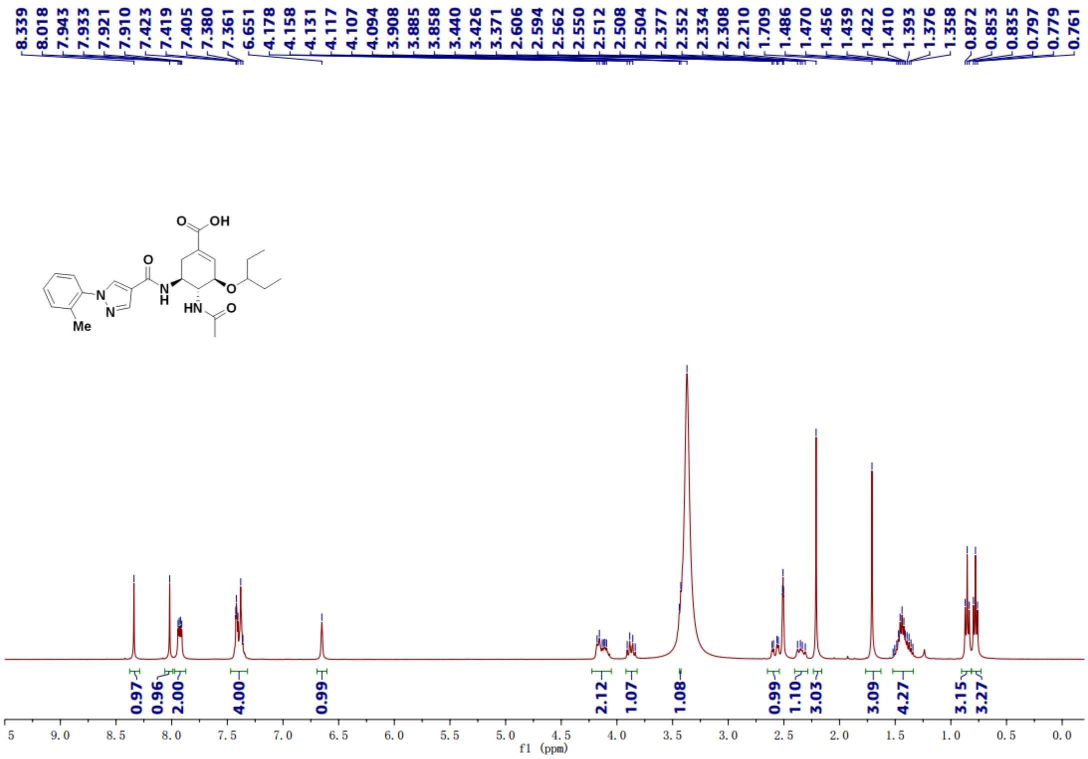

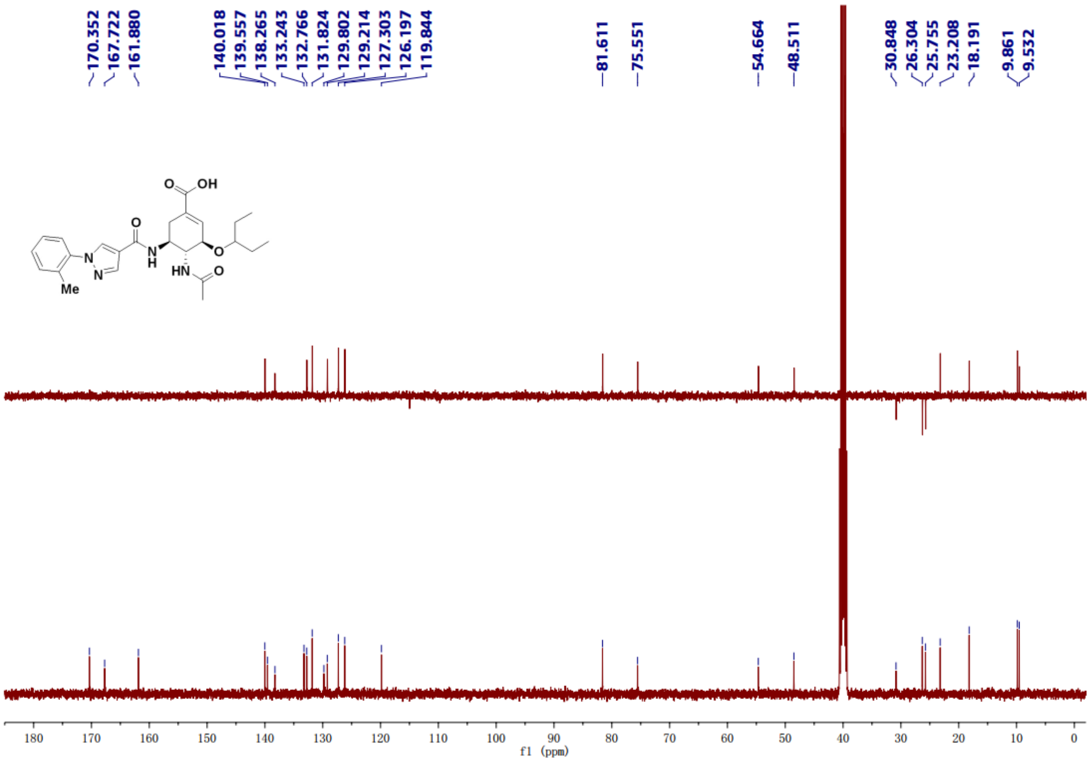

*(3R,4R,5S)-4-acetamido-3-(pentan-3-yloxy)-5-(1-(p-tolyl)-1H-pyrazole-4-carboxamido)cyclohex-1-ene-1-carboxylic acid (**6i**)*

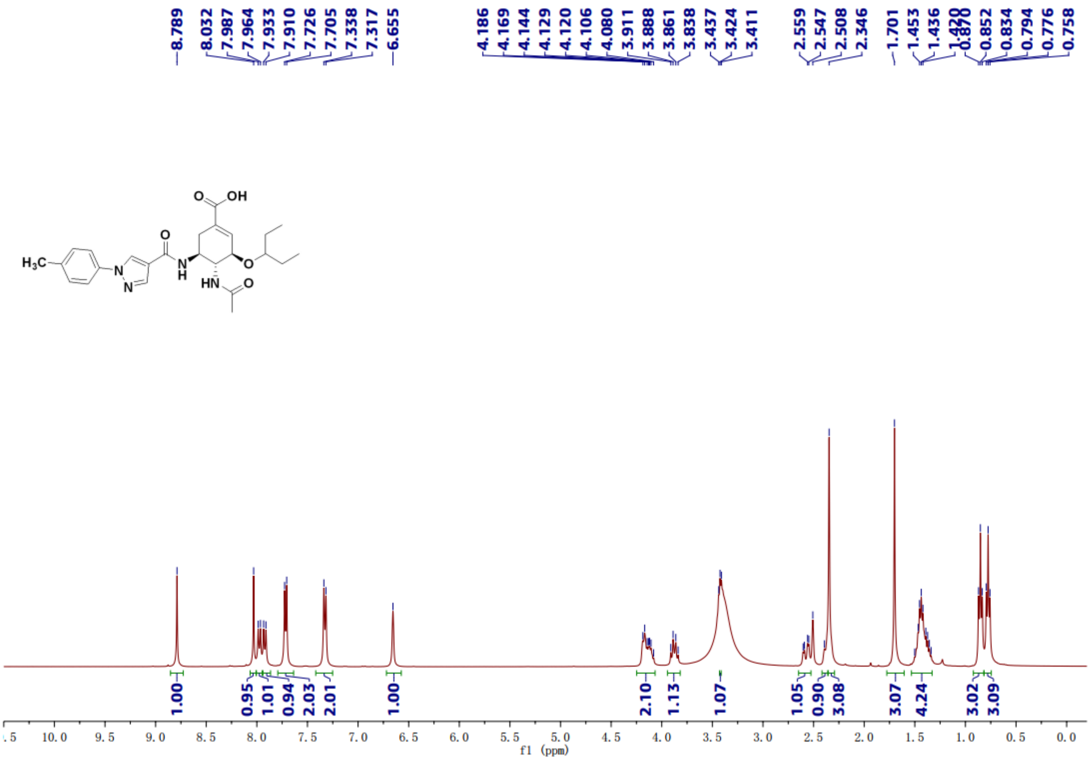

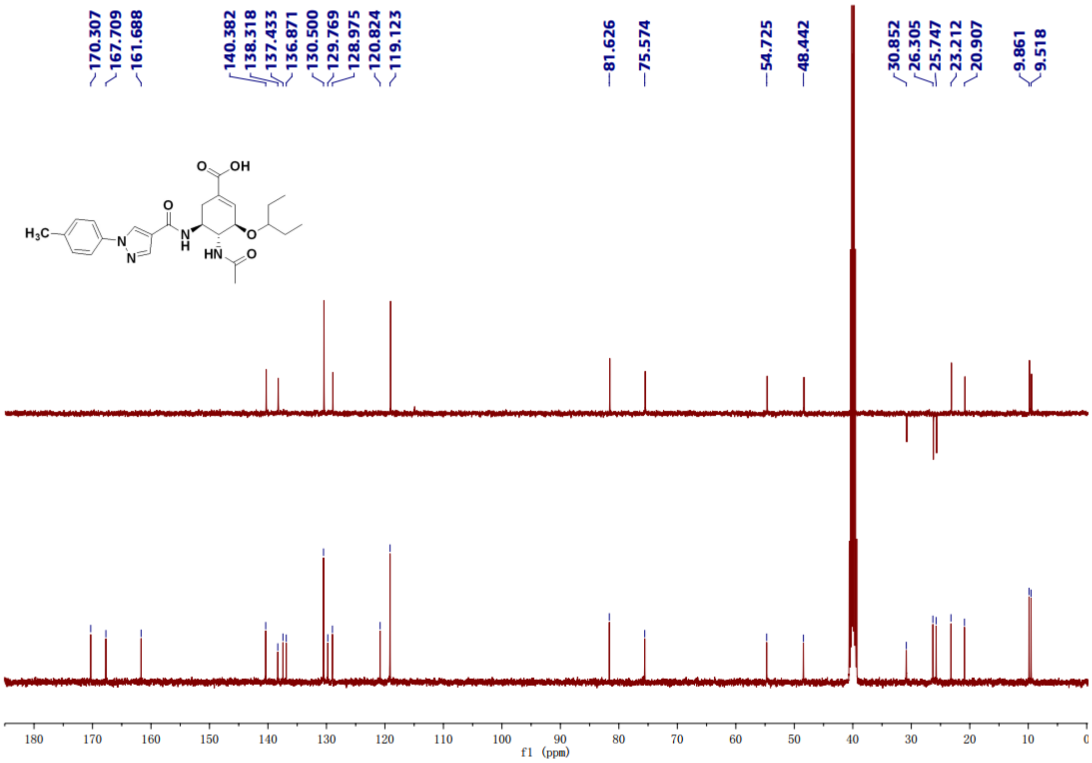

*(3R,4R,5S)-4-acetamido-5-(1-(4-methoxyphenyl)-1H-pyrazole-4-carboxamido)-3-(pentan-3-yloxy)cyclohex-1-ene-1-carboxylic acid (**6j**)*

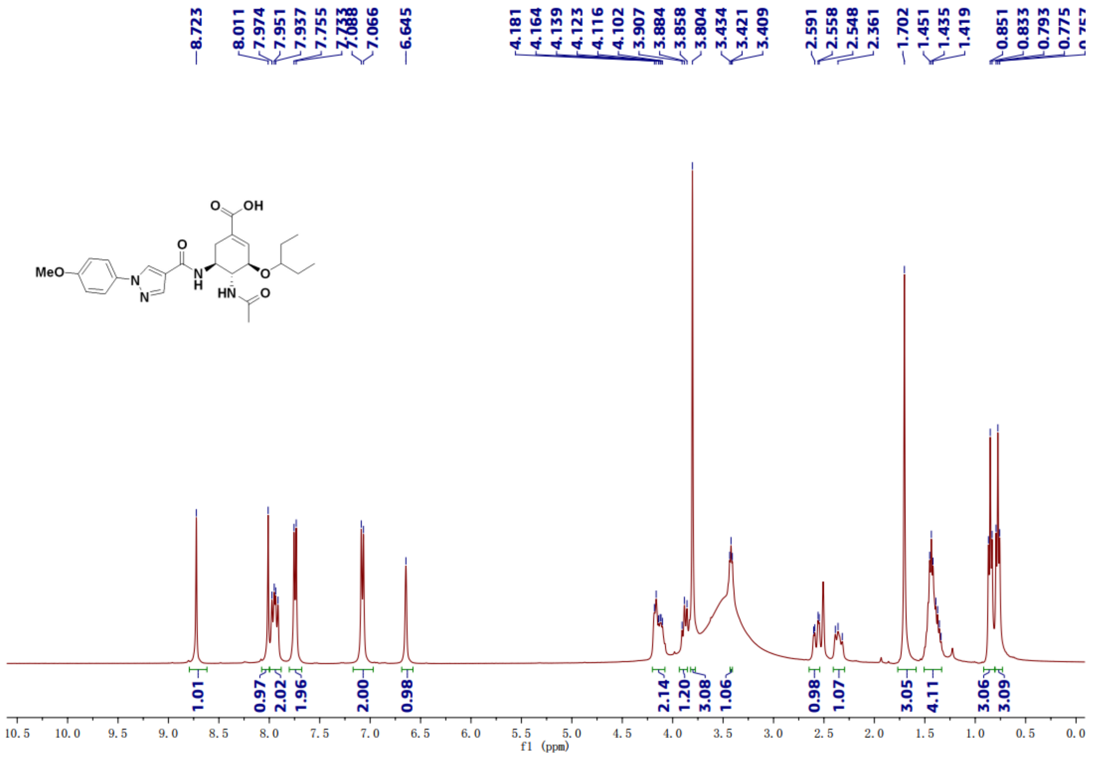

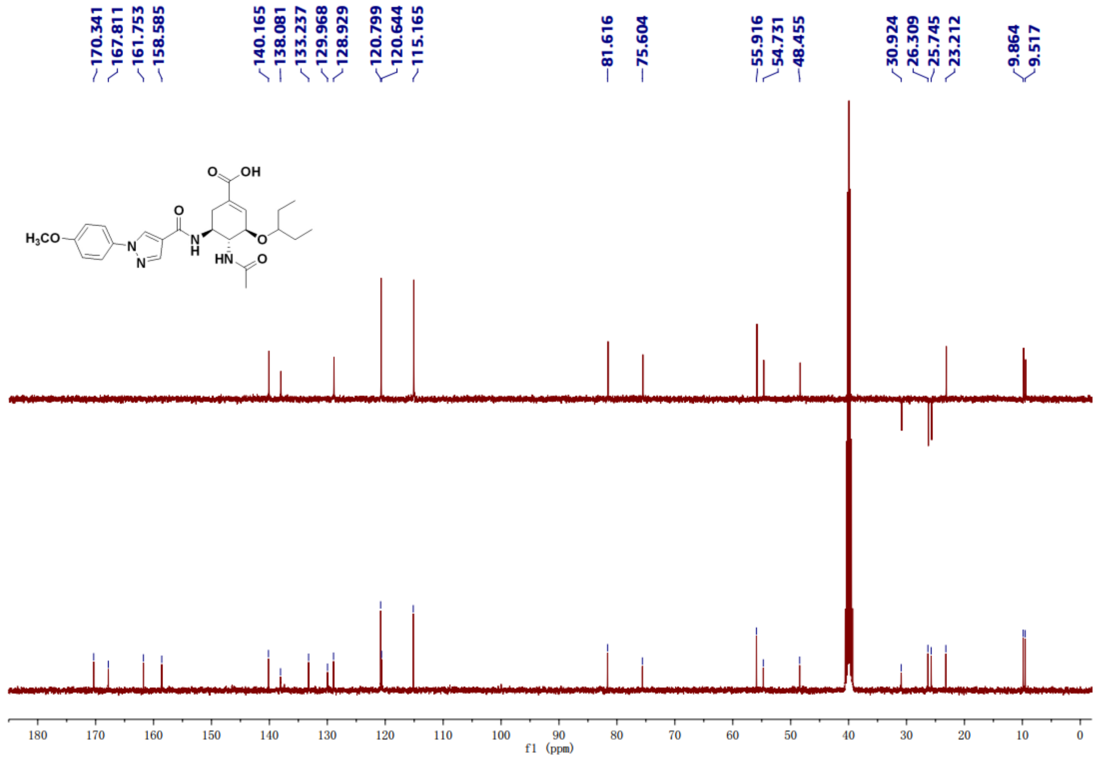

*(3R,4R,5S)-4-acetamido-5-(1-(4-chlorophenyl)-1H-pyrazole-4-carboxamido)-3-(pentan-3-yloxy)cyclohex-1-ene-1-carboxylic acid (**6k**)*

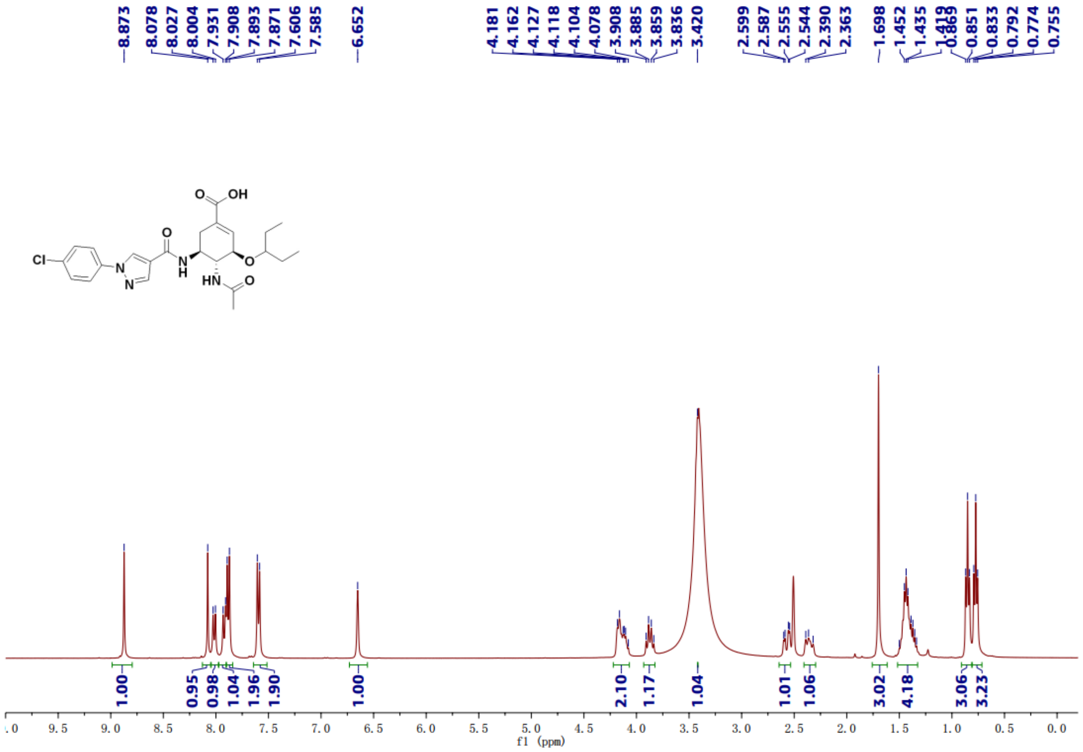

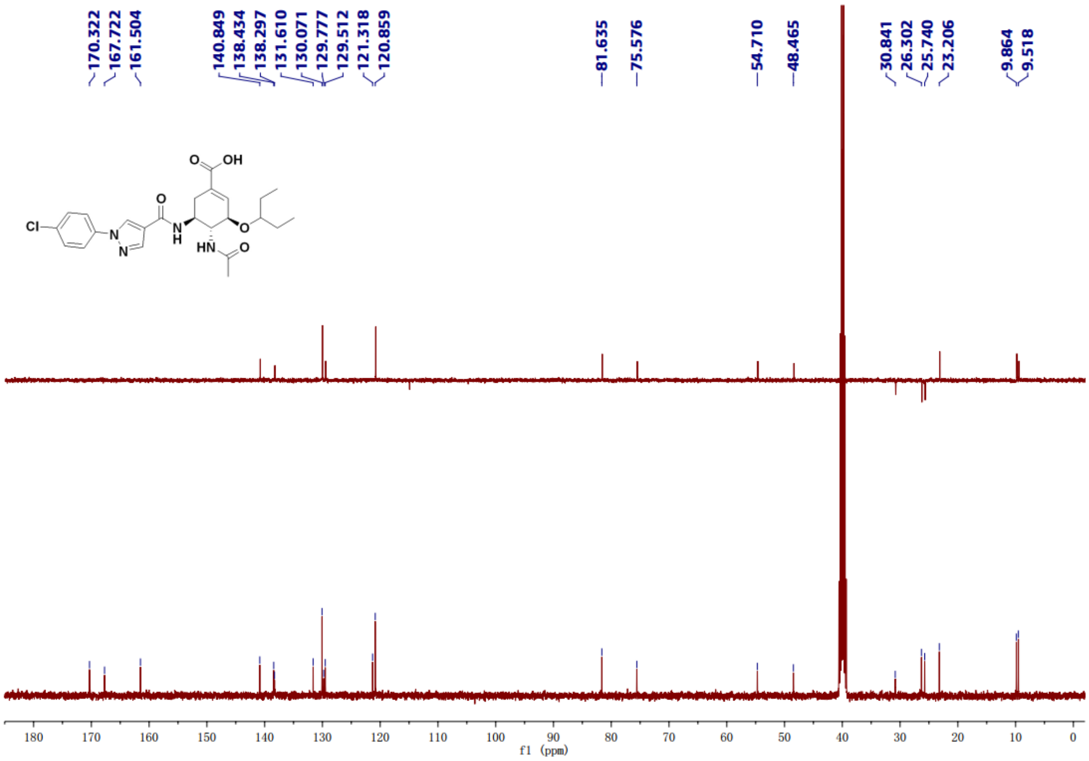

*(3R,4R,5S)-4-acetamido-3-(pentan-3-yloxy)-5-(3-phenyl-1H-pyrazole-5-carboxamido)cyclohex-1-ene-1-carboxylic acid (**12a**)*

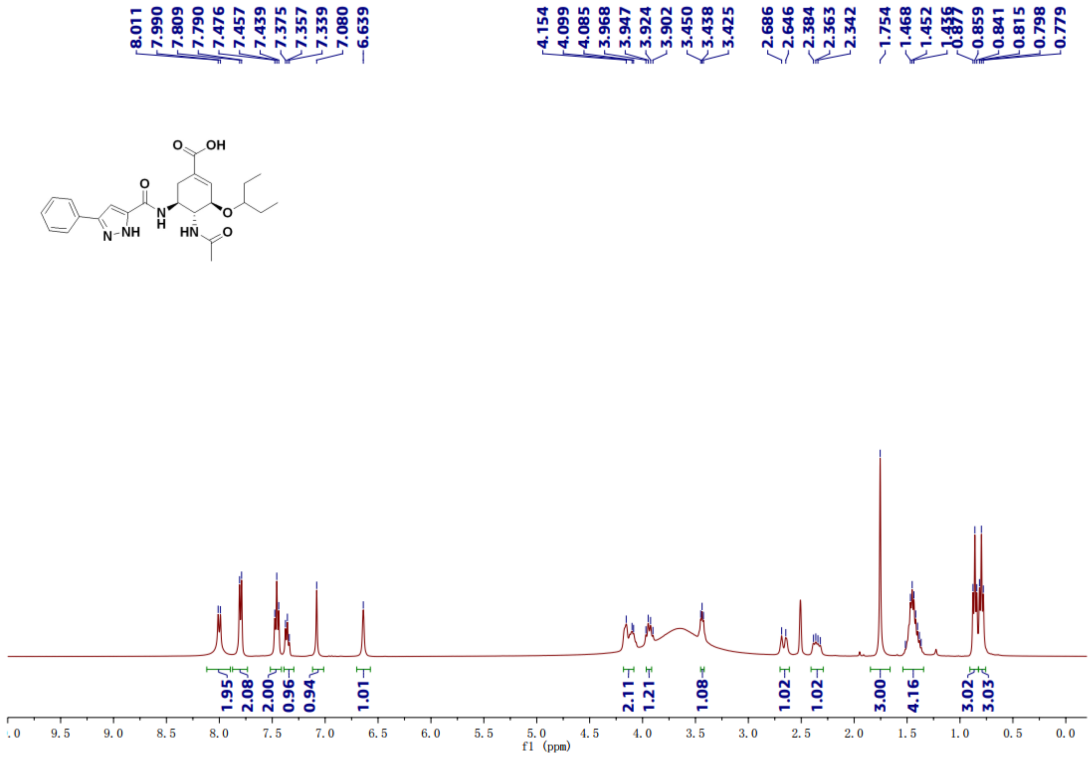

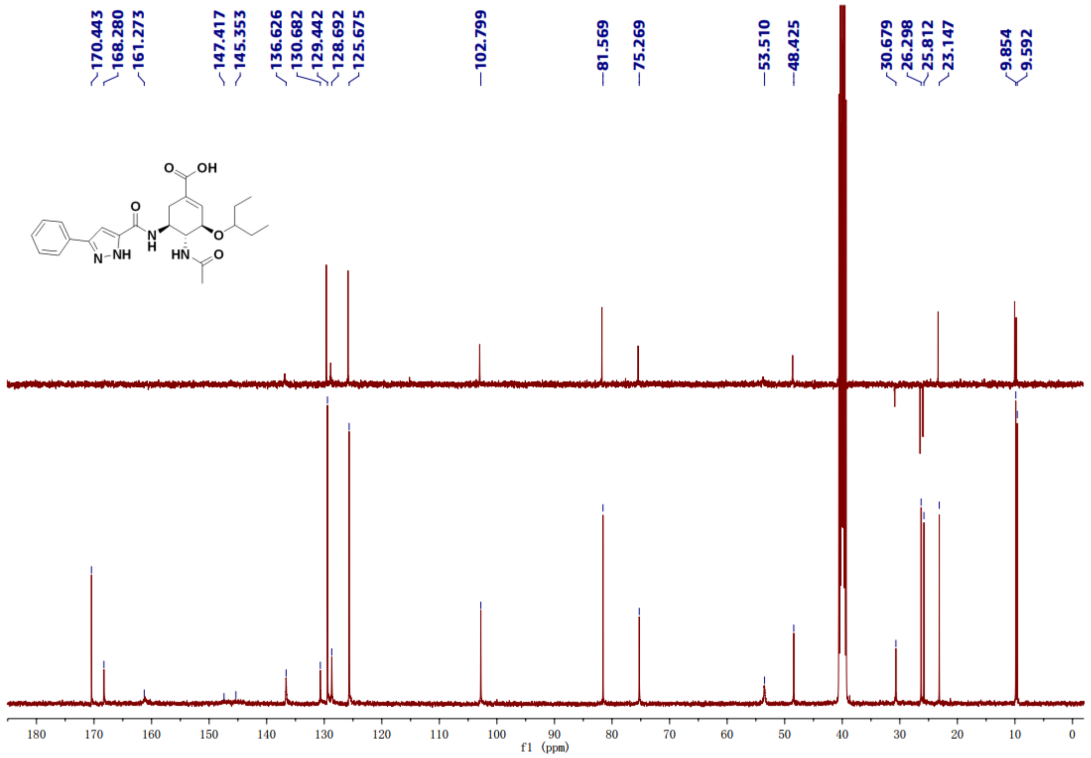

*(3R,4R,5S)-4-acetamido-5-(3-(4-chlorophenyl)-1H-pyrazole-5-carboxamido)-3-(pentan-3-yloxy)cyclohex-1-ene-1-carboxylic acid (**12b**)*

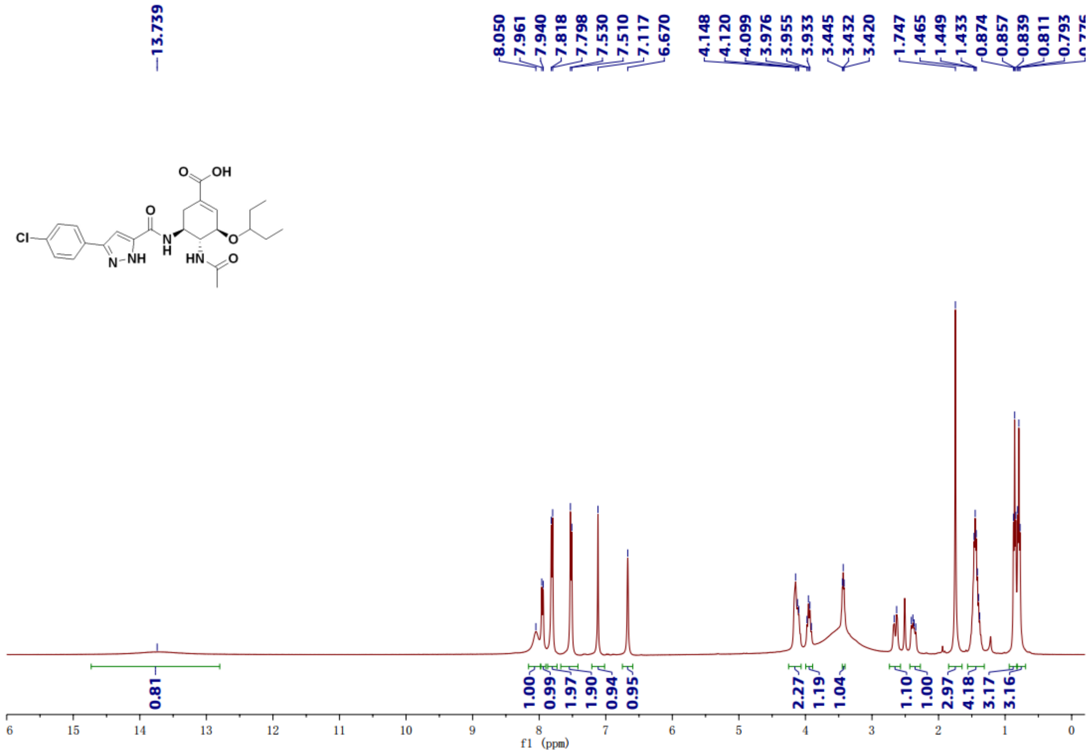

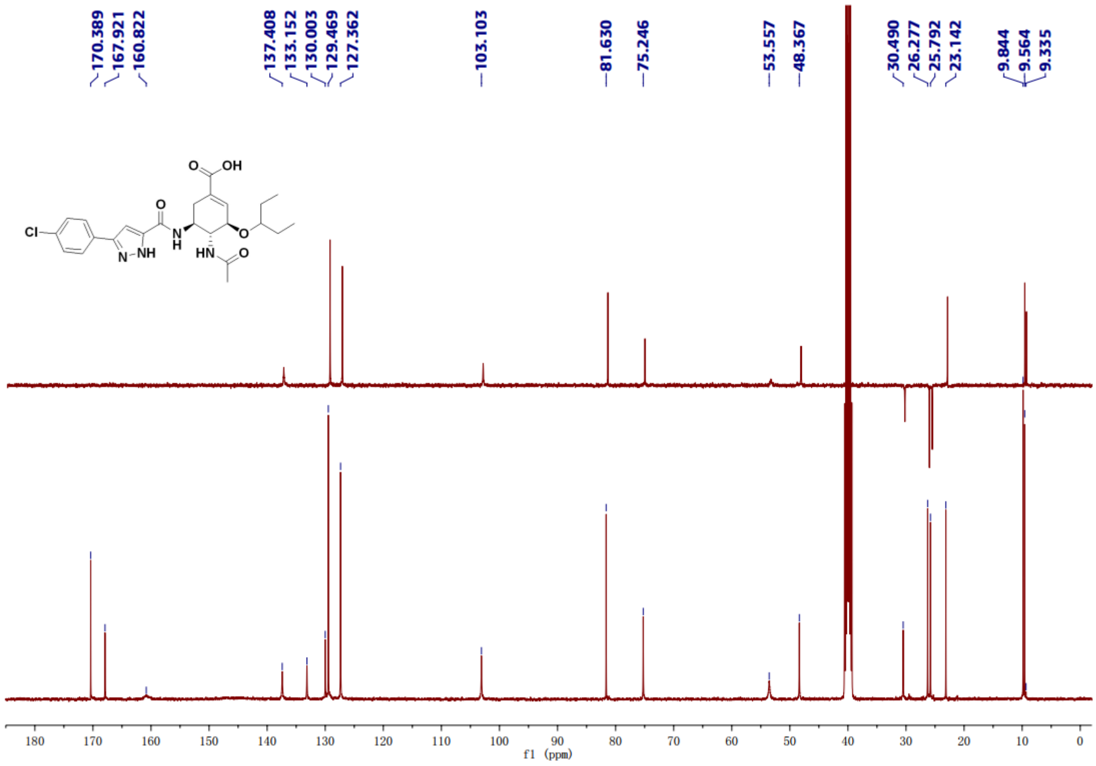

*(3R,4R,5S)-4-acetamido-5-(3-(4-fluorophenyl)-1H-pyrazole-5-carboxamido)-3-(pentan-3-yloxy)cyclohex-1-ene-1-carboxylic acid (**12c**)*

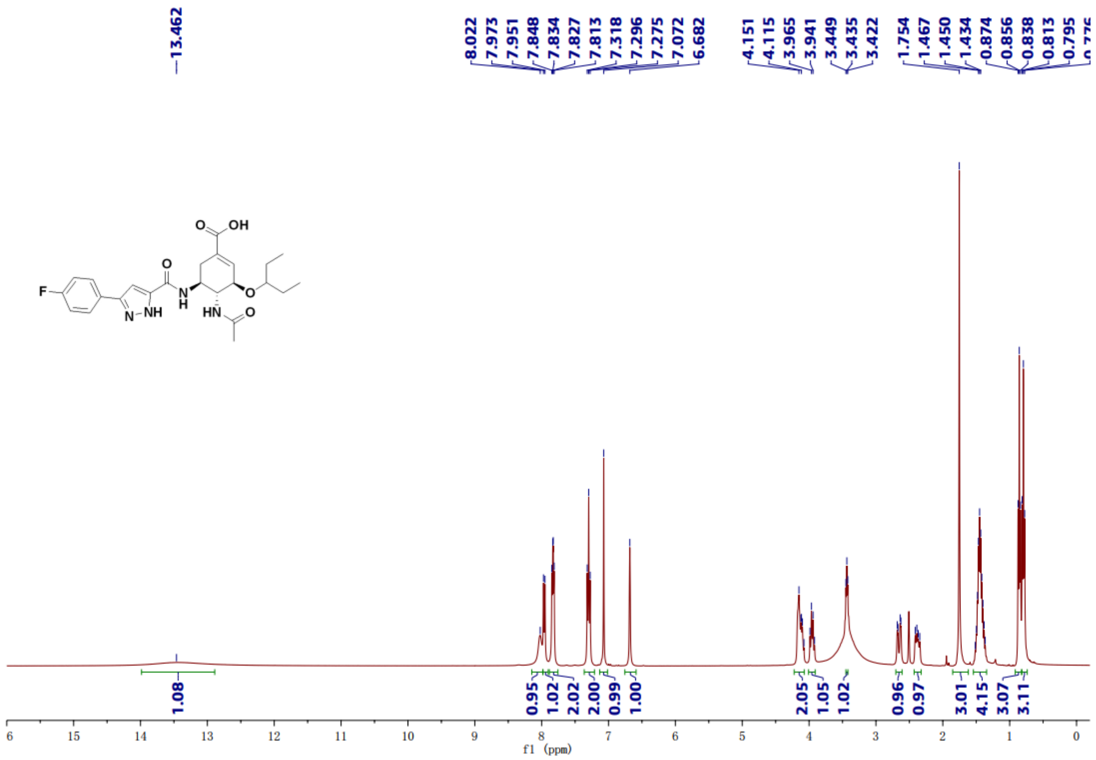

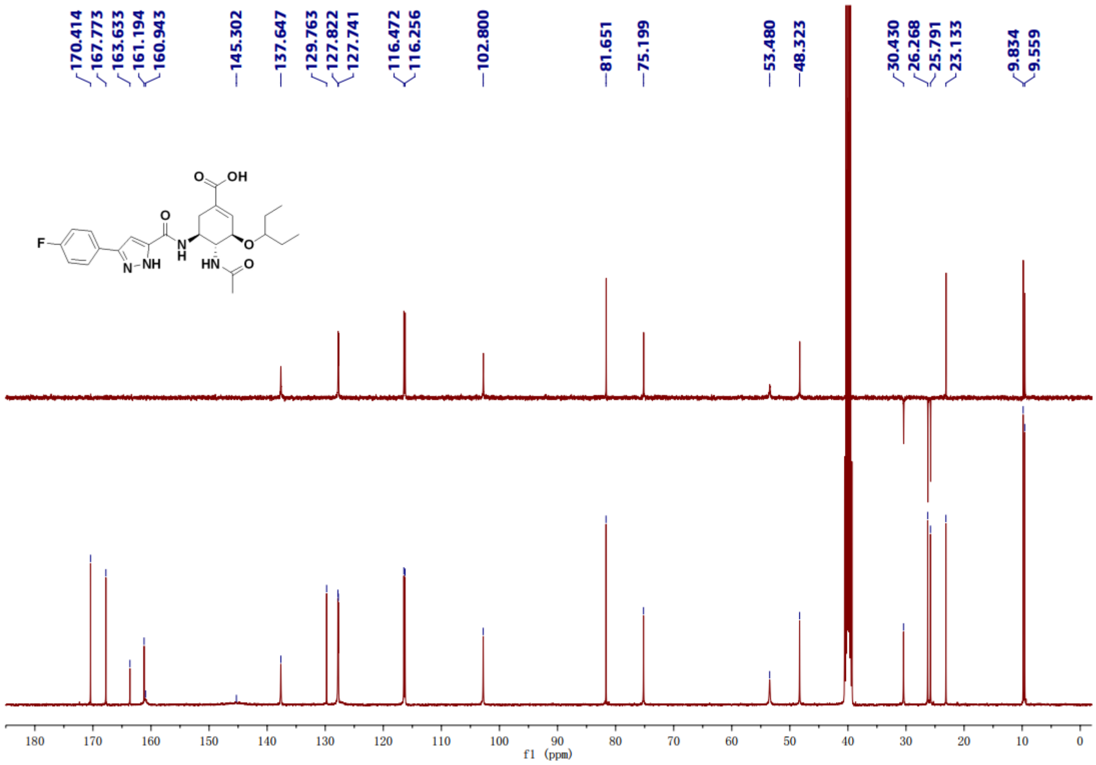

*(3R,4R,5S)-4-acetamido-3-(pentan-3-yloxy)-5-(3-(4-(trifluoromethyl)phenyl)-1H-pyrazole-5-carboxamido)cyclohex-1-ene-1-carboxylic acid (**12d**)*

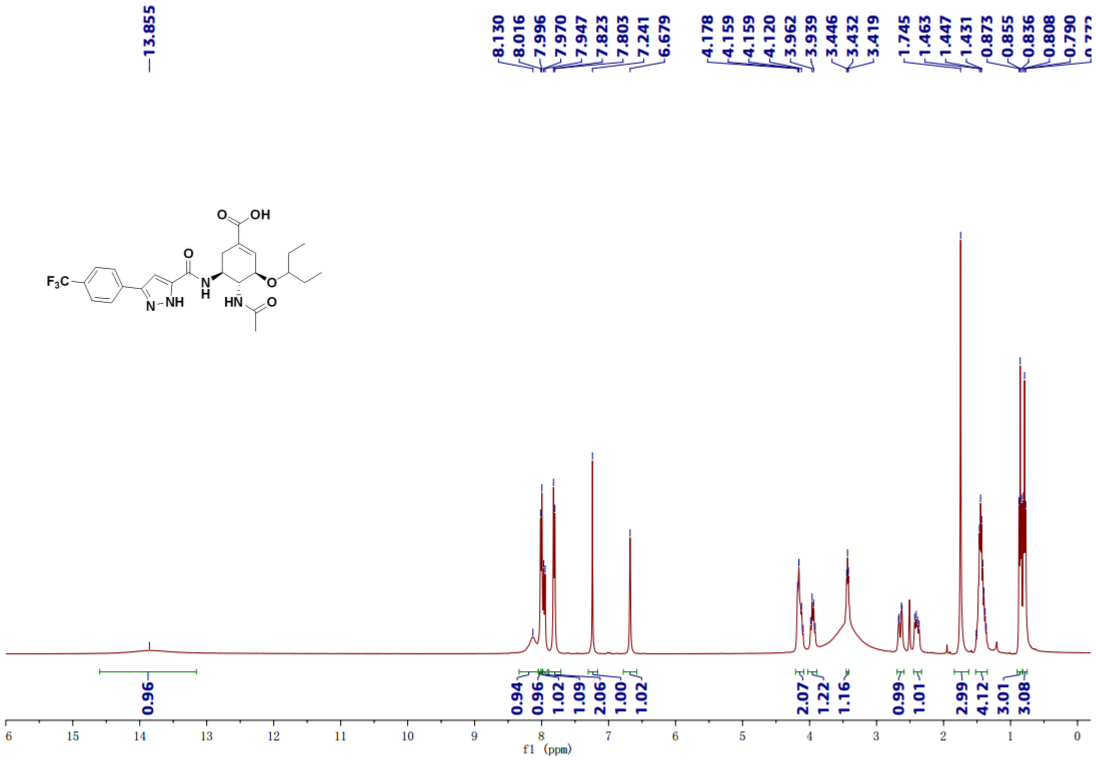

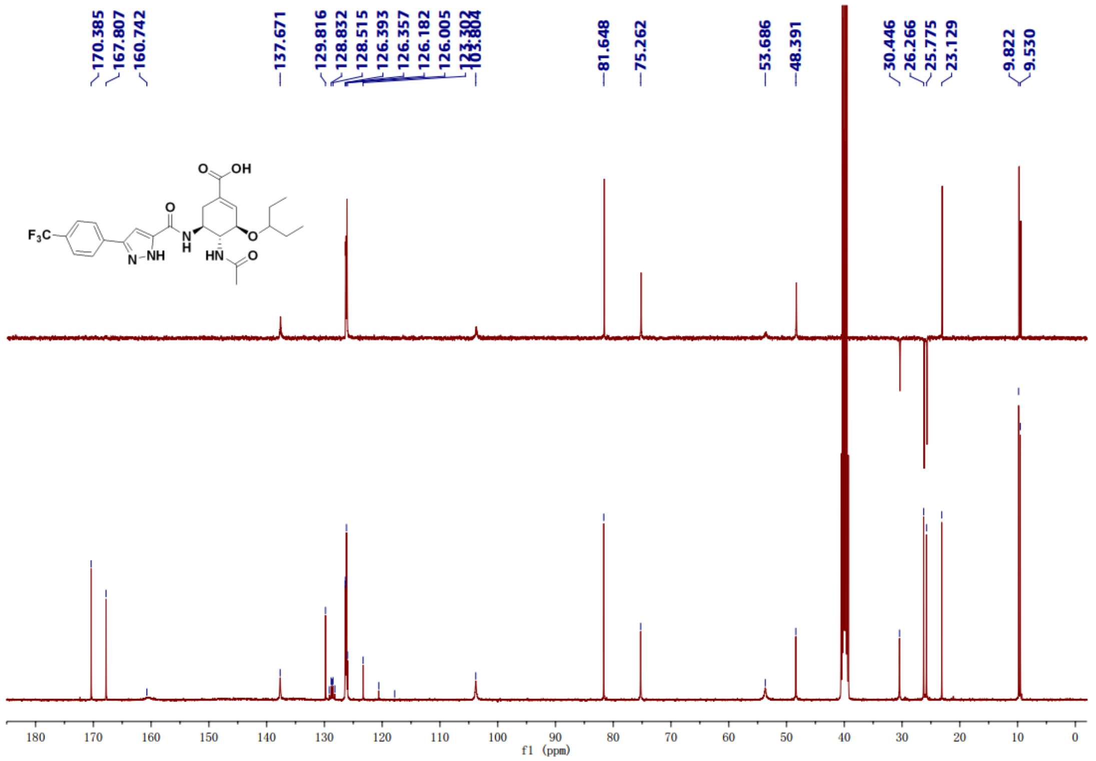

*(3R,4R,5S)-4-acetamido-5-(3-(4-methoxyphenyl)-1H-pyrazole-5-carboxamido)-3-(pentan-3-yloxy)cyclohex-1-ene-1-carboxylic acid (**12e**)*

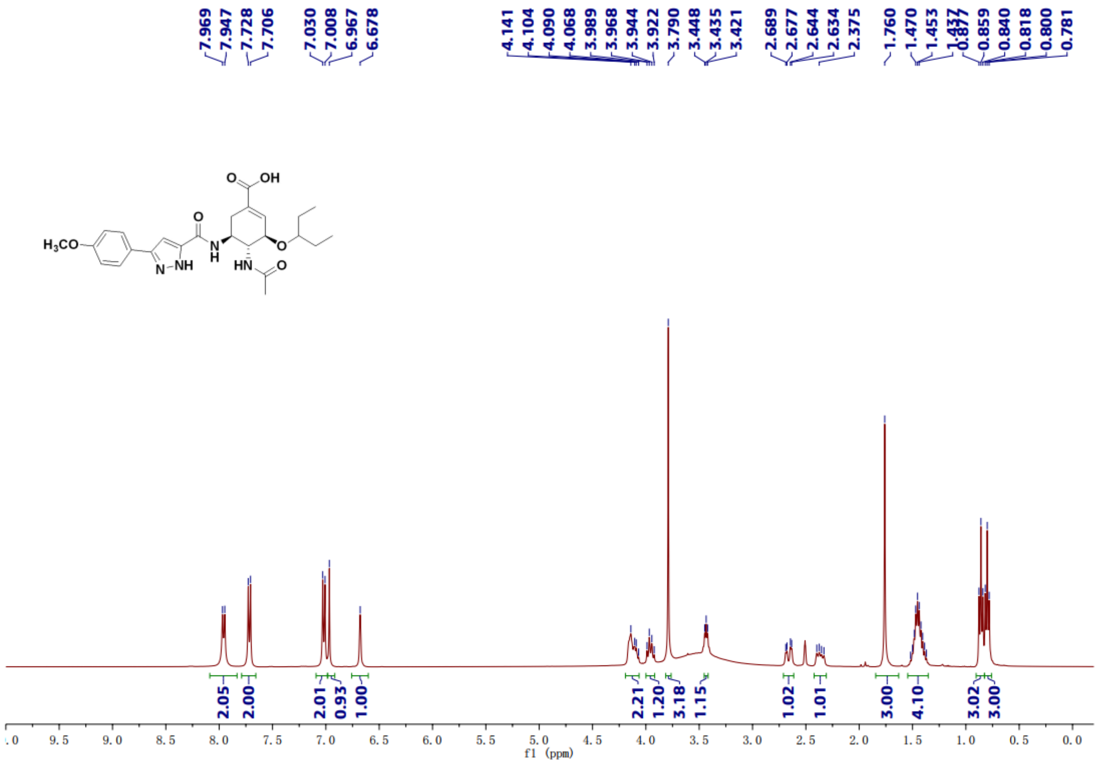

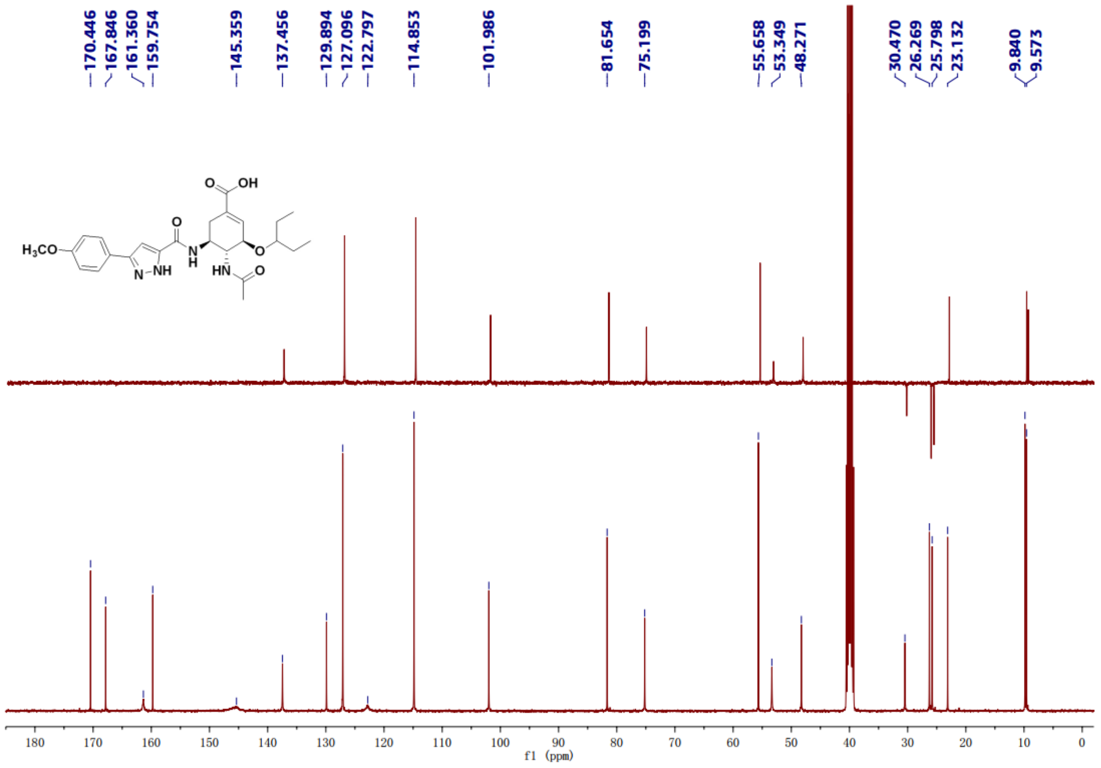

*(3R,4R,5S)-4-acetamido-3-(pentan-3-yloxy)-5-(3-(thiophen-2-yl)-1H-pyrazole-5-carboxamido)cyclohex-1-ene-1-carboxylic acid (**12f**)*

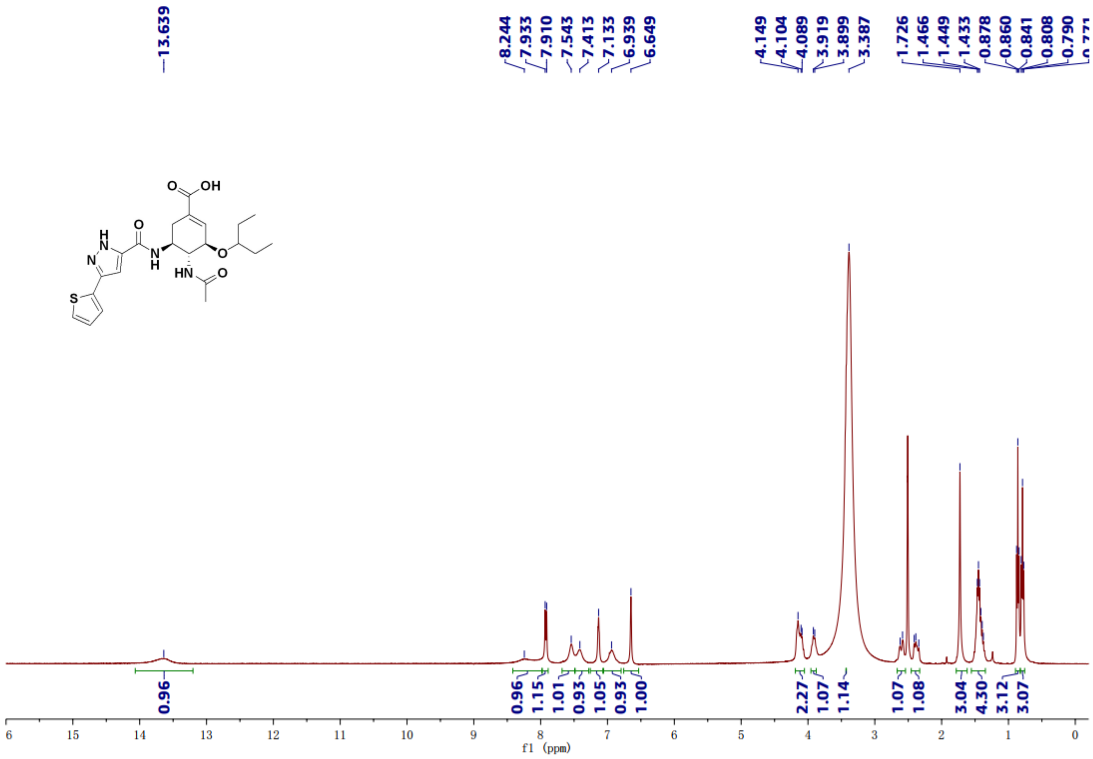

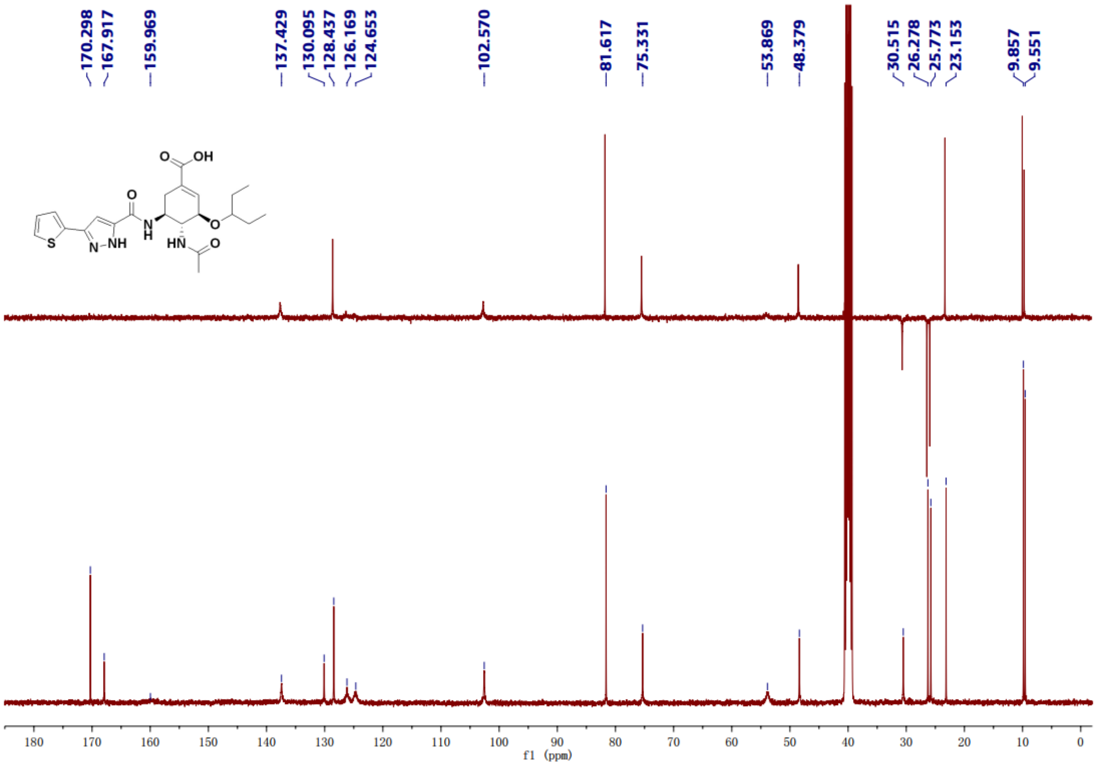

*(3R,4R,5S)-4-acetamido-5-(3-(4-chlorophenyl)-1-(cyclopropylmethyl)-1H-pyrazole-5-carboxamido)-3-(pentan-3-yloxy)cyclohex-1-ene-1-carboxylic acid (**16a**)*

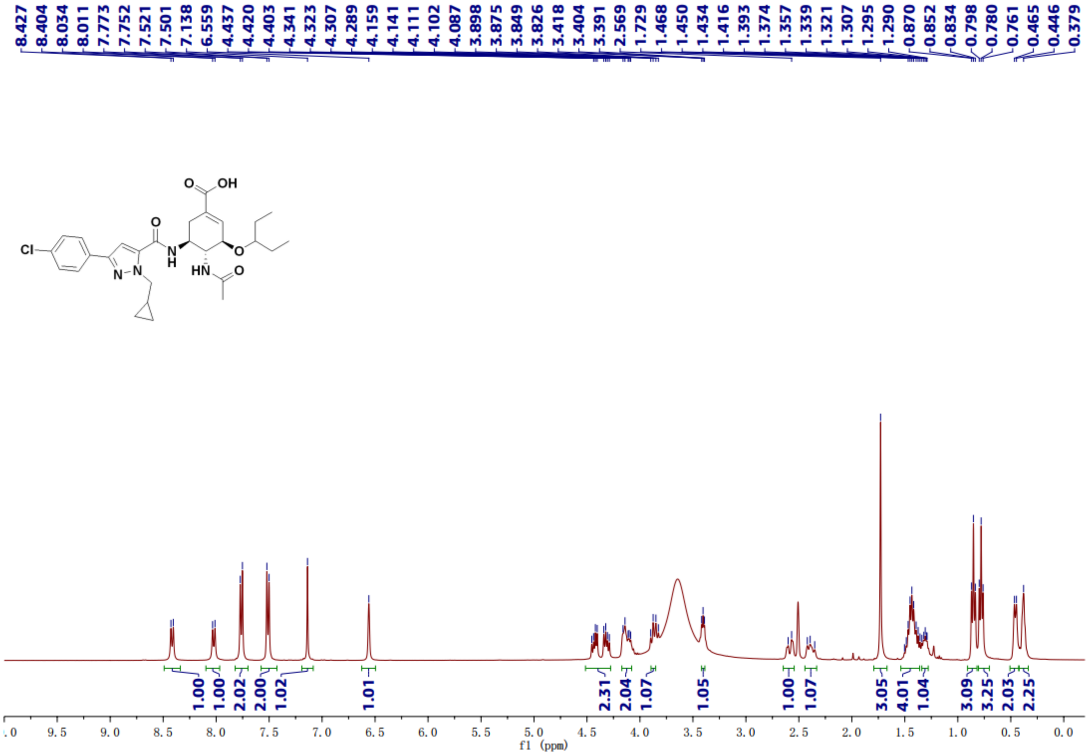

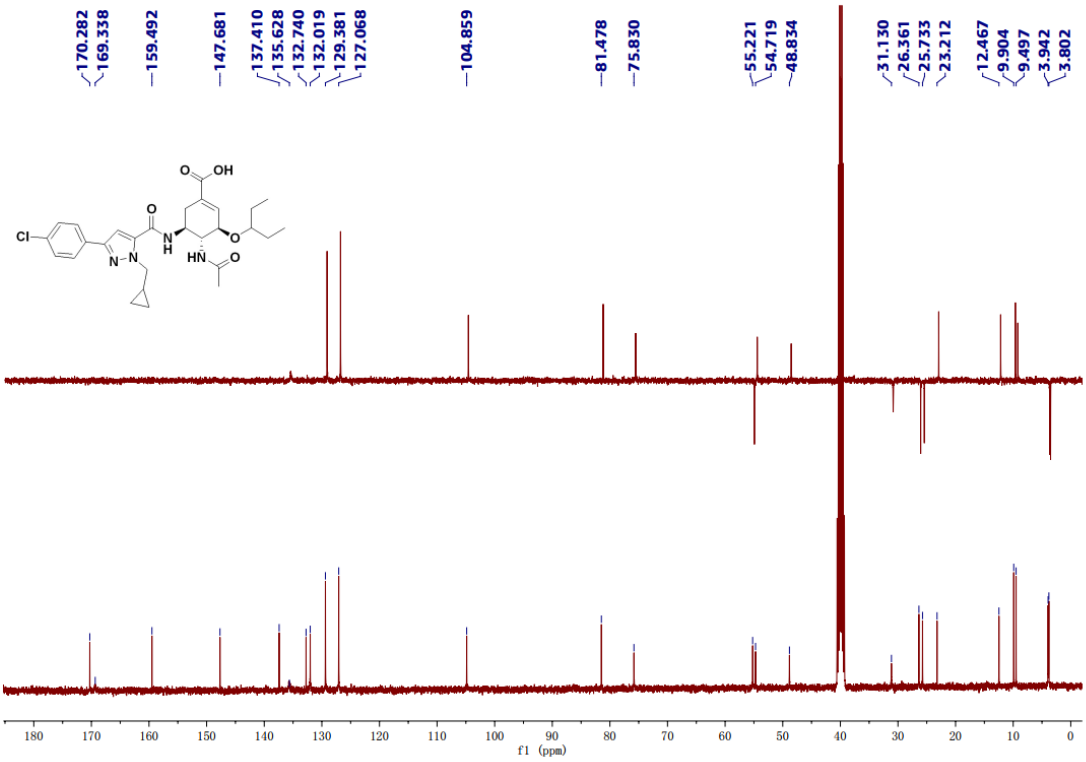

*(3R,4R,5S)-4-acetamido-5-(3-(4-chlorophenyl)-1-(cyclobutylmethyl)-1H-pyrazole-5-carboxamido)-3-(pentan-3-yloxy)cyclohex-1-ene-1-carboxylic acid (**16b**)*

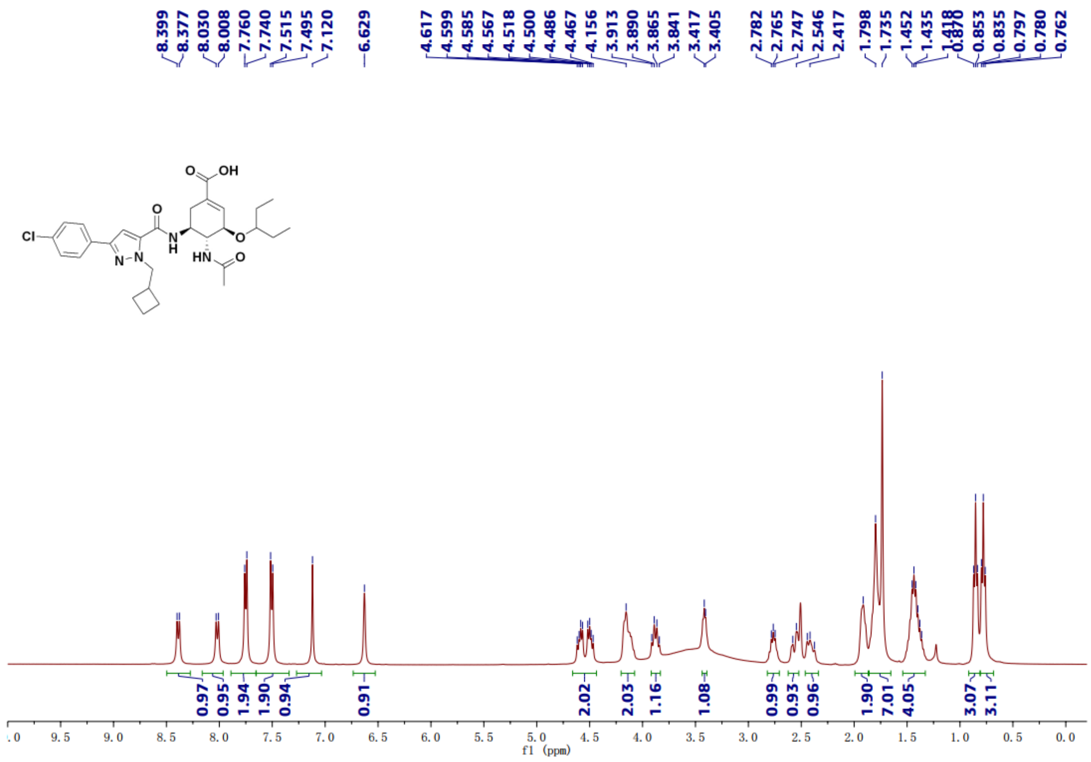

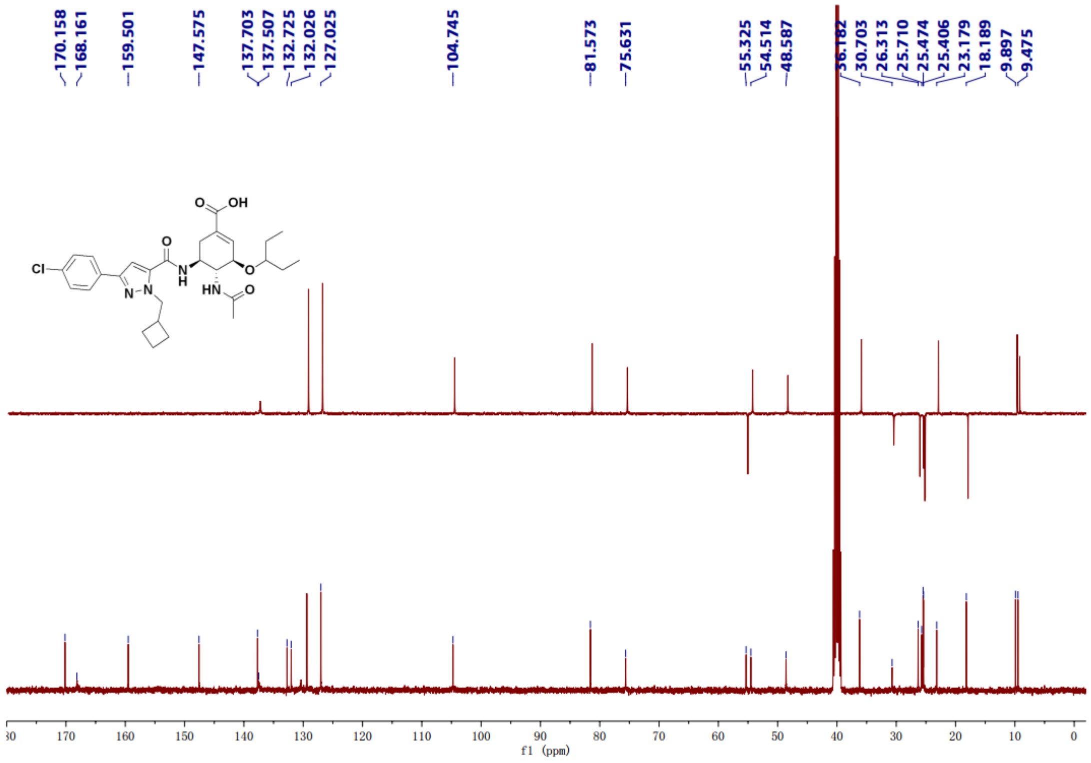

*(3R,4R,5S)-4-acetamido-5-(3-(4-chlorophenyl)-1-(cyclohexylmethyl)-1H-pyrazole-5-carboxamido)-3-(pentan-3-yloxy)cyclohex-1-ene-1-carboxylic acid (**16c**)*

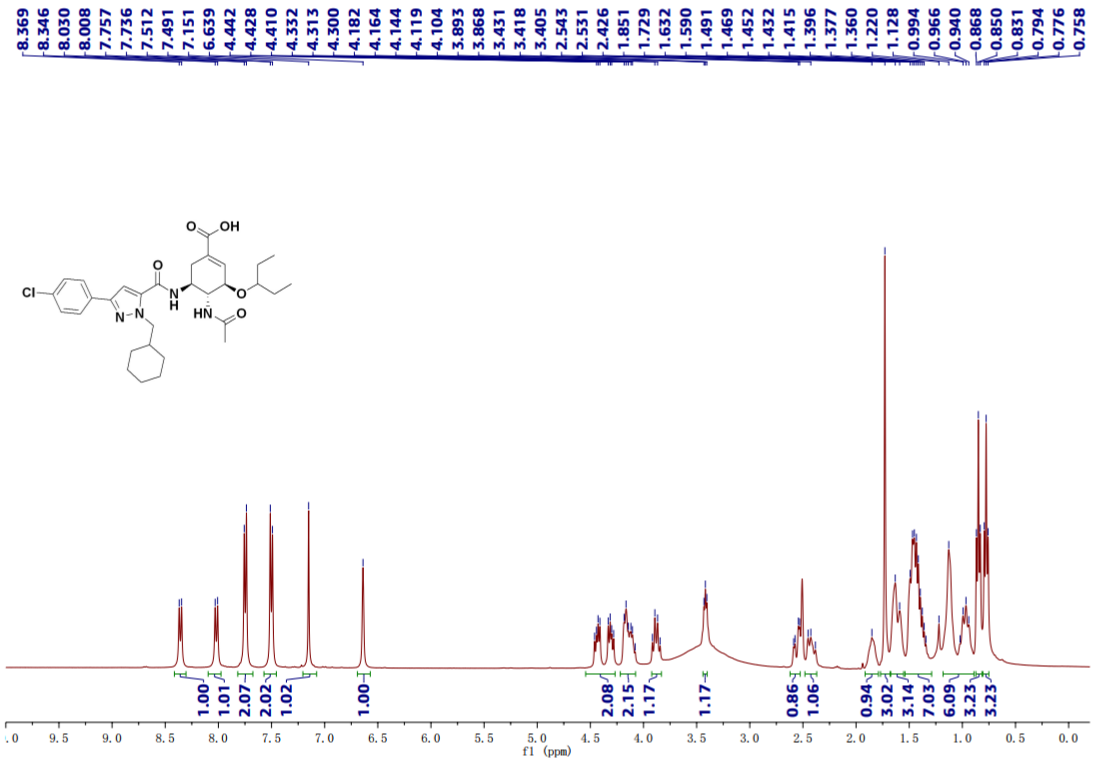

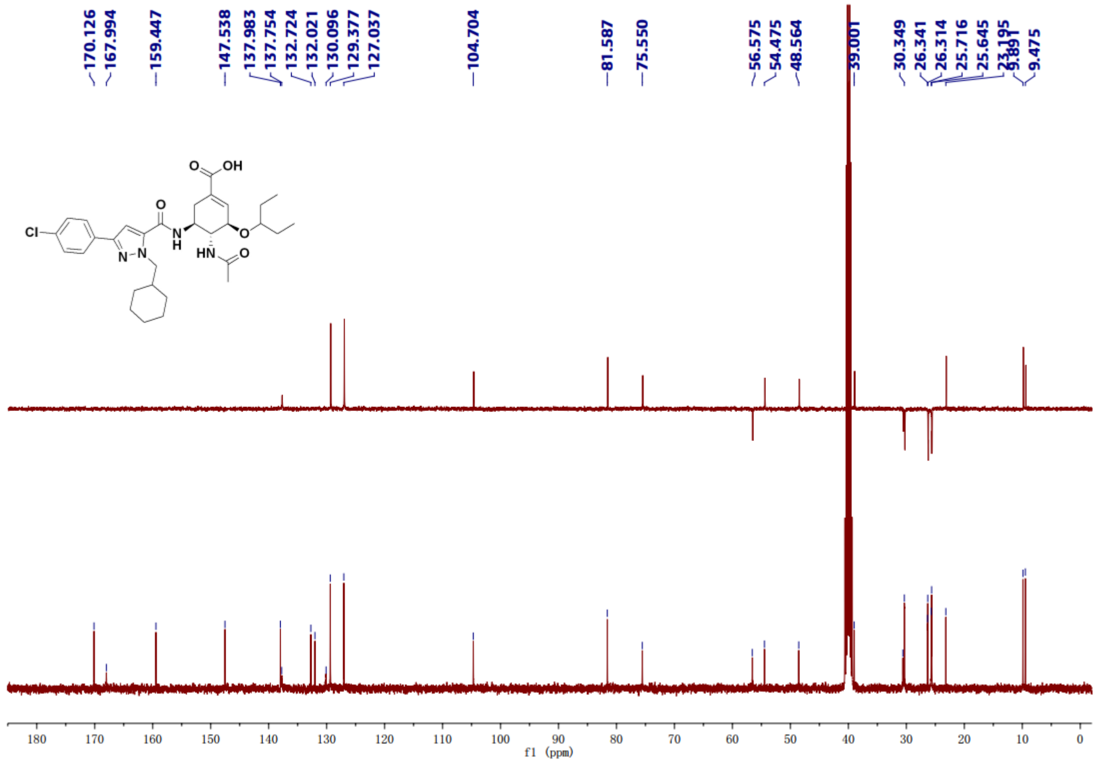

*(3R,4R,5S)-4-acetamido-5-(5-cyclopropyl-1-phenyl-1H-pyrazole-3-carboxamido)-3-(pentan-3-yloxy)cyclohex-1-ene-1-carboxylic acid (**24a**)*

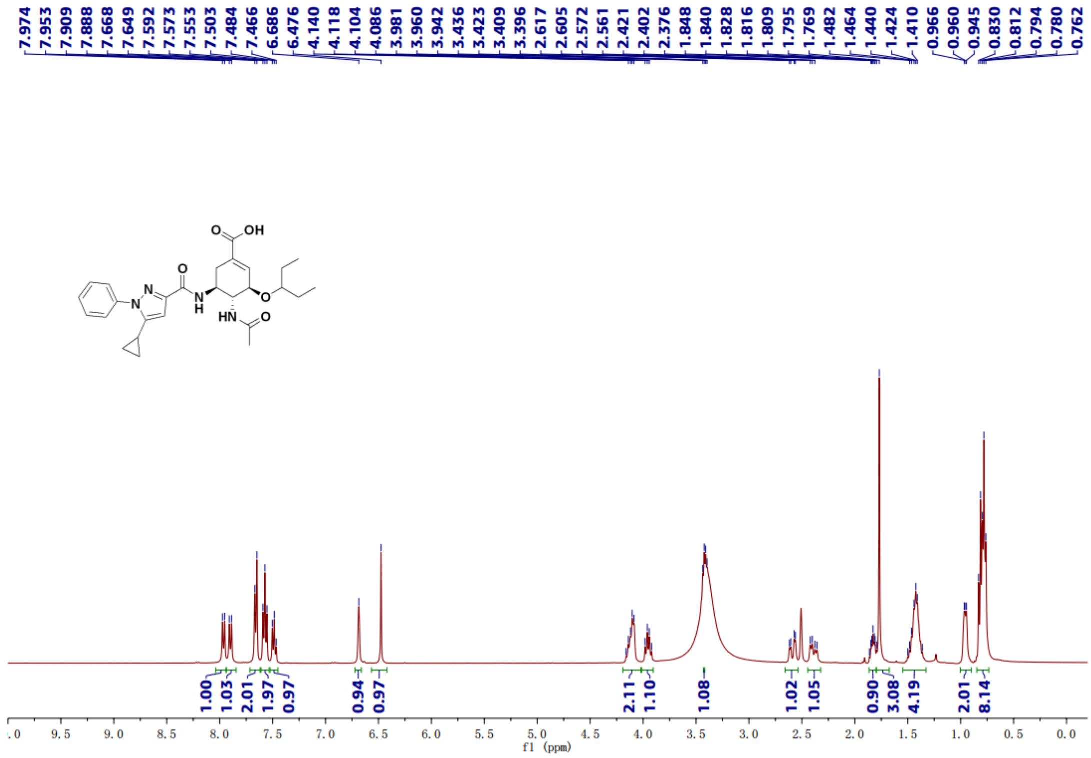

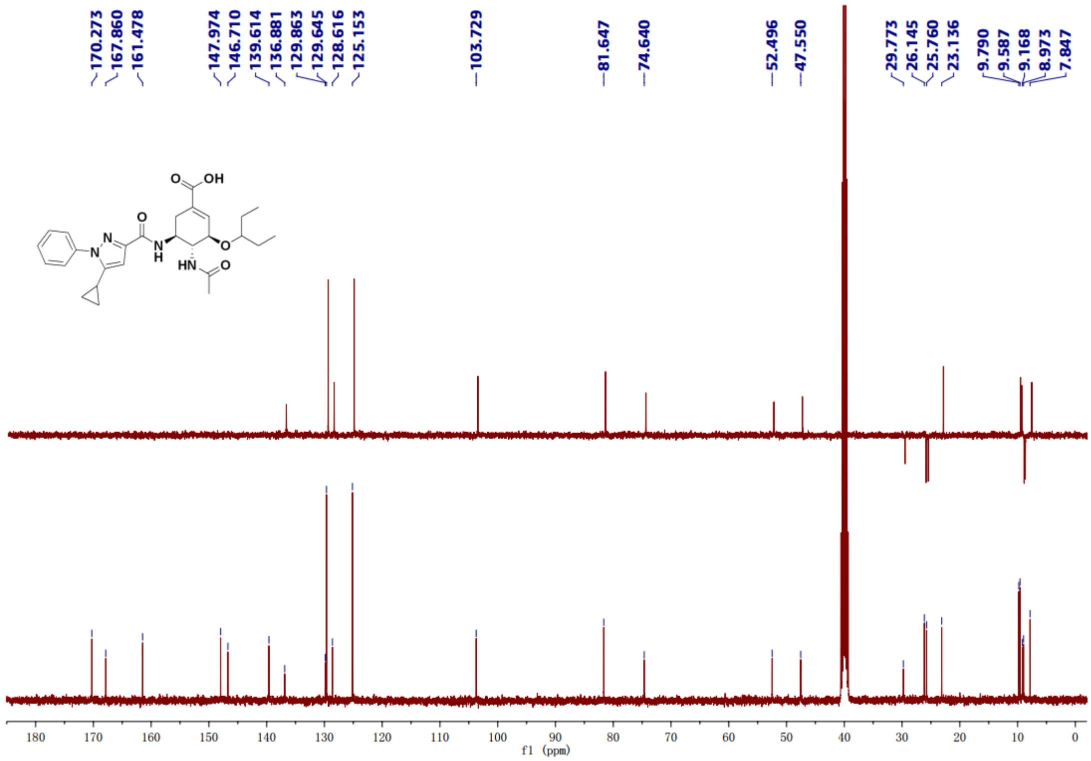

(3R,4R,5S)-4-acetamido-5-(5-cyclopropyl-1-(o-tolyl)-1H-pyrazole-3-carboxamido)-3-(pentan-3-yloxy)cyclohex-1-ene-1-carboxylic acid (***24b***)

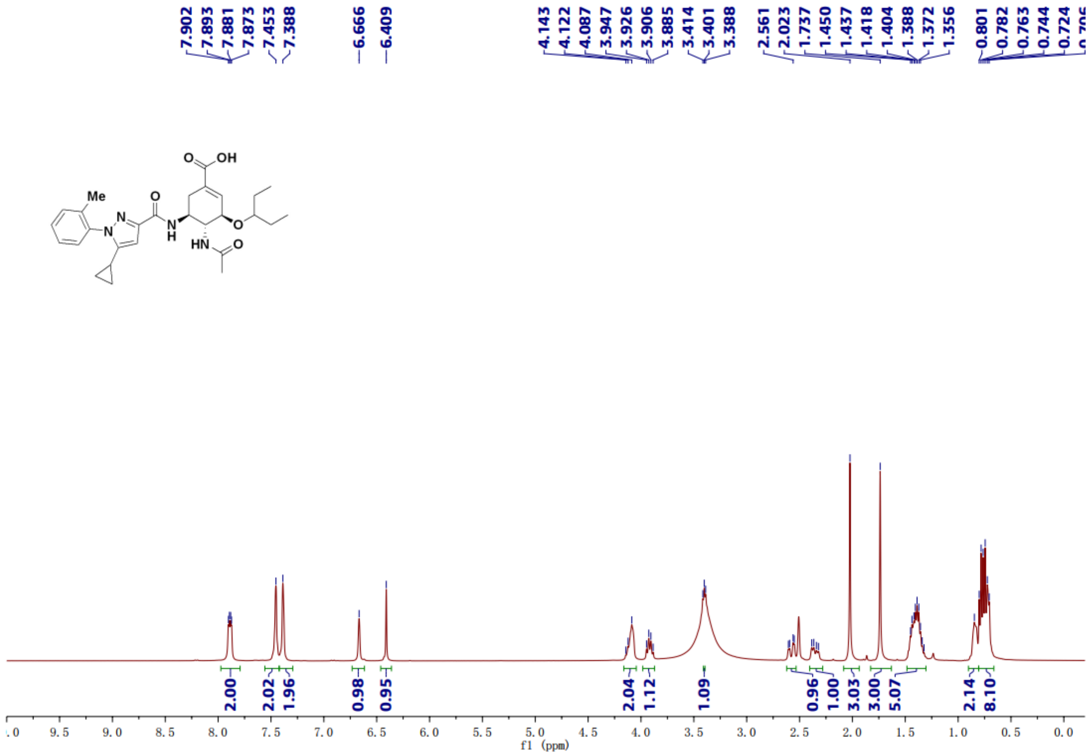

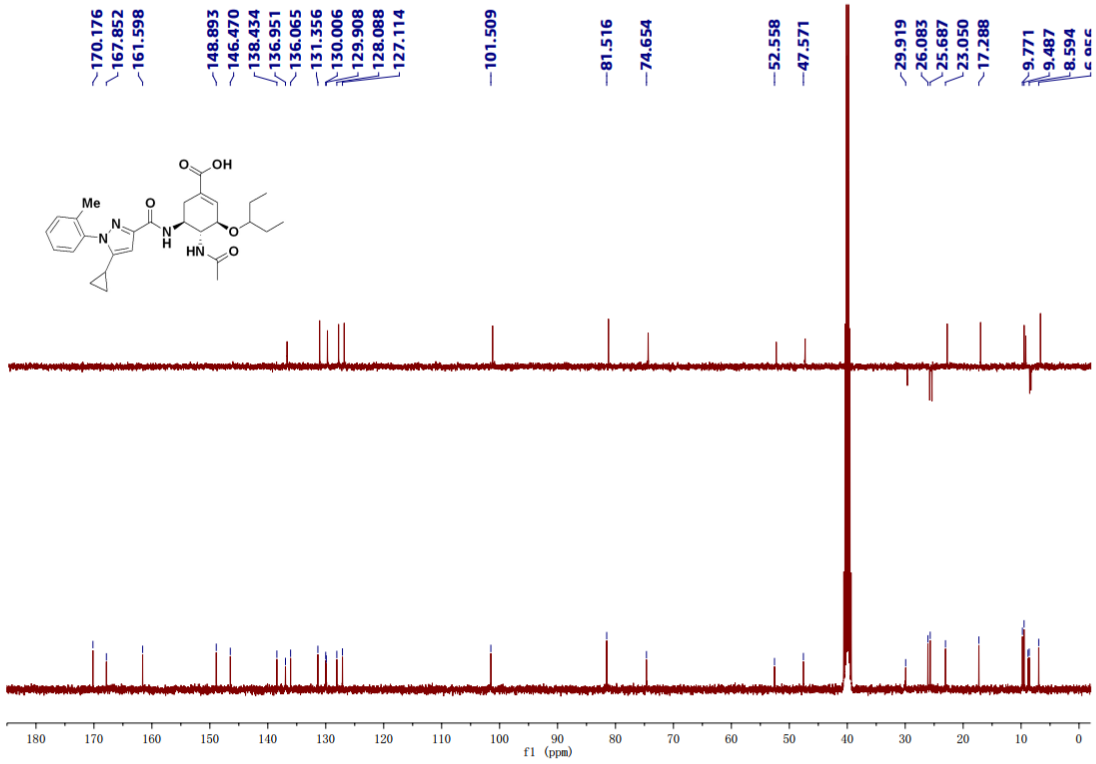

*(3R,4R,5S)-4-acetamido-5-(5-cyclopropyl-1-(m-tolyl)-1H-pyrazole-3-carboxamido)-3-(pentan-3-yloxy)cyclohex-1-ene-1-carboxylic acid (**24c**)*

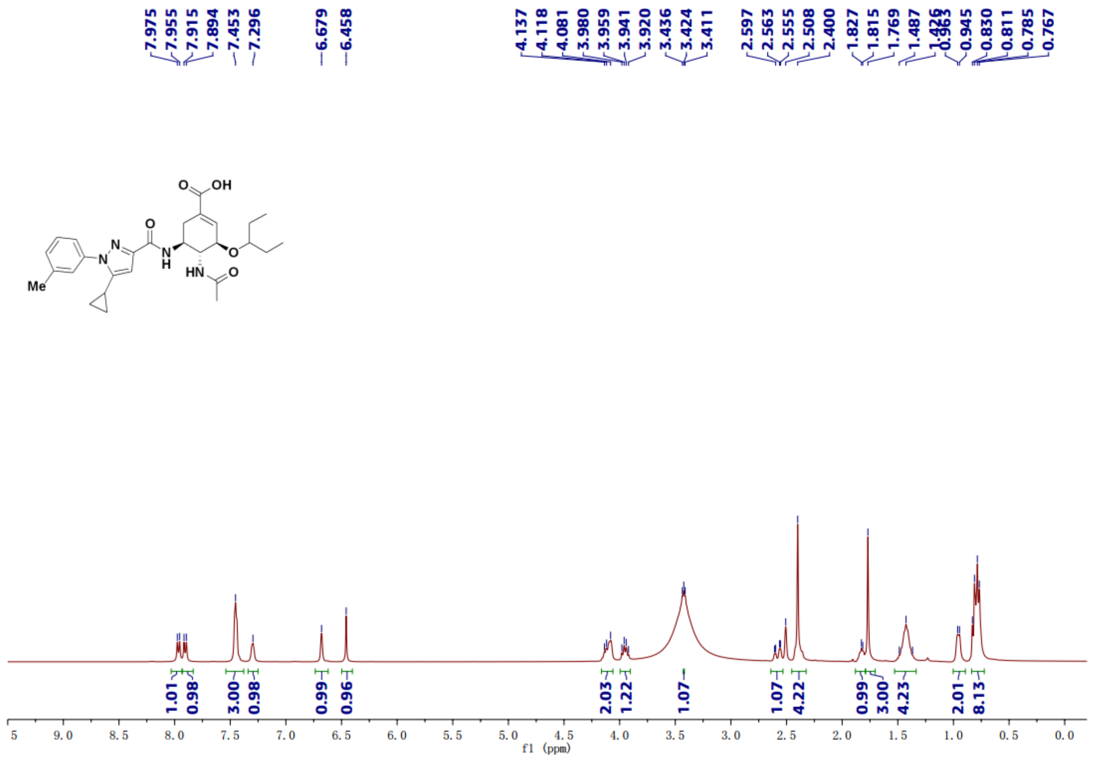

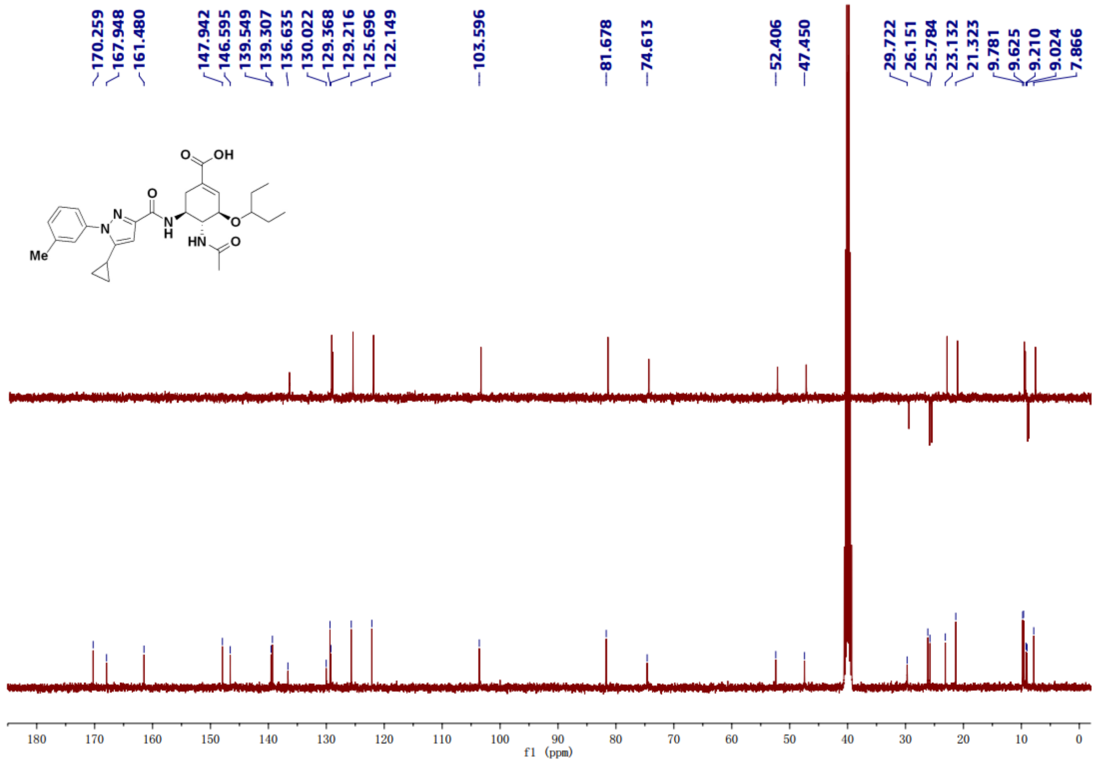

*(3R,4R,5S)-4-acetamido-5-(1-(4-bromophenyl)-5-cyclopropyl-1H-pyrazole-3-carboxamido)-3-(pentan-3-yloxy)cyclohex-1-ene-1-carboxylic acid (**24d**)*

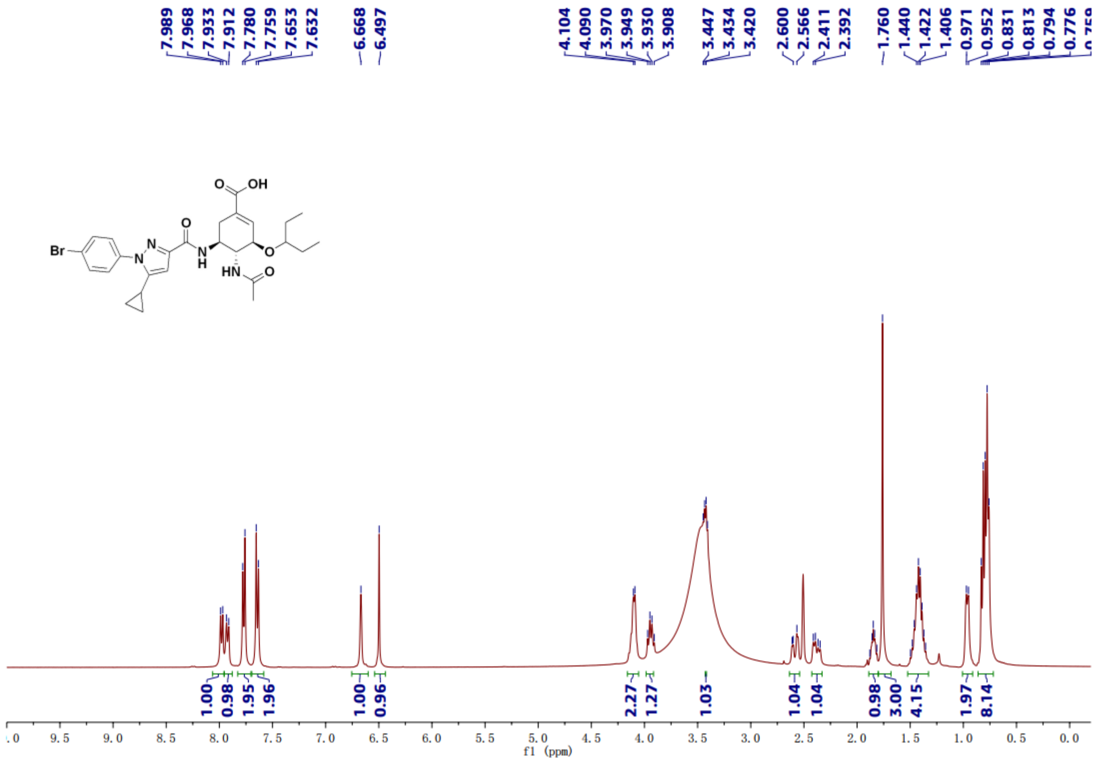

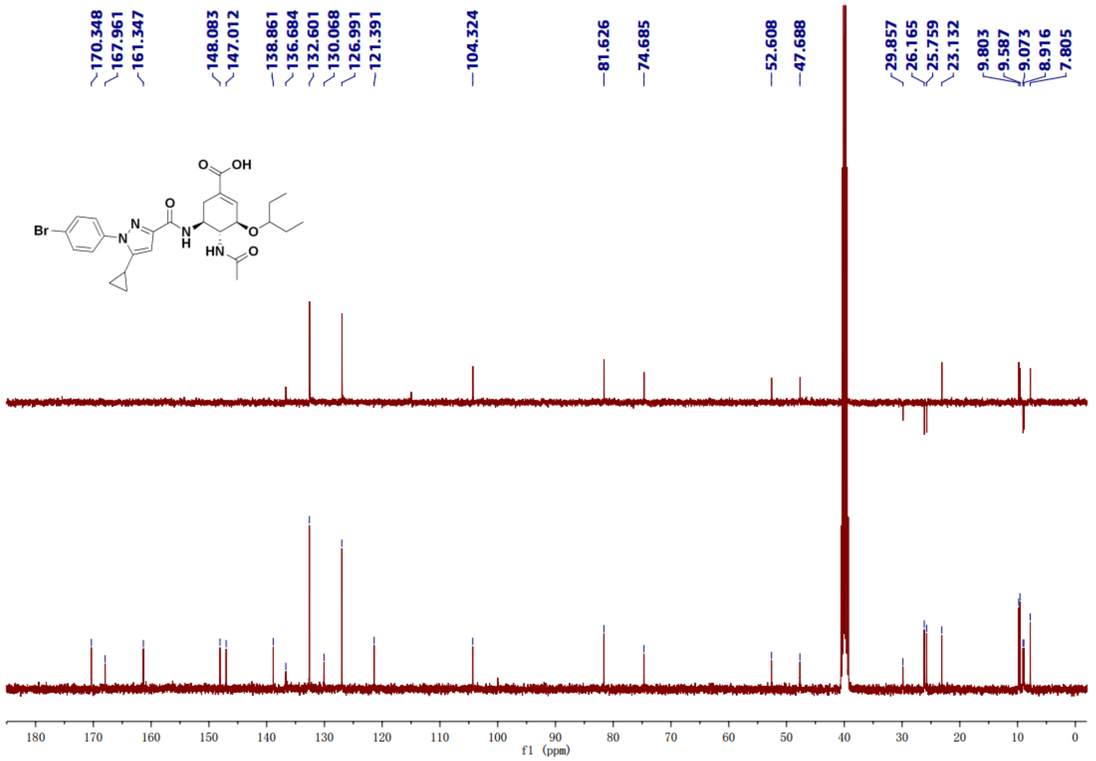

*(3R,4R,5S)-4-acetamido-5-(5-cyclopropyl-1-(4-(trifluoromethyl)phenyl)-1H-pyrazole-3-carboxamido)-3-(pentan-3-yloxy)cyclohex-1-ene-1-carboxylic acid (**24e**)*

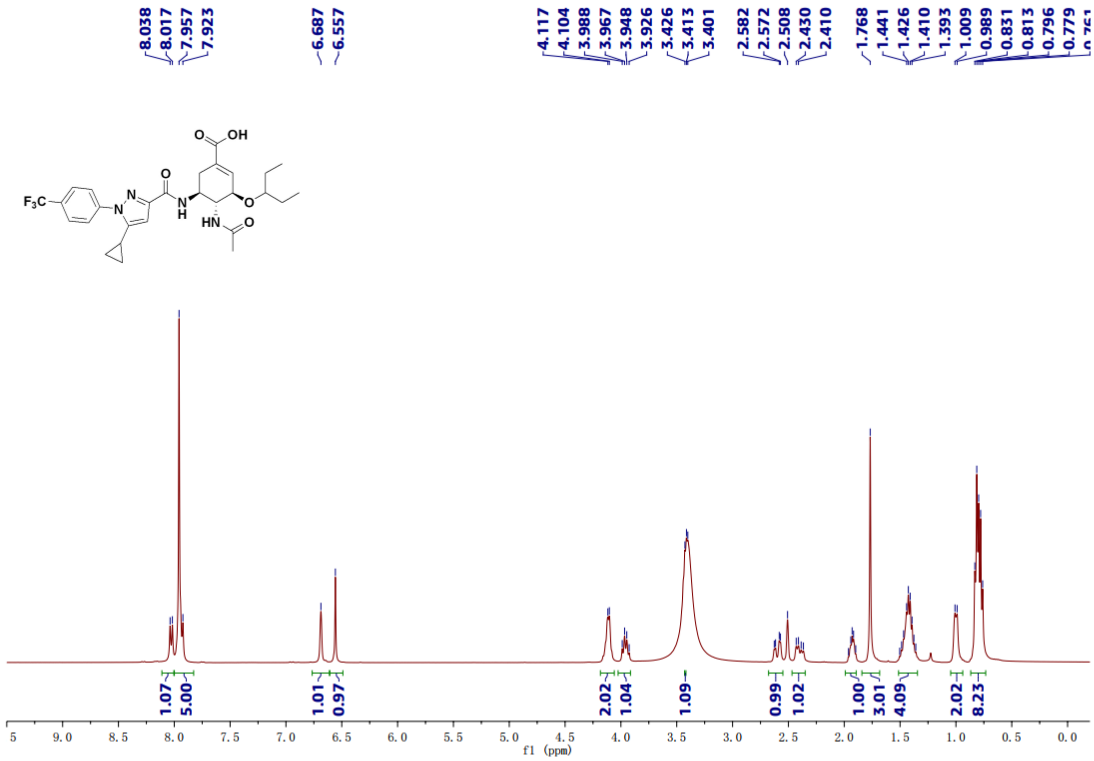

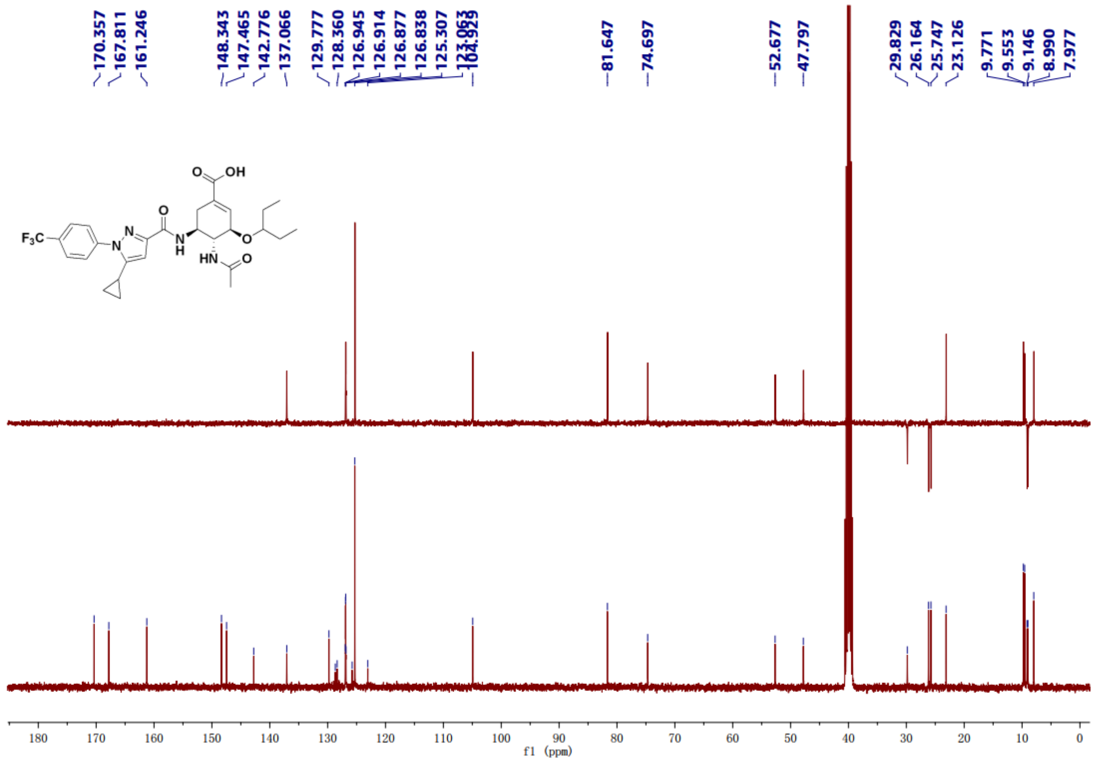

*(3R,4R,5S)-4-acetamido-5-(3-cyclopropyl-1-phenyl-1H-pyrazole-5-carboxamido)-3-(pentan-3-yloxy)cyclohex-1-ene-1-carboxylic acid (**26a**)*

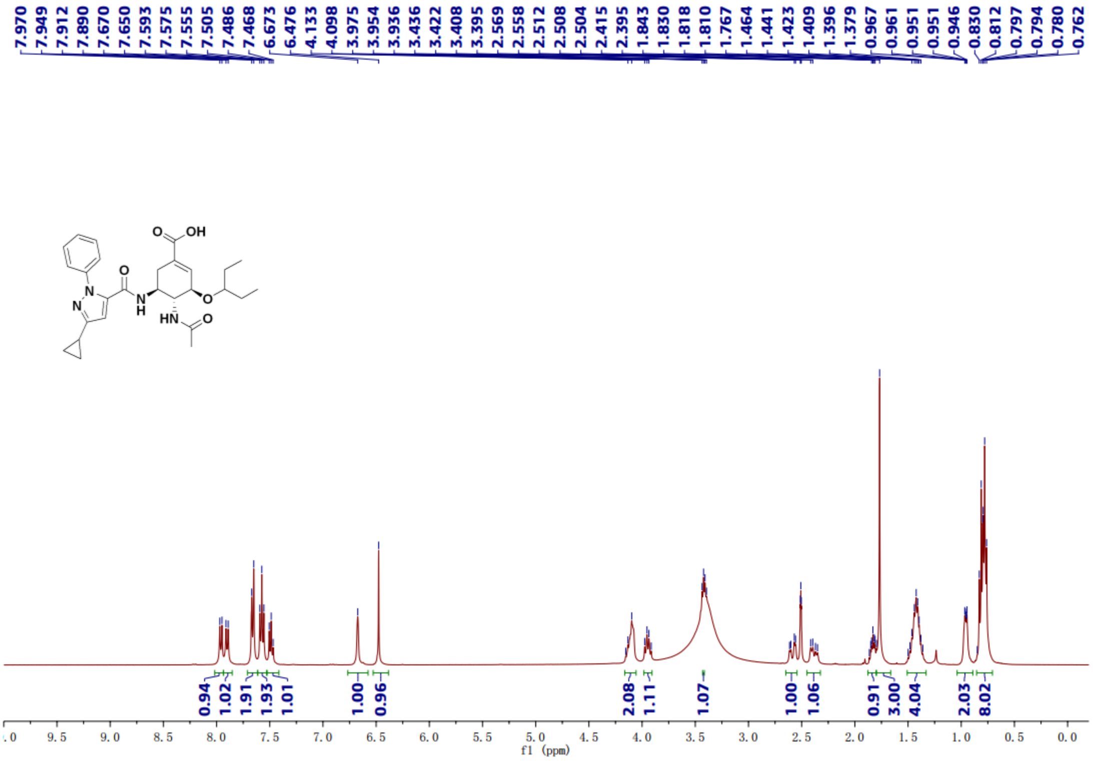

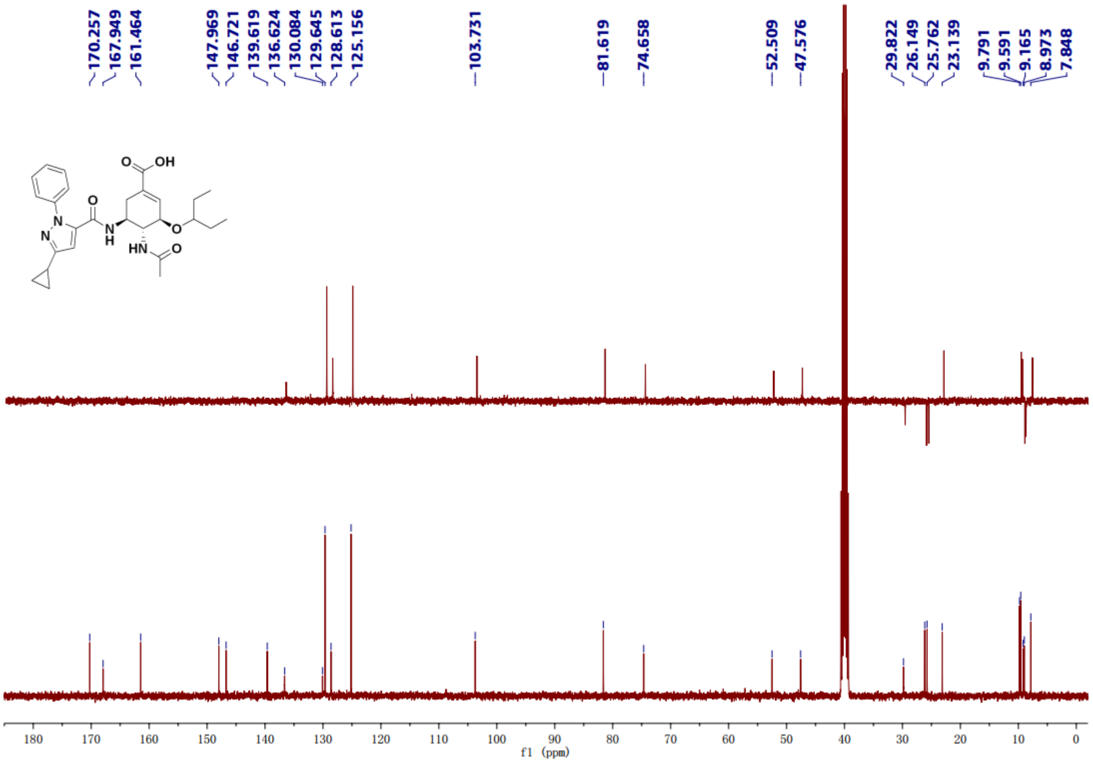

*(3R,4R,5S)-4-acetamido-5-(3-cyclopropyl-1-(o-tolyl)-1H-pyrazole-5-carboxamido)-3-(pentan-3-yloxy)cyclohex-1-ene-1-carboxylic acid (**26b**)*

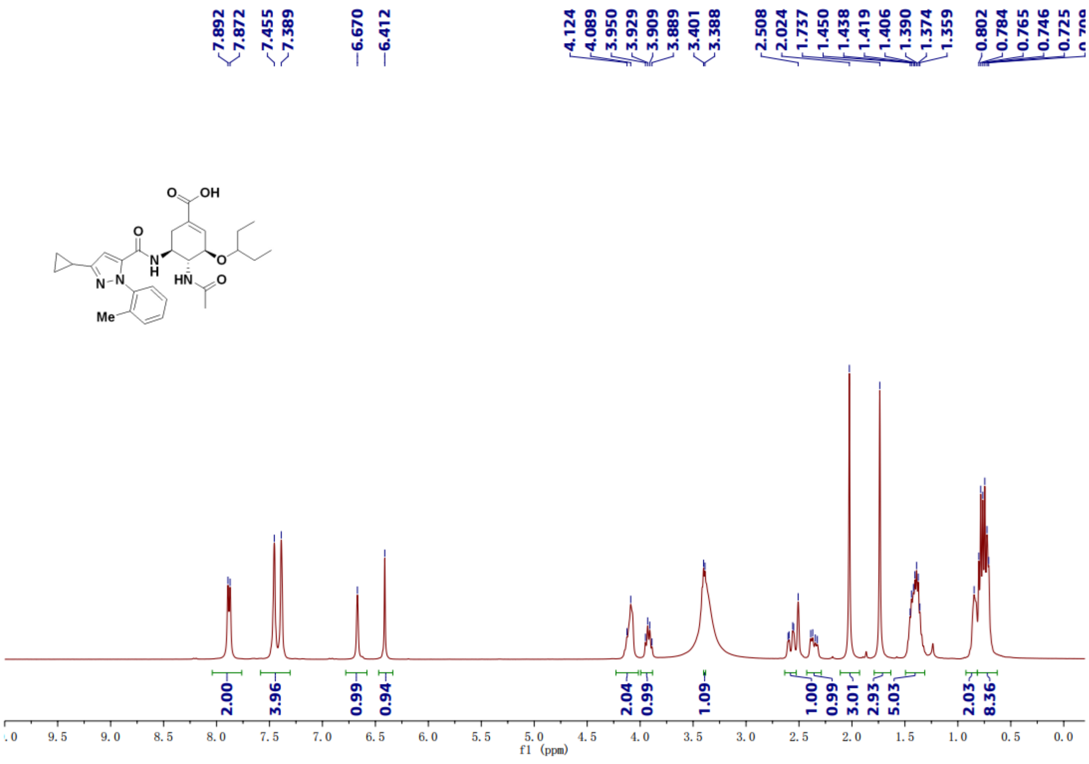

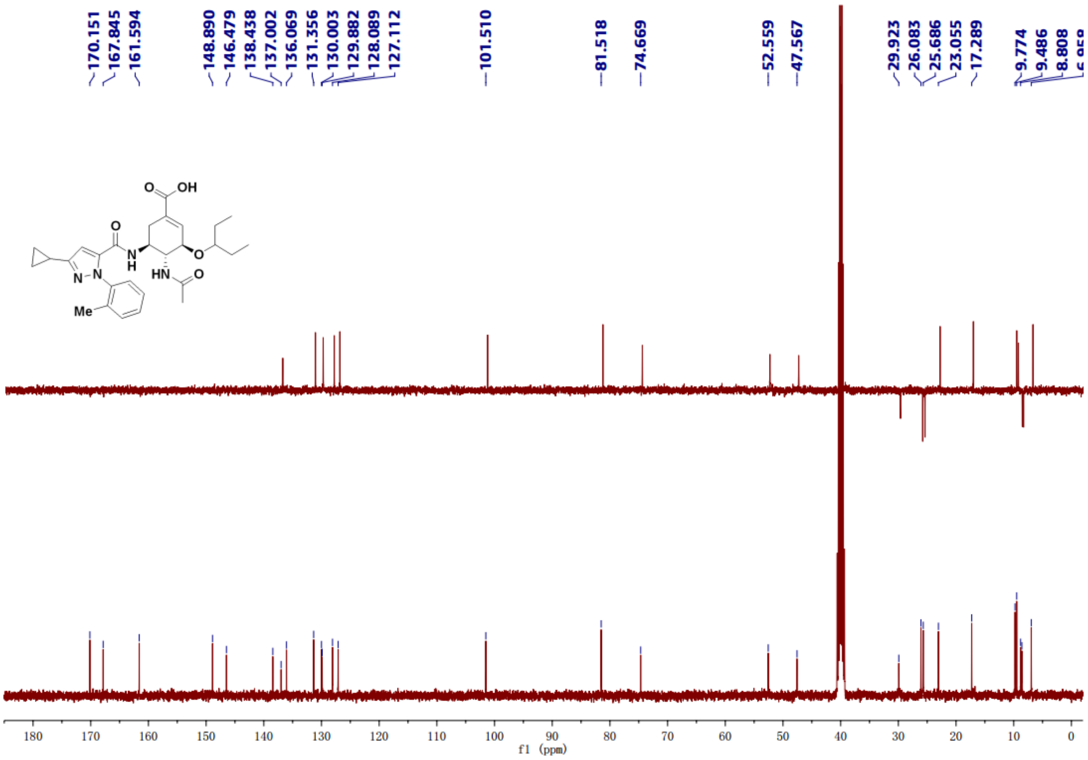

*(3R,4R,5S)-4-acetamido-5-(3-cyclopropyl-1-(m-tolyl)-1H-pyrazole-5-carboxamido)-3-(pentan-3-yloxy)cyclohex-1-ene-1-carboxylic acid (**26c**)*

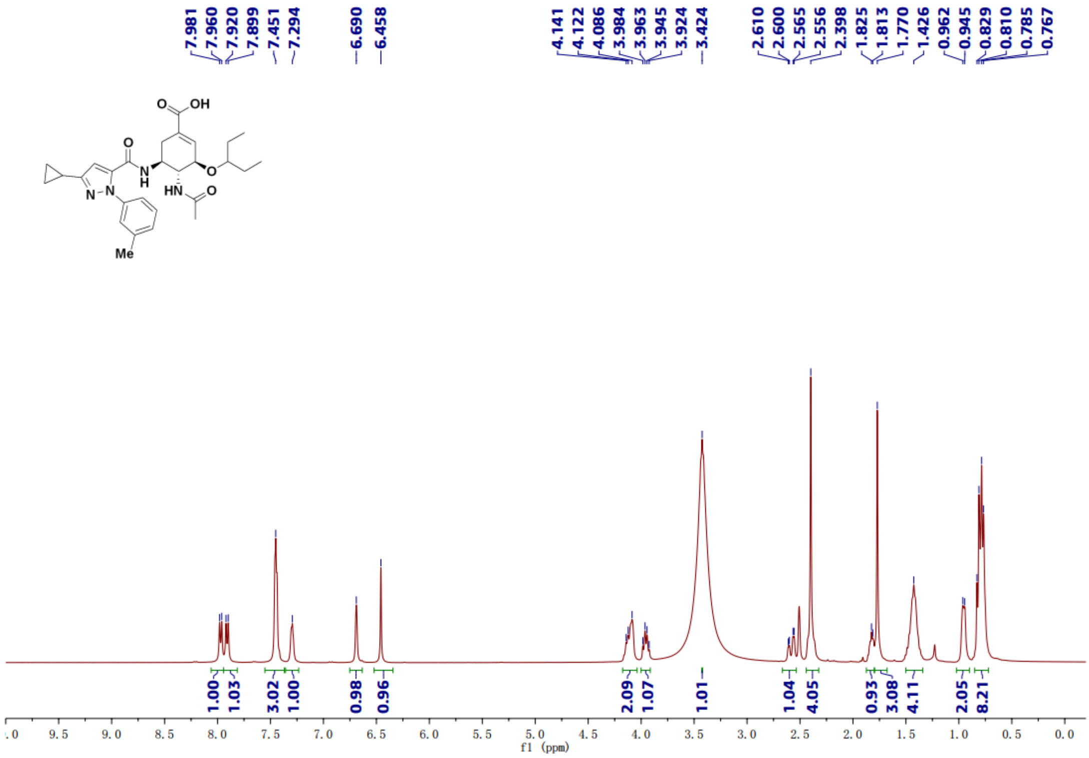

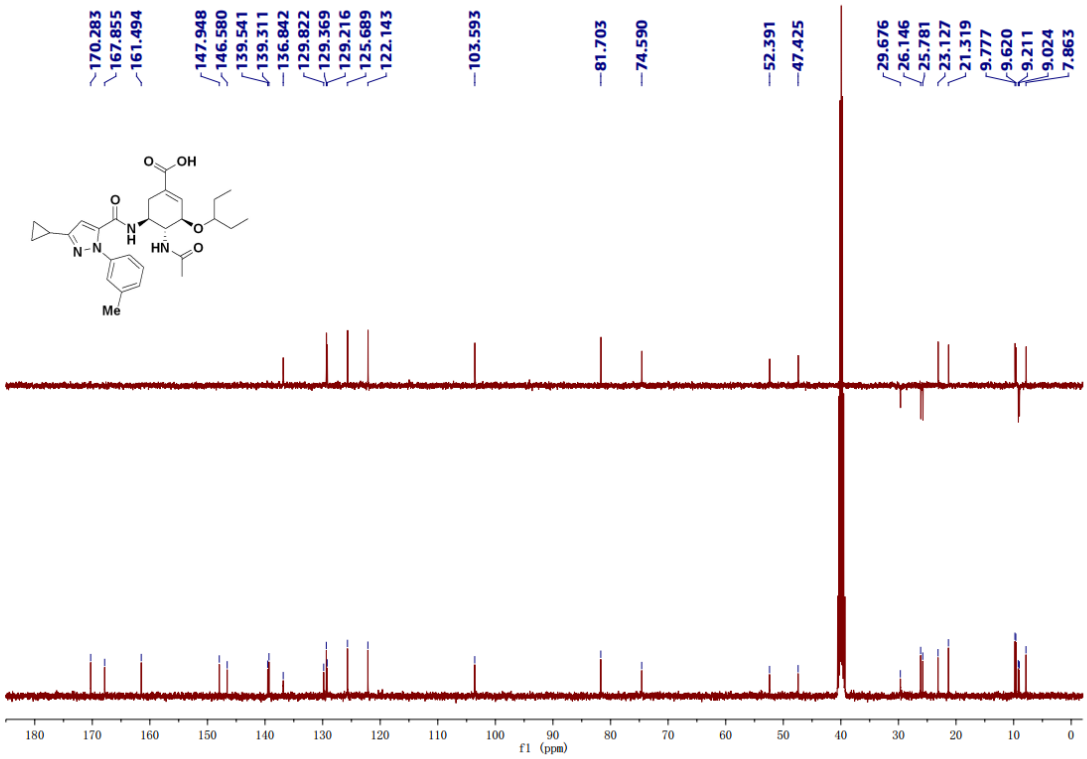

*(3R,4R,5S)-4-acetamido-5-(1-(4-bromophenyl)-3-cyclopropyl-1H-pyrazole-5-carboxamido)-3-(pentan-3-yloxy)cyclohex-1-ene-1-carboxylic acid (**26d**)*

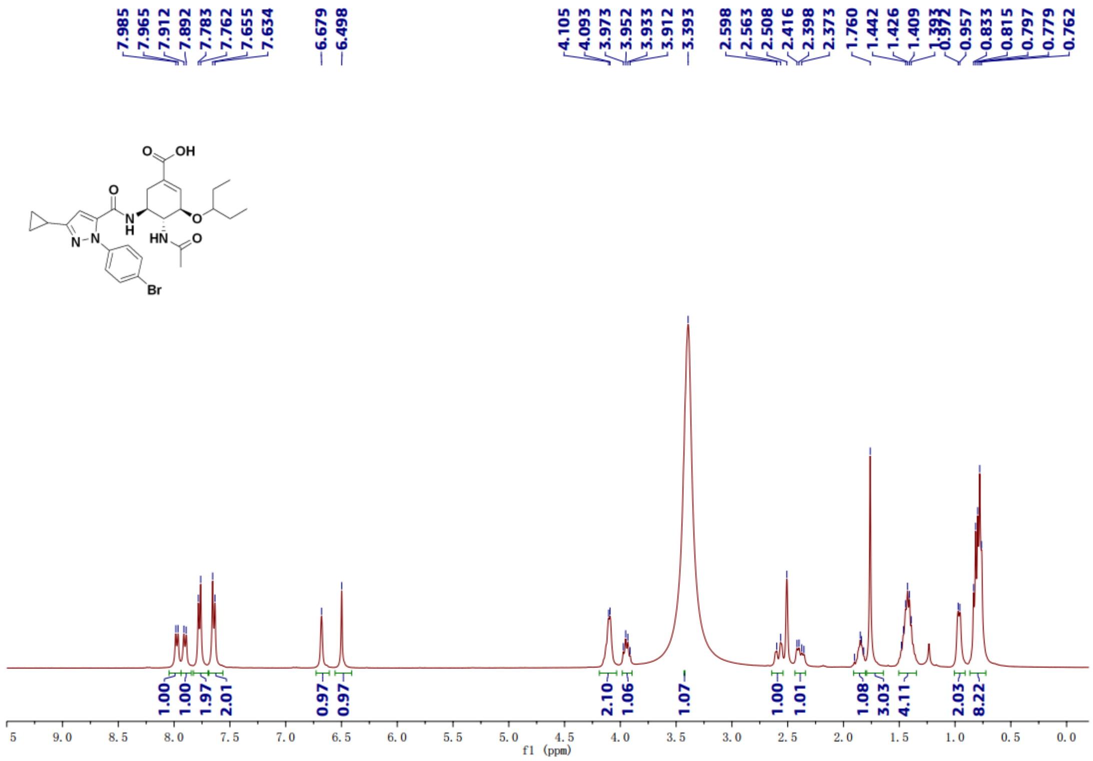

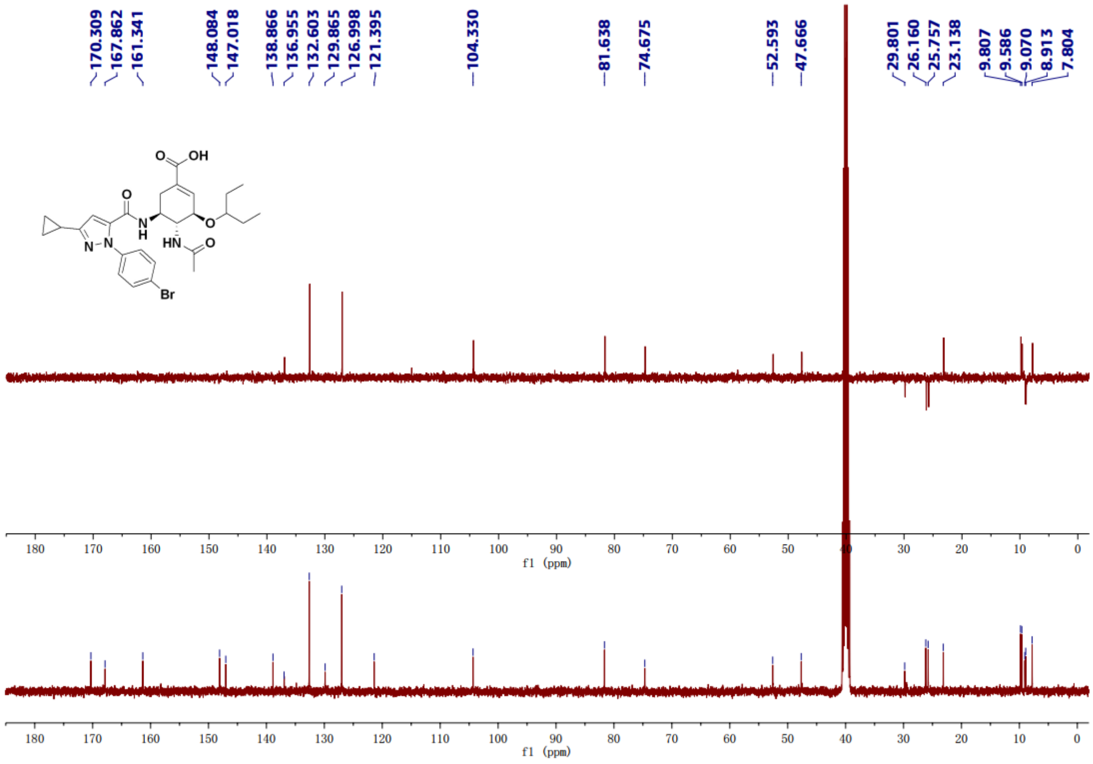

Supplement: Supplementary file 1 [file pharmaceuticals-14-00371-s001.zip › pharmaceuticals-1175640-supplementary.pdf]
